# Open-source customizable website to follow-up physical rehabilitation of cardiovascular patients at home

**DOI:** 10.3389/fcvm.2025.1633106

**Published:** 2025-07-25

**Authors:** Ariadna Sabala, Donough Mcbrearty, Raffaella Salama, Ramon Farré, Ailís Loughnane, Jorge Otero, Núria Farré

**Affiliations:** ^1^ Unit of Biophysics and Bioengineering, School of Medicine and Health Sciences, University of Barcelona, Barcelona, Spain; ^2^ Discipline of Cardiology, Saolta University Healthcare Group, Galway, Ireland; ^3^ CIBER de Enfermedades Respiratorias, Barcelona, Spain; ^4^ Institut d’Investigacions Biomèdiques August Pi Sunyer, Barcelona, Spain; ^5^ School of Medicine, University of Galway, Galway, Ireland

**Keywords:** cardiac rehabilitation, home monitoring, open-source website, healthcare followup, telemedicine, low-cost

## Abstract

**Background:**

Telemedicine home monitoring of physical rehabilitation in cardiovascular patients, which may substantially improve adherence and, thus, prognosis and quality of life, is an underused practice. Indeed, the Apps and websites available are generic and cannot be easily adapted to each specific rehabilitation protocol. We thus aimed at developing a flexible, low-cost, and open-source telemedicine tool that can be customized and operated by any healthcare professional with just user-level internet knowledge.

**Methods:**

The website was co-designed by an interdisciplinary team, including website developers and clinical experts in physical rehabilitation programs for patients with cardiovascular diseases. The operability and robustness of the website were tested on simulated patients and health professionals, and the suitability of the tutorial for website customization was assessed.

**Results:**

The website asks the patient to complete a periodic diary of physical activities (e.g., intensity, type, duration, warm-up, cool-down, subjective effort). At any time, the patient can see graphs of the different types of exercise performed during a selected period. The website allows healthcare professionals to browse patients’ data, send feedback messages, and export data in a conventional spreadsheet format. The tutorial for website customization was prepared as a learning by doing tool.

**Conclusions:**

The website developed can interest cardiovascular physical rehabilitation professionals aiming at quickly and cheaply setting up an approach for home monitoring programs. This telemedicine tool can also be customized to different clinical applications and is particularly well suited for low-resource settings.

## 1 Introduction

Physical exercise is good for health in general (1) and especially as a tool in rehabilitation programs for patients with different diseases [e.g., cancer (2), respiratory disease (3), and pain (4)]. In patients with atherosclerotic cardiovascular disease and heart failure, exercise training is associated with a reduction in hospitalization, adverse cardiovascular events, and mortality rates (5–7). Therefore, the European and American Guidelines recommend patients with cardiovascular disease enroll in cardiac exercise rehabilitation (6, 7).

Physical rehabilitation programs can be carried out at hospitals or other healthcare facilities. This clinical practice is excellent because healthcare professionals monitor the patient in person. However, the applicability and extension of rehabilitation programs for most patients who could benefit is limited. Indeed, the development of physical rehabilitation programs at healthcare premises is very expensive in terms of material infrastructure and labor requirements. It is also a problem for patients with time/work constraints or not living near such premises thus requiring expensive and bothering patient displacements. This problem is particularly relevant in rural areas with low-density populations and in underfinanced regions such as low- and medium-income countries (8). Fortunately, developing rehabilitation programs at the patient's home overcomes these limitations and has shown effectiveness in a variety of pathologies, particularly in cardiovascular diseases (9). However, to be effective in terms of patient adherence, a home rehabilitation program requires as much frequent follow-up as possible by health care staff. Whereas such follow-up is difficult to apply through in-person home care visits, it is feasible and useful to use telemedicine tools to facilitate patient-professional interaction (8). Unfortunately, the few applications (Apps) and websites available for home monitoring are proprietary and generic and thus cannot be easily adapted to each specific rehabilitation protocol. The only conventional option would require high development costs entrusted to professional website designers. Therefore, to solve this problem, our work focused on designing and testing a website for the home follow-up of physical rehabilitation for patients with cardiovascular diseases. This telemedicine tool should be characterized by its simplicity for patients and staff, safety by ensuring patient anonymity, open-source availability for any user, and negligible exploitation costs. Our specific twofold aims were first to develop a website that any healthcare professional can download and use as it is. A second and more outreaching objective was to provide a user-friendly Tutorial allowing any healthcare professional without training in website design to customize the website content to any specific application field.

## 2 Methods

### 2.1 Website design

**Figure 1:**
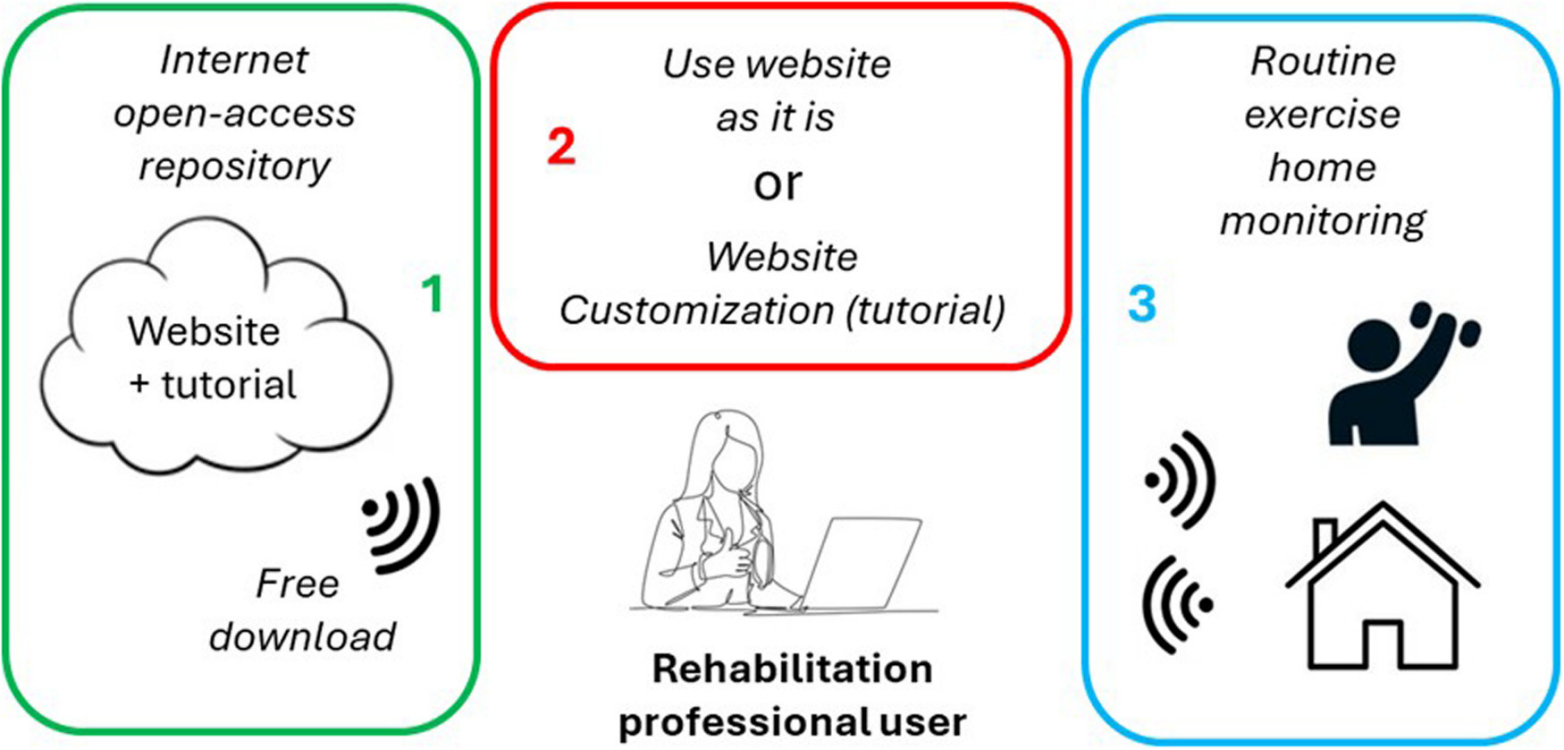
Diagram of the website to follow-up physical rehabilitation of cardiovascular patients at home. Any interested professional can download the website freely (1) and maintain its original format or customize it using the downloaded Tutorial (2). The website is ready for home monitoring patient rehabilitation (3).

We developed a website that can be directly and straightforwardly used by any professional aiming to set up a home cardiovascular rehabilitation follow-up. To this end we followed the innovative approach shown in Figure 1.

The website template, which includes separate areas for patients and healthcare staff, was co-designed by an interdisciplinary team, including website developers and clinical experts in physical rehabilitation programs for patients with cardiovascular diseases. The part of the webpage for the patients is aimed at asking them to complete a periodic diary of different possible physical activities, including the date, type, and time, as well as data on the performance of a warm-up and cool-down and the Borg scale score during exercise (used for subjective self-monitoring through perception of effort). At any time, the patient should be able to see graphs of the data on the different types of exercise performed during a selectable time window. The part of the webpage for healthcare professionals should allow them to browse and select the patients' diaries, observe their data in plot format, and send them a feedback message. The healthcare staff should also be able to export any required patient's data in a conventional spreadsheet format for further analysis.

Once the website prototype was implemented, its usability and reliability were tested by using simulated patients and healthcare staff involving more than 3,000 transferred data and messages. Each simulated patient used the website to introduce the data on their daily rehab exercises, and the testing process was followed up by professionals playing the role of the healthcare staff in charge of the patient's rehabilitation program. At the end of the test, all the professionals involved checked whether there were incidents in the process and verified that all the patient's exercise data downloaded from the webpage for further data analysis were fully accurate and easily usable.

### 2.2 Website customization

To ensure that the website layout can be easily modified for different variants fitting the aims of potential users, we developed a Tutorial for health professionals with no expertise in website development (Supplementary Material). The Tutorial has 3 different sections and is designed as a learning by doing tool (10). The first section is addressed to healthcare professionals who are not familiar with the physical rehabilitation website. This section explains how this website works since it will be used as the original template for creating websites for other applications. The second section of the Tutorial provides step-by-step instructions on how to install the website, create users and verify its functionality. The third section presents two examples: the first one explains how to carry out minor modifications in the webpage template to adapt it to the user specific interest and application; the second example guides healthcare professionals who aim to use the website template to create a home follow-up application for a completely different application in any field of nursing, physiotherapy, and medicine, by using the example of creating a website for general healthcare follow-up.

## 3 Results

### 3.1 Website design

#### 3.1.1 Website platform

The website was created focusing on minimum cost, easy usability, and the possibility to allow straightforward source cloning by any interested user. Among the many different public platforms available for building websites, Webflow™ was chosen to serve as the front-end (the visible part of the website) for its web cloning features. Indeed, this platform offers the possibility to post an entire website, e.g., the one we designed, in their “Made in Webflow” section and to allow other Webflow users to clone this site freely. The Xano™ platform was selected because its 'Snippets' function allows the backend to be cloned easily. After cloning the original site, the user can rename the new website with any specific name (xxx) with the format “https://xxx.webflow.io”. For instance, our web rehabilitation site was named https://exercise-follow-up-template.webflow.io/. If the user wants to replace the extension “.webflow.io” by a more common one (e.g., “.com”), it is possible to buy a website domain (i.e., address) and link it to the generated Webflow site.

The Tutorial in the Supplementary Material shows how any user can freely register on the platforms and clone the website and the backend for their use. Everything required for such cloning can be accessed freely. However, payment is required to achieve the website functionality, specifically to connect the front end to the back end. When following the Tutorial, this payment must be completed before performing any customizations. It involves upgrading the free website plan to a paid plan (see Supplementary Materials).

Following this approach the cost of making the website functional is negligible. Indeed, the only required payment (for connecting the front and back ends) is 168 US$/year as of April 2025, thus less than 0.5 US$/day. In case of optionally buying a conventional extension for the website address (e.g., “.com”) the yearly cost is 10–20 US$ (<0.05 US$/day). Hence, running this website is virtually free.

#### 3.1.2 Patient's interface

**Figure 2:**
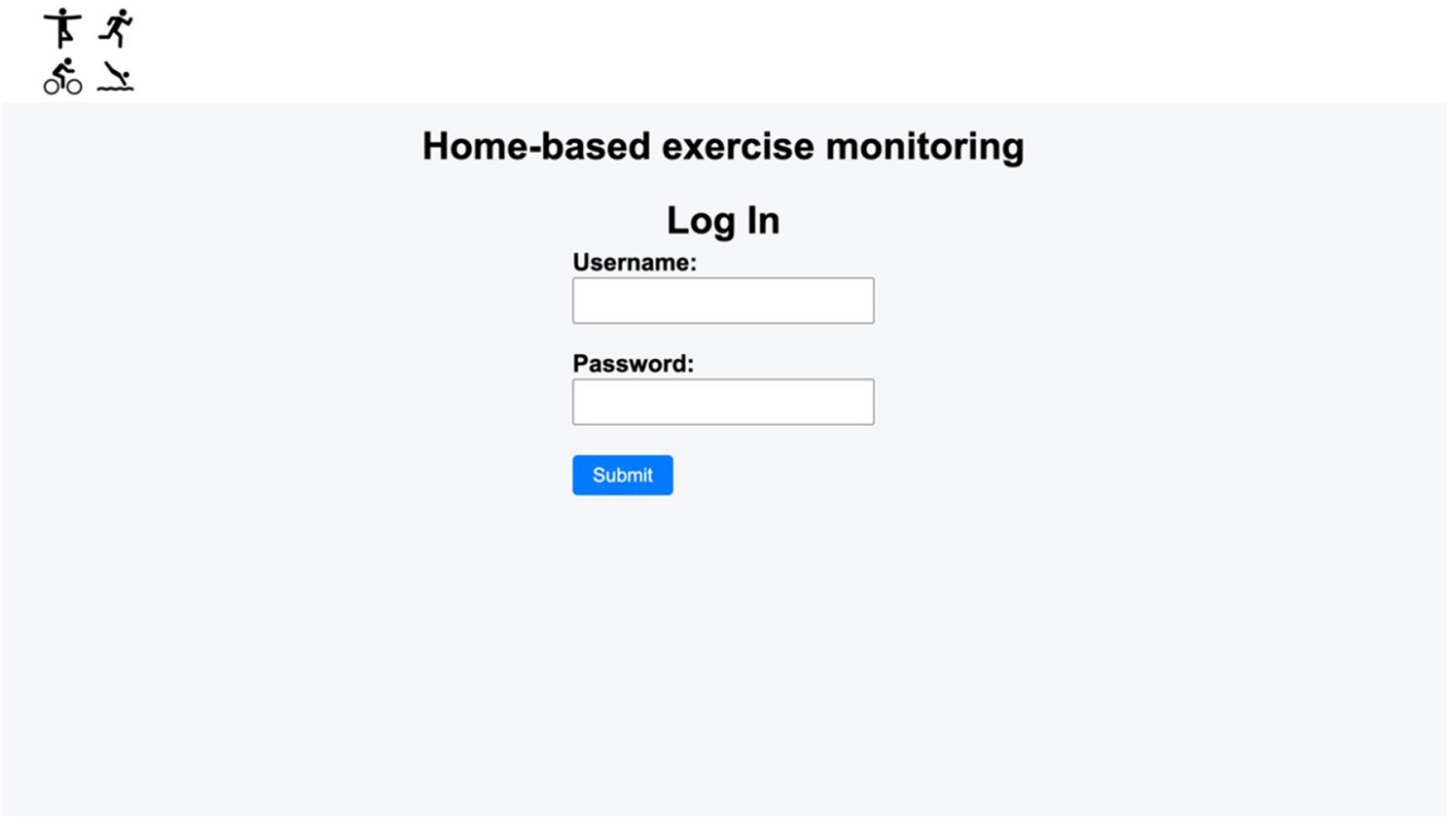
Login page.

When the patient is included in the rehabilitation program website, the healthcare staff in charge provides him/her with a username and a password, e.g., randomly generated, thus ensuring full anonymity on the website and the internet. The entry point of the template website is the login page (Figure 2). Through this page, users are authenticated and then redirected to the patient's interface.

**Figure 3:**
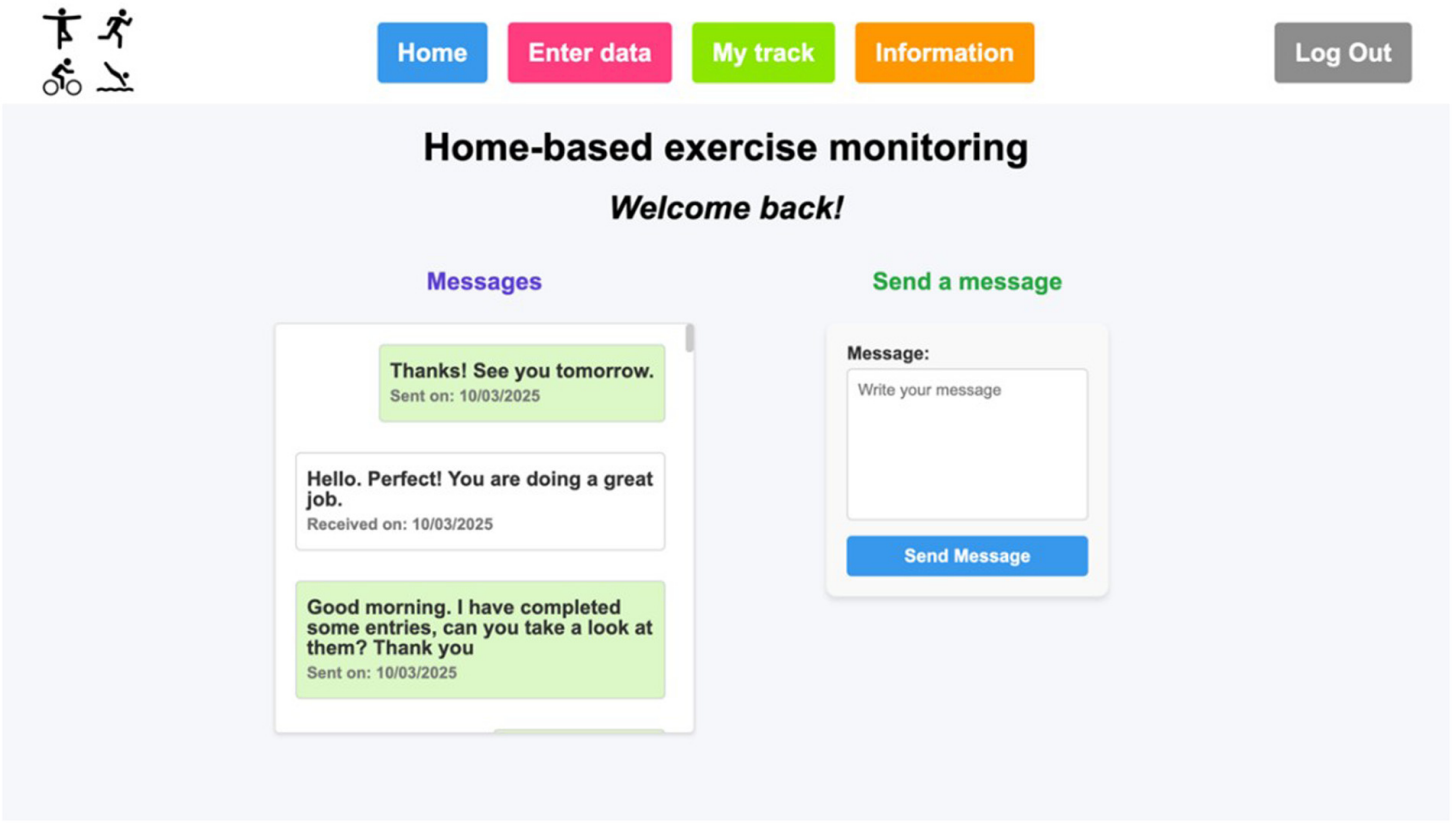
Patient's home page.

**Figure 4:**
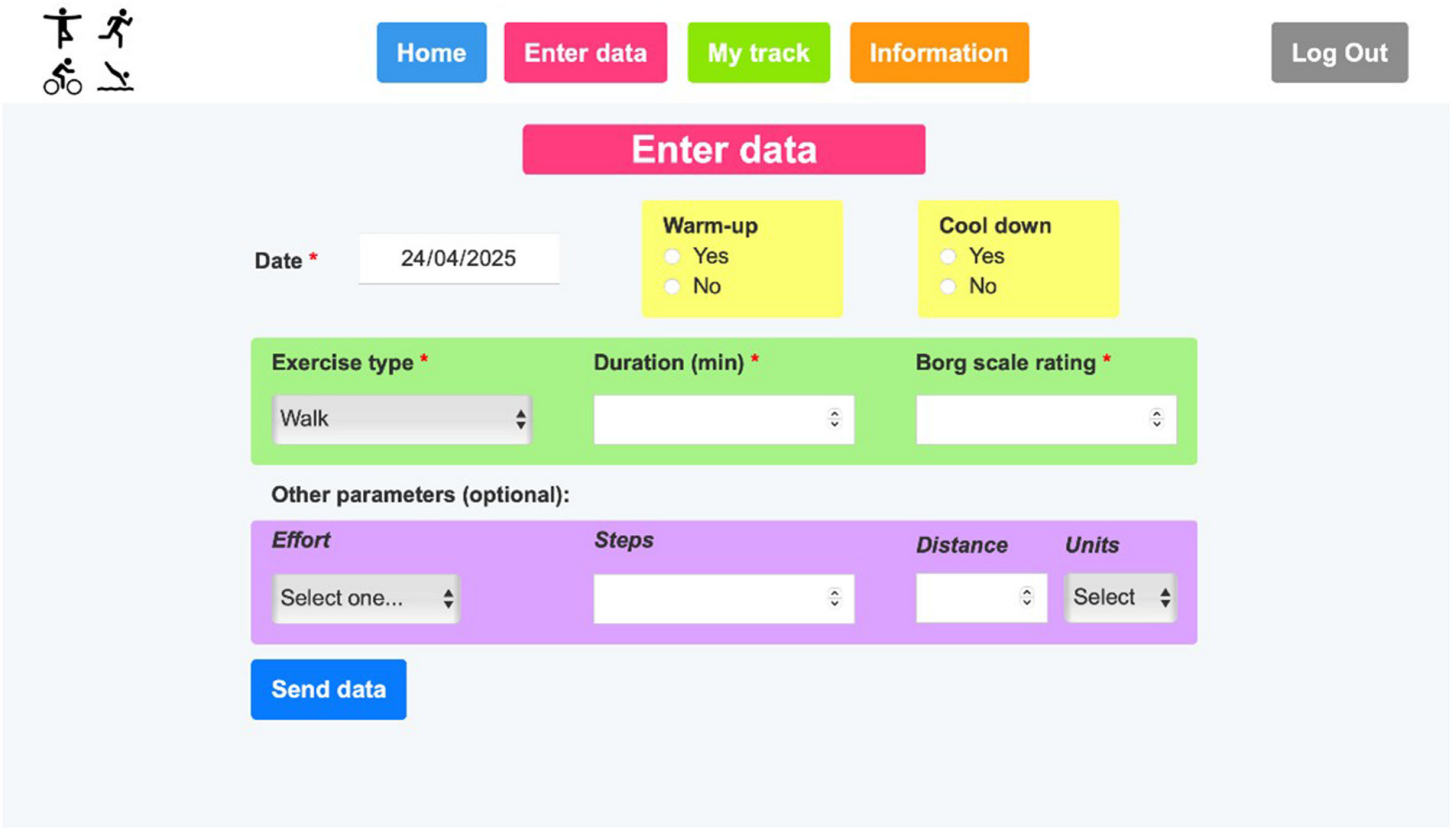
Patient's “Enter data” page.

When a patient logs in, he/she is directed to the patient's home webpage (Figure 3), where the messages interchanged with the healthcare staff can be seen, and the patient can send messages to the health staff. Then, using the menu on the top of the page the patient can go to “Enter data”, “My track” and “Information” pages, as well as log out. At the “Enter data” page (Figure 4), the patient can complete the physical activity diary. The user can select a date, which is useful in case of reporting data of rehabilitation exercises from previous days. The exercise type can also be selected, and, if the patient has done an exercise type that is not included in the list, the “Other” option can be selected, and a free text field will appear to specify it. Additionally, if a patient has exercised more than once a day, more entries can be made for that same day with the details of each physical activity.

**Figure 5:**
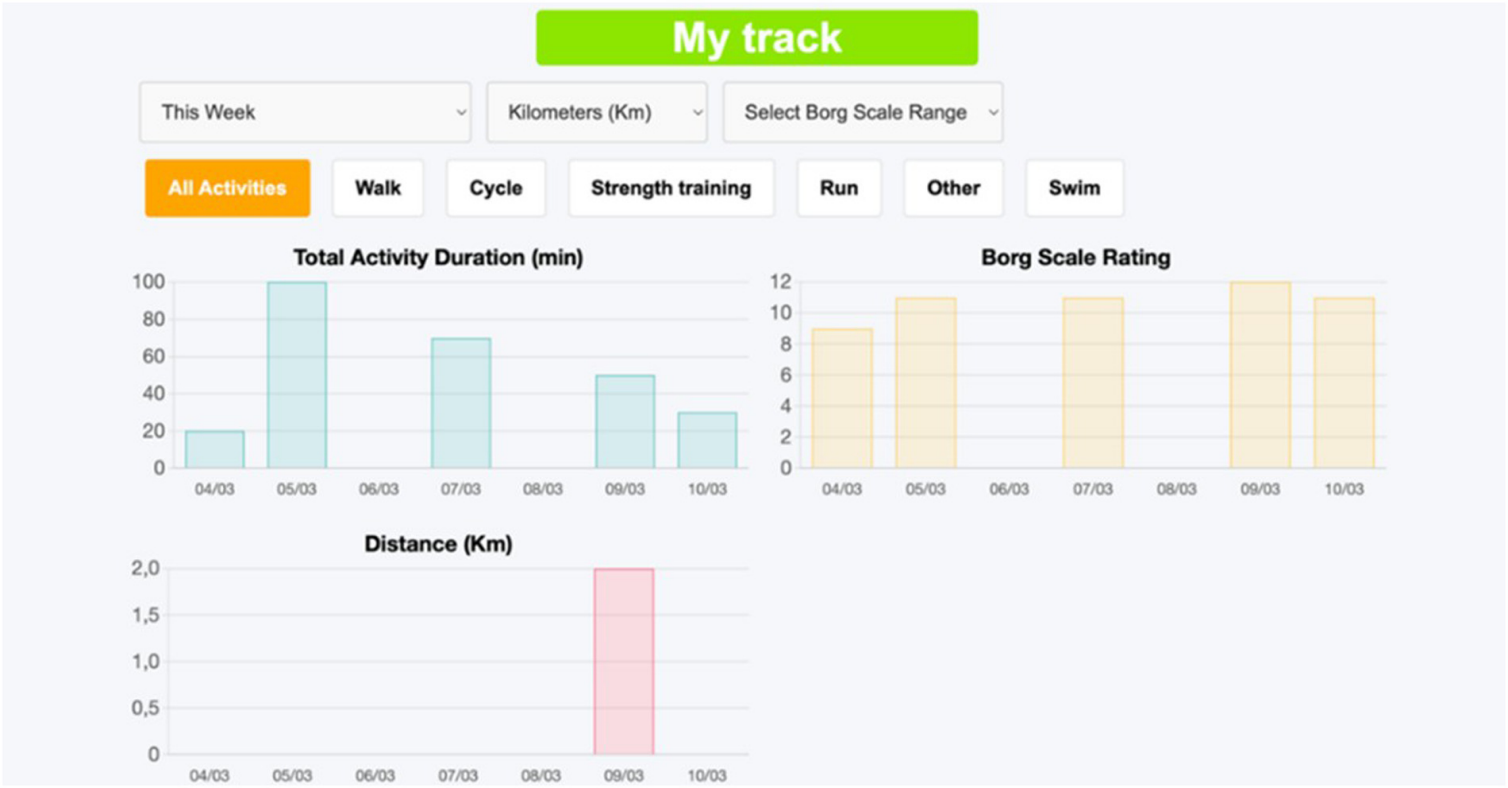
Patient's “My track” page.

**Figure 6:**
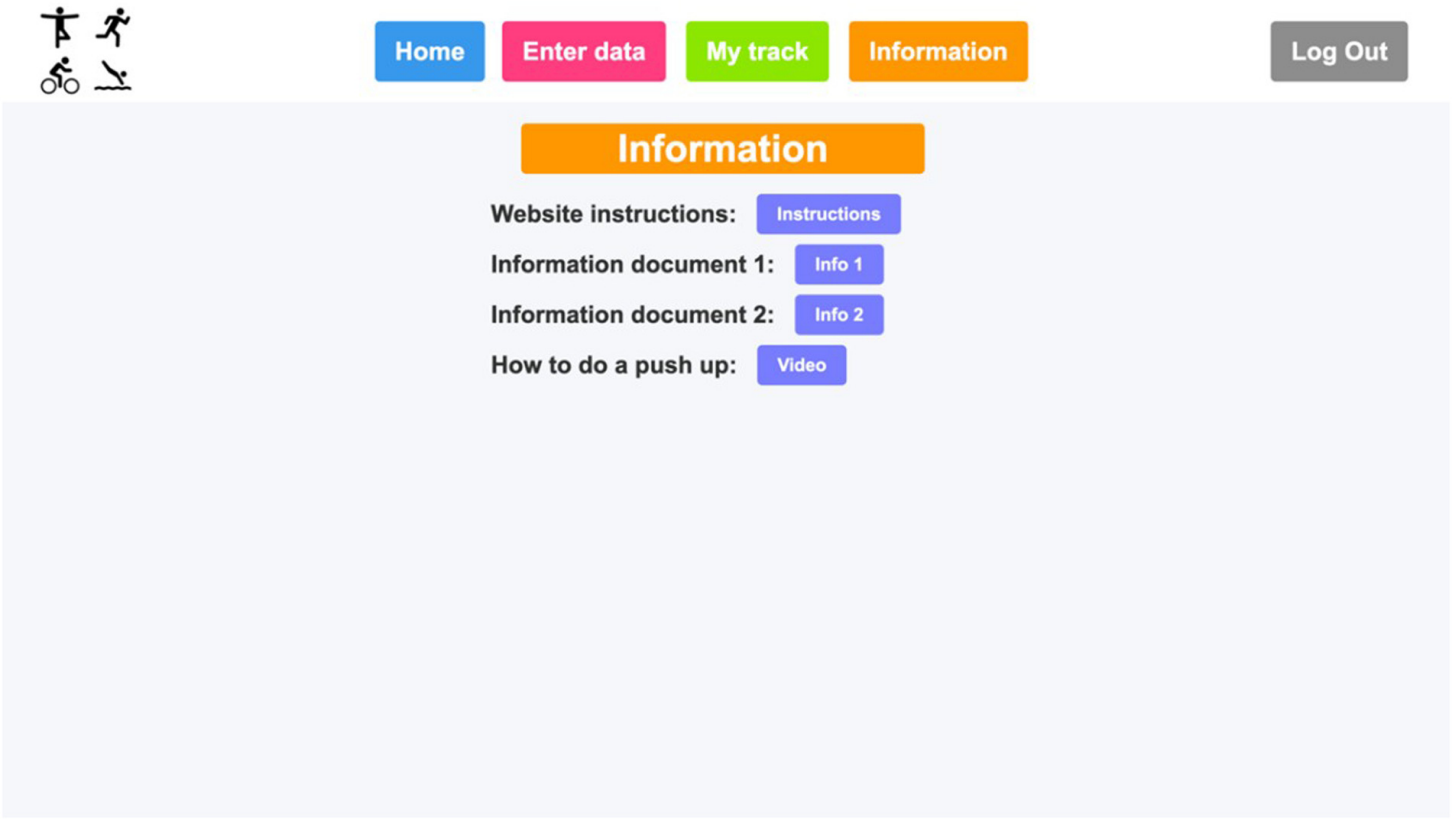
Patient's “Information” page.

By clicking the “My track” button in the top menu, the patient can see the time evolution of the performed exercises (Figure 5). Also, he/she can choose which exercise to see graphically and on which time scale. At the “Information” page (Figure 6), the patient can download the website user instructions and any written or video information that the rehabilitation program has made available to him/her.

By clicking at the “Log Out” button on the top-right of any page, the user will be logged out of the website and redirected to the entry point, i.e., the login page.

#### 3.1.3 Healthcare staff's interface

**Figure 7:**
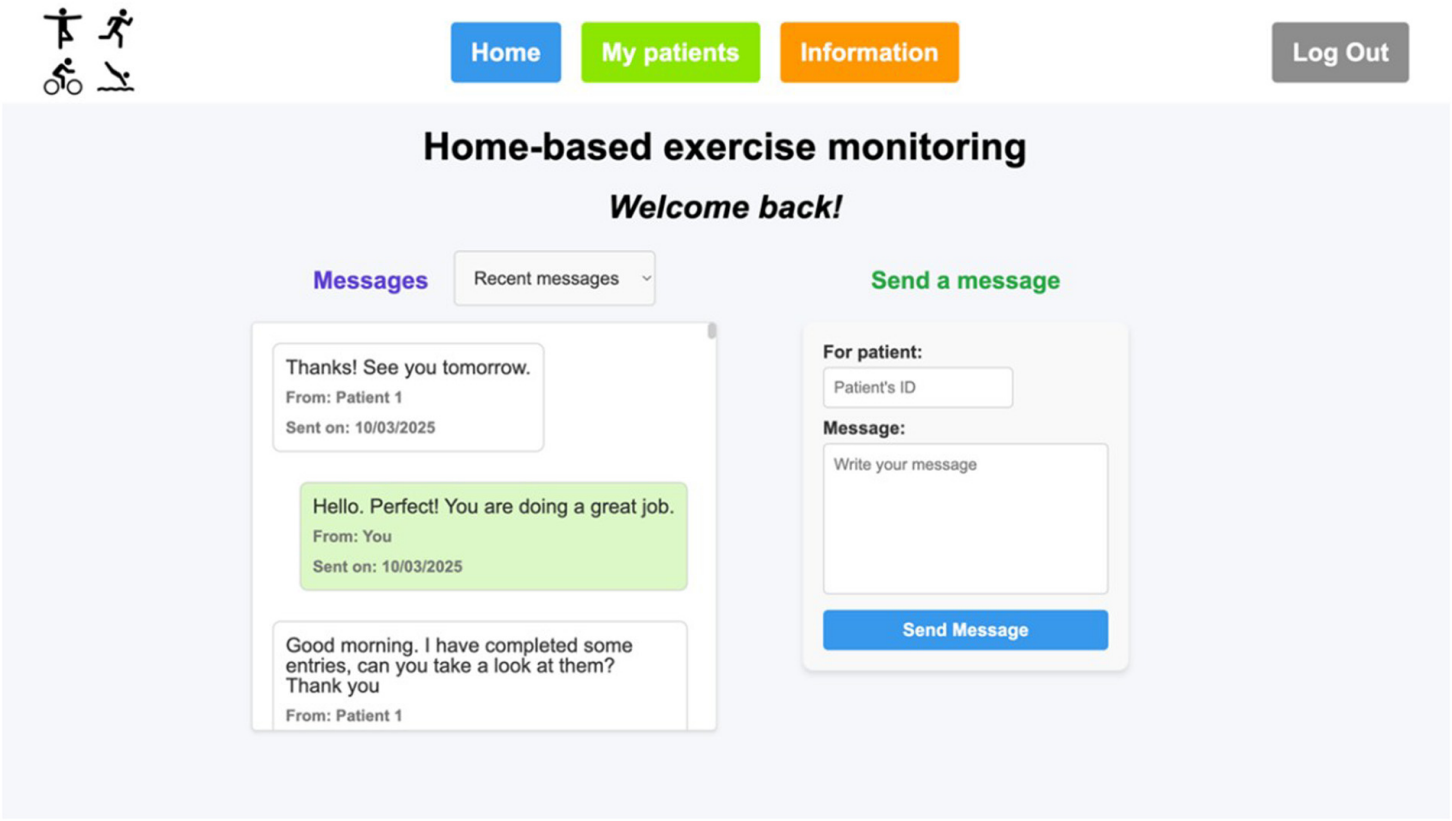
Healthcare staff's home page.

Users who are part of the healthcare staff will be automatically identified as a program professional (according to their username) and directed to the staff's home page (Figure 7). There, the most recent messages interchanged with patients can be seen (either all of them or filtered by patients). Also, messages can be sent by filling the ’Send a message' box with the patient's ID and the message.

**Figure 8:**
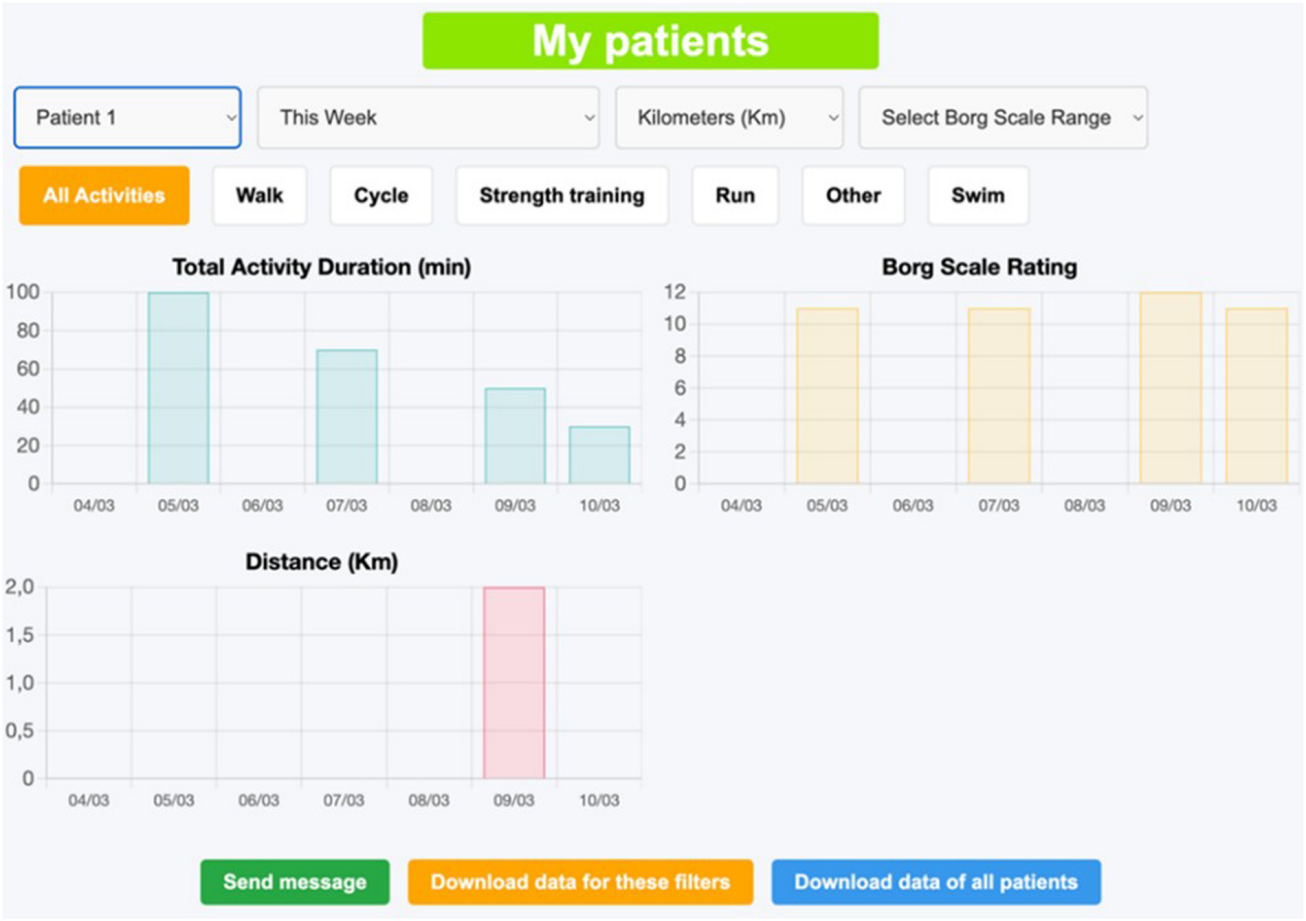
Healthcare staff's “My patients” page.

Through the menu on the top of the page, the staff user can go to “My patients” or “Information” pages, as well as log out. At the “My patients” page (Figure 8), the user can browse and select among all the patients included in the home care program to see a graphical display of the time evolution of the patient's activities. In addition, the buttons below the graphs allow the professional to send a message to the patient (redirecting to the homepage), to download the data (in Excel™ format) corresponding to the graphs on the screen (“Download data for these filters” button), or to download all the data from all the patients (“Download data of all patients” button), so that any analysis tool can be used to further process the data. The Excel™ files data can be accessed by free readers.

The staff interface also has an “Information” page (similar to that of the one in the patient homepage) (Figure 6) where the user can download the website instructions for staff and any other information that has been made available to patients. While being at any page the staff can log out by clicking at the “Log Out” button at the top-right of the page.

The test on the simulated patients and professionals showed that both the patient and healthcare webpages are clear and user friendly, and it was verified that there were no errors in the downloaded data as compared with the ones introduced in the rehabilitation diaries. The designed websites were satisfactorily tested using the most widely used internet browsers (Google Chrome, Safari, Microsoft Edge, Mozilla Firefox, and Opera) (11).

### 3.2 Website customization

**Figure 9:**
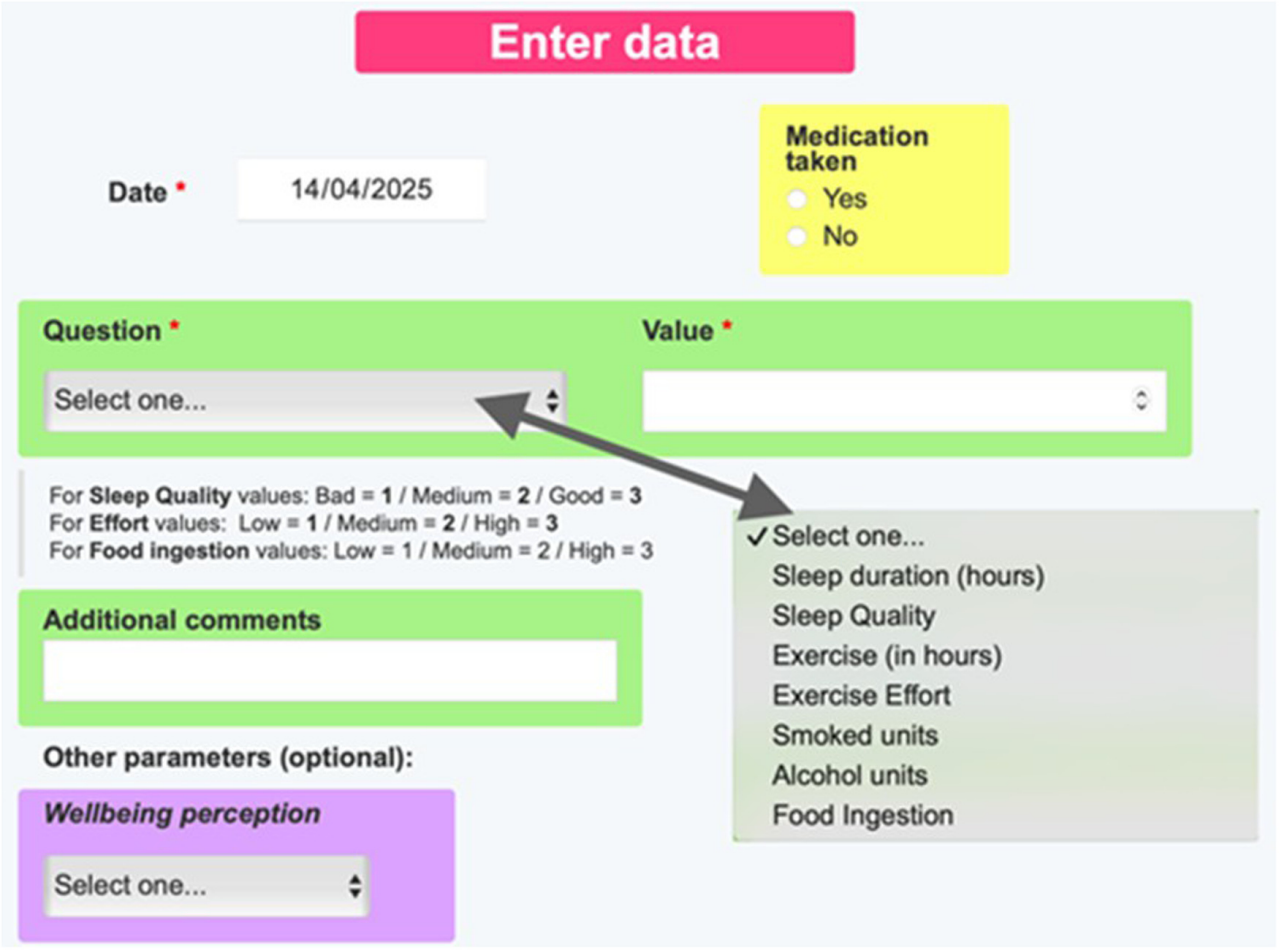
Patient's “Enter data” page in the new customized webpage following the tutorial (supplementary Materials).

**Figure 10:**
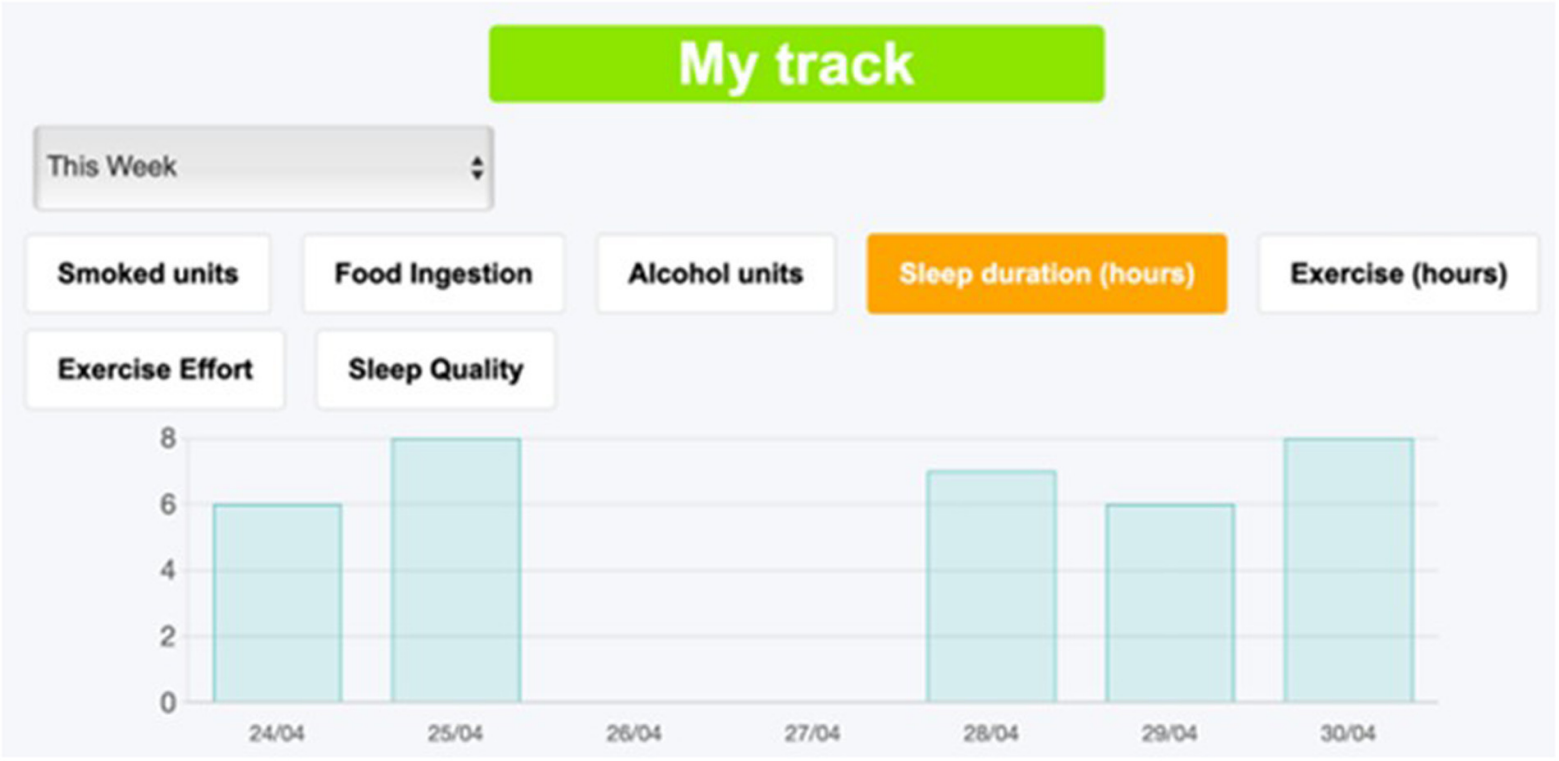
Patient's “My track” page in the new customized webpage following the tutorial (supplementary Materials).

The previously shown website for physical rehabilitation can be used as a template for slightly modifying it or for creating a different website for other applications in homecare follow-up, as explained in detail in the Tutorial (Supplementary Materials). The second example of the Tutorial's third section focuses on the steps to follow for modifying the questions to ask the patient and for redesigning the webpage appearance. Given that the Tutorial is designed as a learning by doing tool for healthcare users with no previous training in website construction, the reader is asked to create a new website example aimed at following up issues regarding the general health status of the patient. This example is not comprehensive and thus not aimed at immediate use, it is simply intended for illustrating the process for website customization. When the reader fulfils the Tutorial tasks, he/she is able to create a website for following-up patient objective behaviors (medication compliance, sleep time, exercise duration, smoking, alcohol drinking, and eating) and their subjective perceptions (sleep quality, exercise effort and general wellbeing), as illustrated in Figure 9. The “My track” page then shows the time course of the new variables (Figure 10). The Tutorial also describes how to modify other webpage details, e.g., to change webpage logo and presentation or modify the files in the “Information” pages.

The estimated time required to perform the different tasks described in the Tutorial by a person who has a user-level knowledge of internet, not trained in webpage construction, were the following: (1) 1.5–1.75 h to clone and install the website as it is, (2) 0.5–0.75 h to implement a simple variant of the downloaded website, and (3) 1.25–1.5 h to customize it to for a different application in another healthcare application.

## 4 Discussion

The telemedicine tool we present in this work enables any professional in the field of physical rehabilitation of patients with cardiovascular diseases to immediately and straightforwardly initiate a home care monitoring program by directly using the developed website. Moreover, the Tutorial we propose may allow that, after a minimum training, any healthcare professional without previous experience in website design is able to customize the patient's and healthcare professional's pages for home monitoring in any clinical specialty beyond physical rehabilitation.

We based our telemedicine tool on public internet platforms to avoid requiring the use of proprietary information and communication networks (e.g., from hospitals or other institutions). This enables any healthcare professional to use the website in regions with poorly available Information and Communication Technology provisions from specific institutions, as is common in small or rural healthcare centers or in private practices. The two commercial internet platforms we selected provide the highest data security, processing integrity, privacy, and confidentiality according to most rigorous safety standards (SOC 2 Type 2 certification). Moreover, it is noteworthy that the website was designed to ensure full patient's privacy protection. Indeed, on the one hand, no personal or clinical data related to the patient's health is introduced into the website (only anonymous data on daily exercise). Furthermore, the patient's username and password can be randomly generated by the healthcare professional, hence including no information relative to the patient. Interestingly, the code file linking each patient with their username and password can be kept in a private file curated by the healthcare professional and stored in a physical repository outside the internet and even outside any computer. Therefore, using safe internet platforms and the fact that no patient's personal identification data are introduced into the website offer the highest possible privacy guaranties.

The webpages we designed were deliberately simple to facilitate usability for most patients and healthcare professionals. For instance, each tab in the patient page is contained in a single screen, the labels and windows to introduce data are great enough to enhance visibility for aged patients or those with visual limitations, and colors belong to a blind color friendly palette (12). For the sake of simplicity and flexibility, the data processing and viewing in the healthcare professional page was minimized. Alternatively, allowing the data to be downloaded into a conventional data sheet format makes it possible that any healthcare user involved in the program to further define a specific graphical and statistical processing format. Also, for simplicity we avoided including video calls, with the bidirectional patient-healthcare professional communication based on written messages into the website. If deemed necessary, nowadays a video call can always be established by widespread conventional tools. Interestingly, the website is designed for friendly appearance in both computer, tablet or mobile phone screens. The latter can be particularly useful for open-source initiatives (13) for home monitoring in low- and middle-income countries, where mobile phones are progressively ubiquitous (14).

A remarkable feature of our approach in developing this telemedicine tool was its flexibility to be easily customized for a wide range of applications in home monitoring of patients with different health diseases. As illustrated by the customization section in the Tutorial (a simple possible example for general healthcare follow-up), the website questions and pages layout can be easily modified to cover almost any question that any home came program may need, e.g., on quality of life, patient activities, amount of food ingestion, and physiological variables from home sensors such as hearth rate, blood pressure, weight, glycemia and oxygen saturation. Accordingly, the website could be easily adapted for patient home monitoring in endocrinology, pneumology, sleep disturbances, neurology, psychiatry, postoperative and elder patient care, high-risk pregnancies monitoring, or neonatal follow-up (15–18). It could be also possible to customize the website for professional team-building purposes or for improving coordination among healthcare staff in places without the availability of professional communication networks (19–21). For instance, following the rationale and structure of the website template we present herein, the role of the patients can be replaced by health community workers or small rural/local centers, and reference hospitals can play the role of the health professionals as network coordinators. Accordingly, instead regarding exercise activity, the data interchanged among them could be on a variety of aspects related to clinical practice (e.g., diseases incidence, treatments application, waiting lists or professional inter-consultation), thus allowing to set up an empowering customized network to improve patient's healthcare. Most importantly, with the aid of the Tutorial, such website customization can be carried out by health professionals who are not experts in the construction of webpages, and simply requires internet access, with no need for institutional digital networks availability.

This Methods report describes the design, technical robustness and feasibility of a novel telemedicine approach developed by an interdisciplinary team including health care professionals routinely involved in rehabilitation programs. However, future patient studies should substantiate its performance and outreach in clinical routine.

## 5 Conclusions

First, the website developed and presented herein can be of interest for cardiovascular physical rehabilitation professionals aiming at quickly setting up a free tool for home monitoring programs. Second, this telemedicine tool can be customized for different fields of health care applications. Finally, being open-source, low-cost, and not requiring institutional digital infrastructure, this approach can be of particular interest in low-resource settings.

## Data Availability

The original contributions presented in the study are included in the article/Supplementary Material, further inquiries can be directed to the corresponding author.
