# Supplementary material for: Open-source customizable website to follow-up physical rehabilitation of cardiovascular patients at home
Source: Front Cardiovasc Med. 2025 Jul 25;12:1633106. doi: 10.3389/fcvm.2025.1633106 (PMC12331590; doi:10.3389/fcvm.2025.1633106)
Supplement: Supplementary file 1 [file Datasheet1.pdf]

SUPPLEMENTARY MATERIAL

for

Open-source customizable website to follow-up physical rehabilitation of cardiovascular patients at home

A. Sabala, D. Mcbrearty, R. Salama, R. Farré, A. Loughnane, J. Otero, N. Farré

TUTORIAL

Index

Website Template Description *4*

1. **Patient's interface** **4**
2. **Healthcare staff's interface** **6**

Website Template Implementation (1h 30min – 1h 45min) *9*

1. **Getting Started** **9**
    1.1. Creating a Webflow account 10
    1.2. Creating a Xano account 12
2. **Installing the Website** **15**
    2.1. Cloning the Website – Webflow 15
    2.2. Cloning the Backend – Xano 16
    2.3. Connecting the Website and Backend (Webflow + Xano) 19
3. **Setting Up the Website's Address** **27**
4. **Publishing the Website** **29**
5. **Creating User Profiles** **31**
6. **Verifying Website Functionality** **34**
    6.1. Verifying Login Functionality 34
    6.2. Verifying Messaging Functionality 35
    6.3. Verifying Data Submission and Visualization 38
7. **Security Best Practices** **41**

Website Template Customization *43*

Example 1: Adapting the Physical Rehabilitation Monitoring Template *(30min – 45min)* **44**

Example 2: Modifying the Website for Patient Behaviour and Wellbeing Monitoring *(1h 15min – 1h 30min)* **46**

Annex A: How to Modify the Website *68*

1. **Changing the website's logo (level of difficulty: 1/5)** **68**
2. **Modifying the 'Information' pages (level of difficulty: 1/5)** **71**
    2.1. How to add a document, image, link or video 71
    2.2. How to delete a document, image, link or video 75
3. **Modifying the message system (level of difficulty: 2/5)** **77**
    3.1. Restrict patients from sending messages 77
    3.2. Remove the messaging system 79
4. **Modifying the form (level of difficulty: 4/5)** **81**
    4.1. Explaining the form's behaviour 81
    4.2. Managing Required fields 83
    4.3. Managing the Exercise Type Field 87
    4.4. Changing the remaining fields 93
    4.5. Deleting a field 100
5. **Modifying the graphs (level of difficulty: 2/5)** **102**
    5.1. Changing the title of a graph 102
    5.2. Deleting a graph 103
    5.3. Deleting a filter 106
6. **Deleting a User's Data (level of difficulty: 2/5)** **108**

This manual provides a description of the website and step-by-step instructions on how implement it as it is or customize it for a completely different application in any field of nursing, physiotherapy and medicine.

As seen in the index, the manual is divided into three parts:

- **Website Template Description** – Learn how the website works.

- **Website Template Implementation** – Follow the steps to set up and test the website.

- **Website Template Customization** – Contains two examples of how to adapt the website to different needs. Choose the example that best suits your needs.

Website Template Description

The entry point of the template website is the login page (Figure 1). Through this page, users are authenticated and then redirected to the patient's interface, if they are patients, or to the healthcare professionals' interface, if they are staff members.

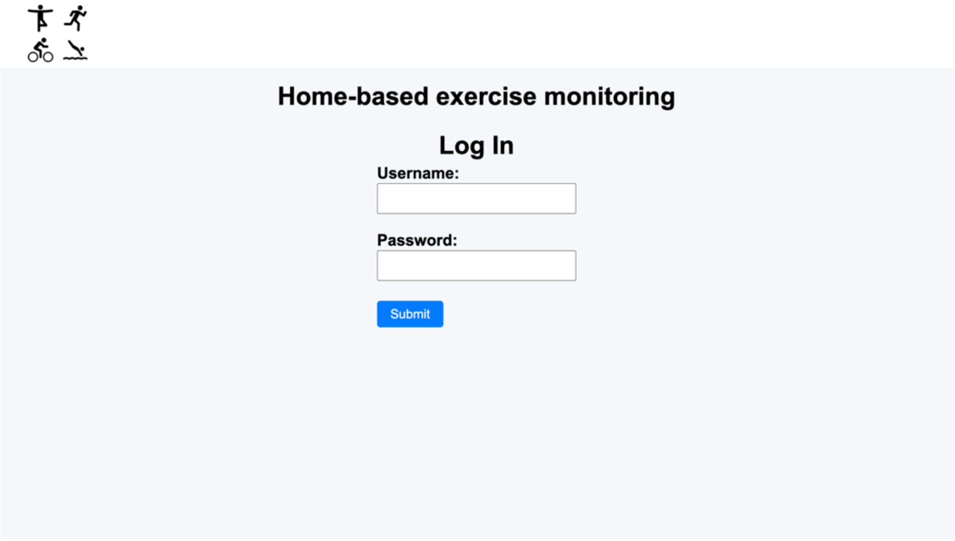

Figure 1. Login page

1. Patient's interface

When a patient logs in, they are directed to the patient's home webpage (Figure 2), where the messages interchanged with the healthcare staff can be seen, and the patient can send messages to the healthcare staff.

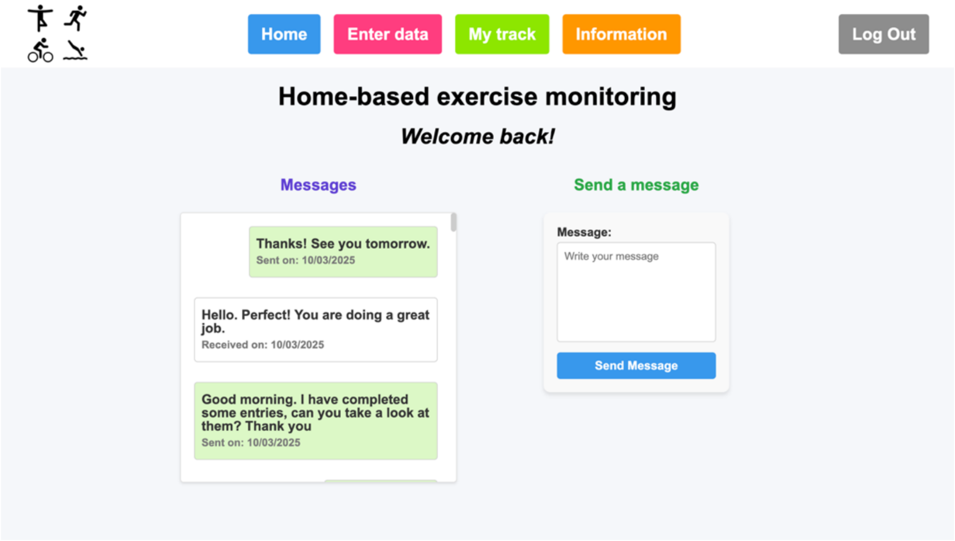

Figure 2. Patient's home page

Then, using the menu on the top of the page the patient can go to 'Enter data', 'My track' and 'Information' pages, as well as log out. At the 'Enter data' page (Figure 3), the patient can complete the physical activity diary. The user can select a date, which is useful in

case of reporting data of rehabilitation exercises from previous days. The exercise type can also be selected, and, if the patient has done an exercise type that is not included in the list, the 'Other' option can be selected and a free text field will appear to specify it. Additionally, if a patient has exercised more than once one day, more entries can be made for that same day with the details of each physical activity.

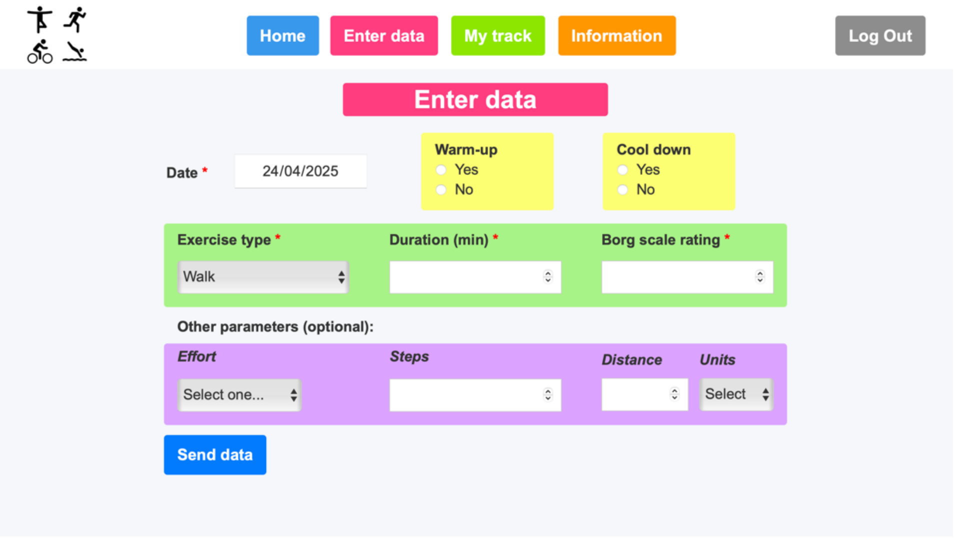

Figure 3. Patient's 'Enter data' page

By clicking the 'My track' button in the top menu, the patient can see the time evolution of their exercises (Figure 4). Also, users can choose which exercise to see graphically and on which time scale.

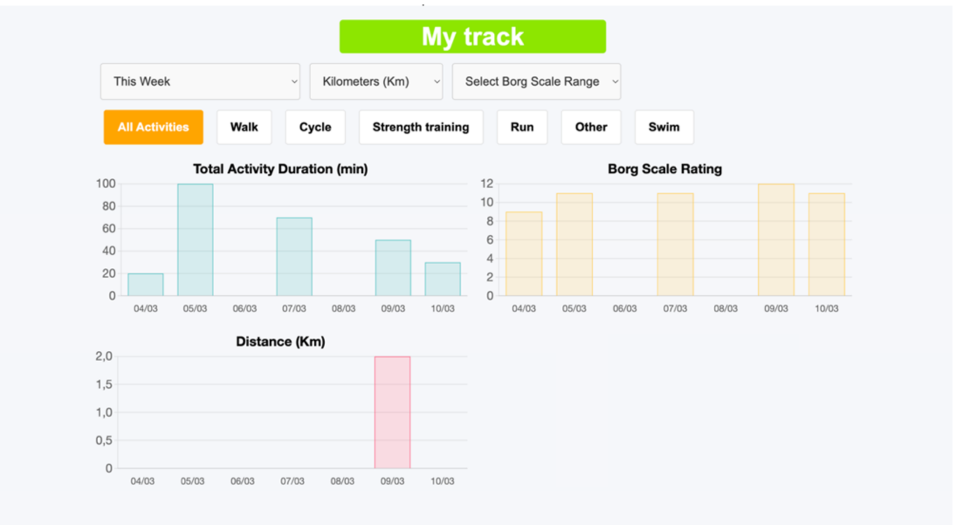

Figure 4. Patient's 'My track' page

Then, at the 'Information' page (Figure 5), the patient can download the website user instructions and any written or video information that the rehabilitation program has made available to them.

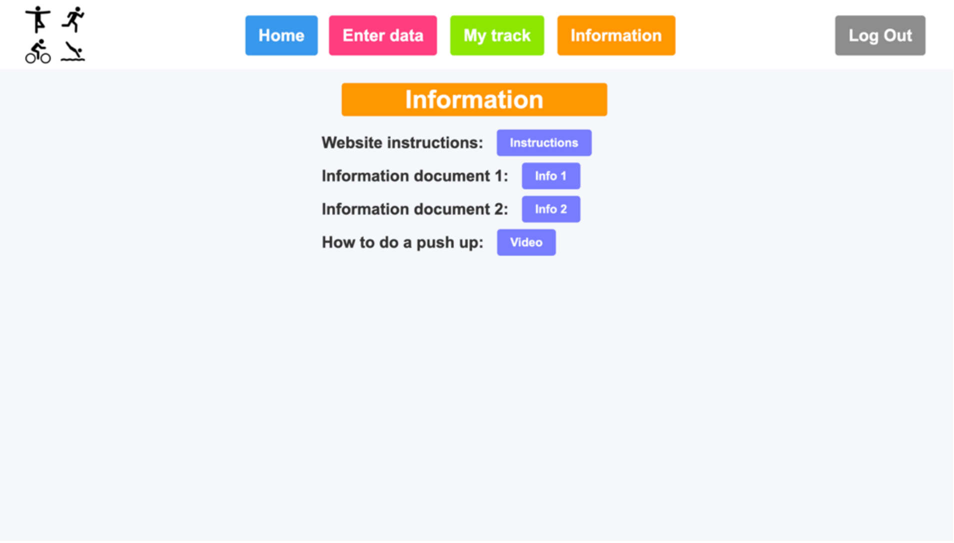

Figure 5. Patient's 'Information' page

By clicking at the 'Log Out' button on the top-right of any page, the user will be logged out of the website and redirected to the entry point, the login page.

2. Healthcare staff's interface

Users who are part of the healthcare staff, will be directed to the staff's home page (Figure 6). There, the most recent messages interchanged with patients can be seen (either all of them or filtered by patient). Also, messages can be sent by filling the 'Send a message' box with the patient's ID and the message.

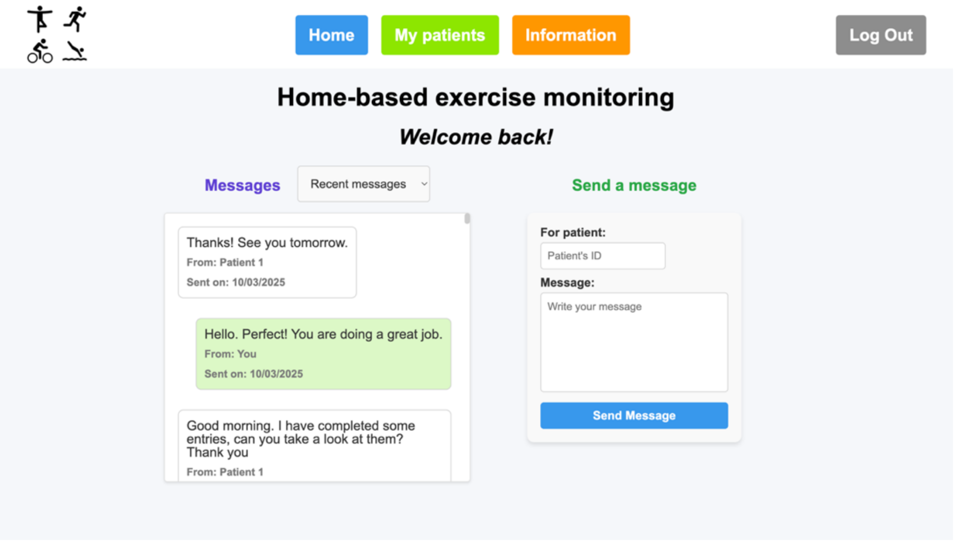

Figure 6. Healthcare staff's home page

Through the menu on the top of the page, the staff user can go to 'My patients' or 'Information' pages, as well as log out.

At the 'My patients' page (Figure 7), the user can browse and select among all the patients included in the home care program to see a graphical display of the time evolution of the patient's activities. In addition, the buttons below the graphs allow the

professional to send a message to the patient (redirecting to the 'Home' page), to download the data corresponding to the graphs in an Excel format ('Download data for these filters' button), or to download all the data from all the patient ('Download data of all patients' button), so that any analysis tool can be used to further process the data.

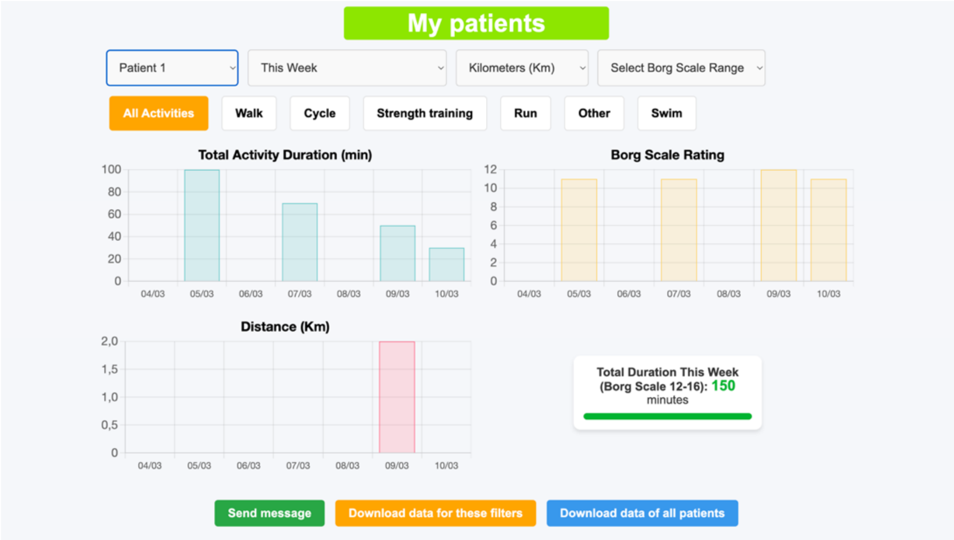

Figure 7. Healthcare staff's 'My patients' page

Lastly, the staff interface also has an 'Information' page (Figure 8) where the user can download the website instructions for staff and any other information that has been made available. Also, at any page the staff can log out by clicking at the 'Log Out' button at the top-right of the page.

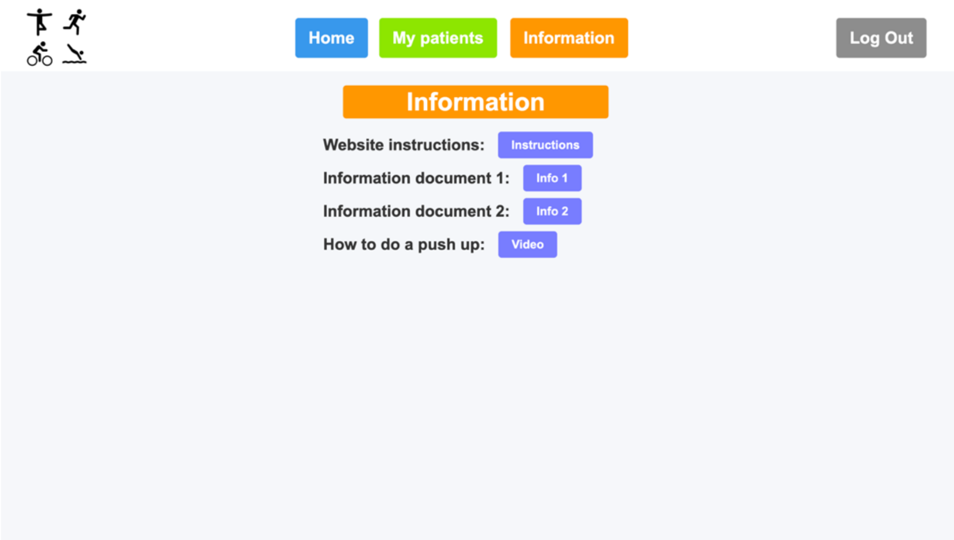

Figure 8. Healthcare staff's 'Information' page

Website Template Implementation *(1h 30min – 1h 45min)*

1. Getting Started

Before starting the installation process, you will need to create an account in both **Webflow** and **Xano**.

- **Webflow** is the platform that will be used to design and publish the frontend of the website (the part that users of the website see).
- **Xano** is the platform that will act as the backend (where the website's data is stored and managed).

Throughout this manual, we will use **Webflow** to modify the visual elements of the website. Each time we make changes, we will publish them so they appear in the live site. **Xano** will be used less frequently, mainly for connecting it with Webflow and for occasional modifications.

In this section, you will find the instructions on how to create both a Webflow and a Xano account. These accounts will later be used to copy (or 'clone') the frontend and the backend of the website.

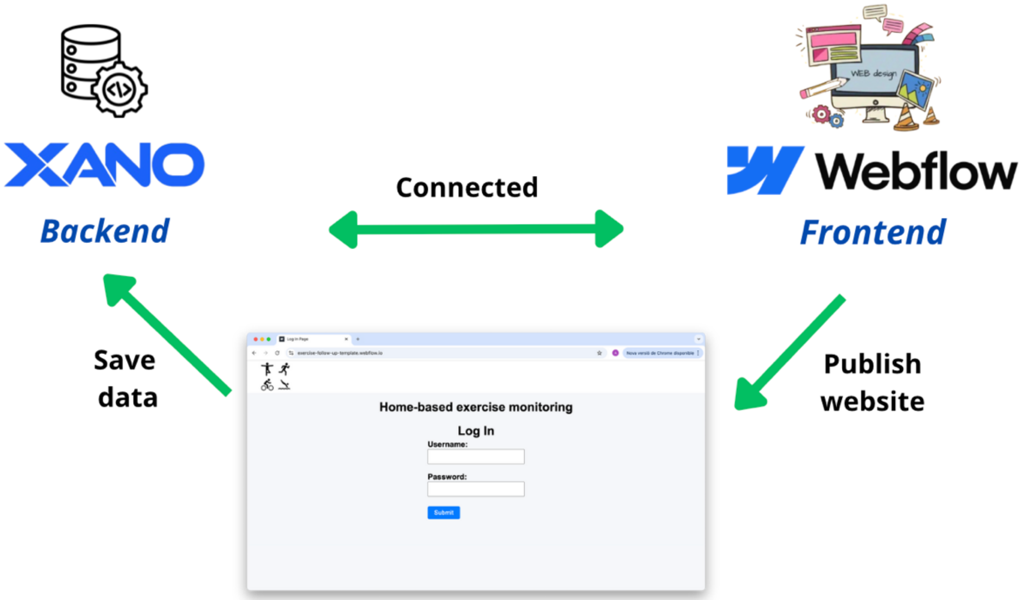

Figure 9. Diagram of the platforms used for the website implementation

1.1. Creating a Webflow account

Note: The goal of this section is to create a Webflow account. *If any questions or screens appear differently than described, do not worry. The goal is to successfully create your Webflow account* ***and*** *ensure that you* ***verify it****.*

1. Go to Webflow's website.

2. Click the **'Get started – it's free'** button at the top-right of the page (Figure 10)

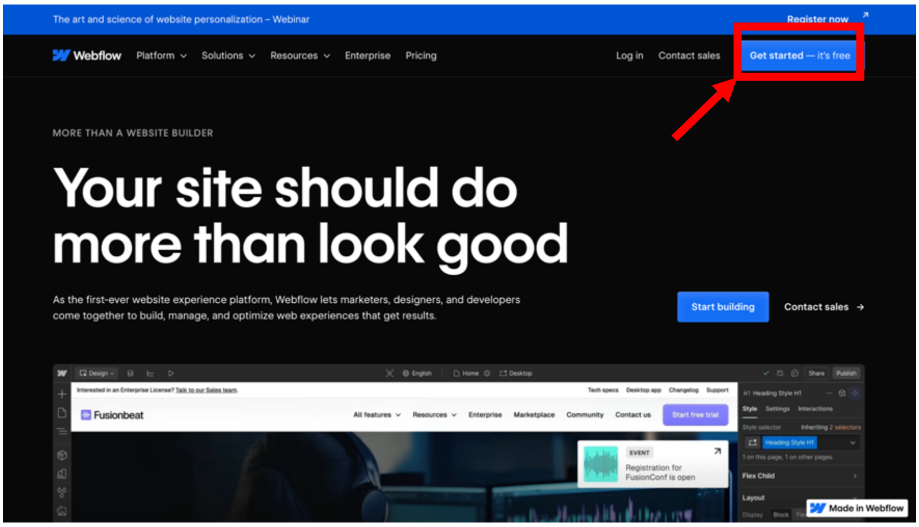

Figure 10. Webflow's home page

3. In the registration form (Figure 11):

- Enter an email and click ***Continue***.
- Create a password that meets the requirements.

(If preferred, you can also sign in with Google. If you choose this option, you will not be asked to verify your email and can skip step 4.)

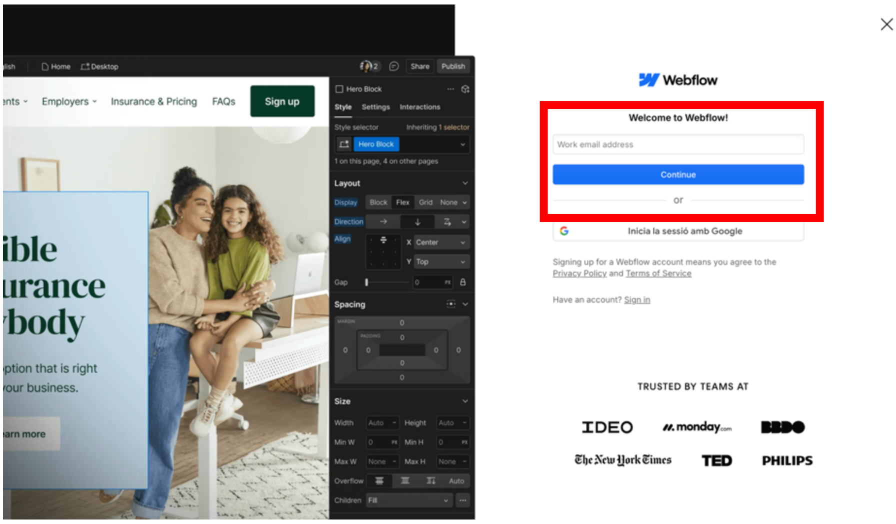

Figure 11. Webflow's registration form page

4. **Go to your email** and **click at the link** sent to you to **verify your account**. This is a very important step.

5. Return to the Webflow page you were before. Fill the questions and click *Continue* (Figure 12).
For more details, refer to steps 5.1-5.3.

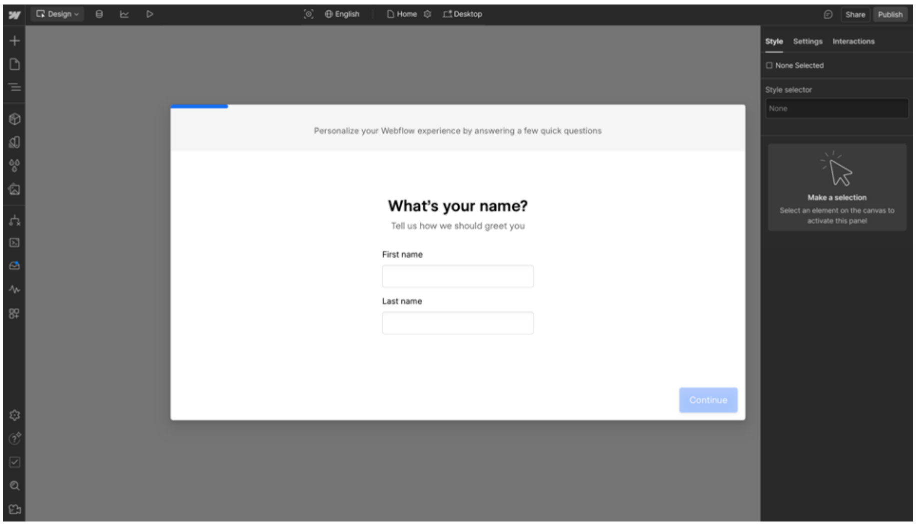

Figure 12. Webflow's initial questions

5.1. Answer the following questions however you prefer:
- 'What's your name?'
- 'What do you want to build websites for?'
- 'Are you a student?'
- 'What type of site are you looking to build today?'

This will not affect the site we create later.

5.2. When asked:
- 'Are you interested in hiring someone to help build your site today?'

Select ***No***.

5.3. To the question:
- **'Select a way to get started'**

Choose ***Blank site***.
A default site name will be generated, leave it as it is and click ***Create site***. This name does not matter, as we will not use this website later.

6. Wait for the default website to be loaded.
    - Then, hover your mouse over the **'W' logo** in the top-left corner of the screen.
    - A **menu icon (three horizontal lines)** will appear (Step 1- Figure 13), **click it**.
    - Select ***Dashboard*** (Step 2 - Figure 13).
    - This will take you to your Webflow Workspace, where the default blank site we just created will appear. You can delete this blank site, as we will not use it.

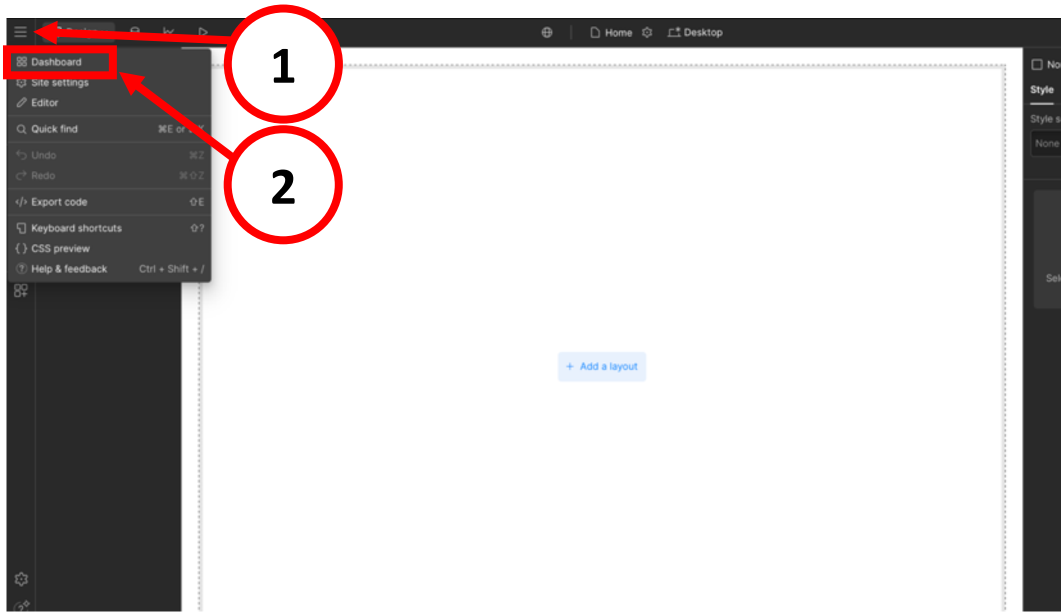

Figure 13. Webflow's default blank site

7. **Congrats!** You have successfully created your Webflow account.

1.2. Creating a Xano account

Note: The goal of this section is to <u>create a Xano account</u>. *If any questions or screens appear differently than described, do not worry, continue with the process. The goal is to create an account.*

1. Go to Xano's website: https://www.xano.com/. **Use Google Chrome**.

2. Click the **'Get started for free'** button at the top-right of the page (Figure 14).

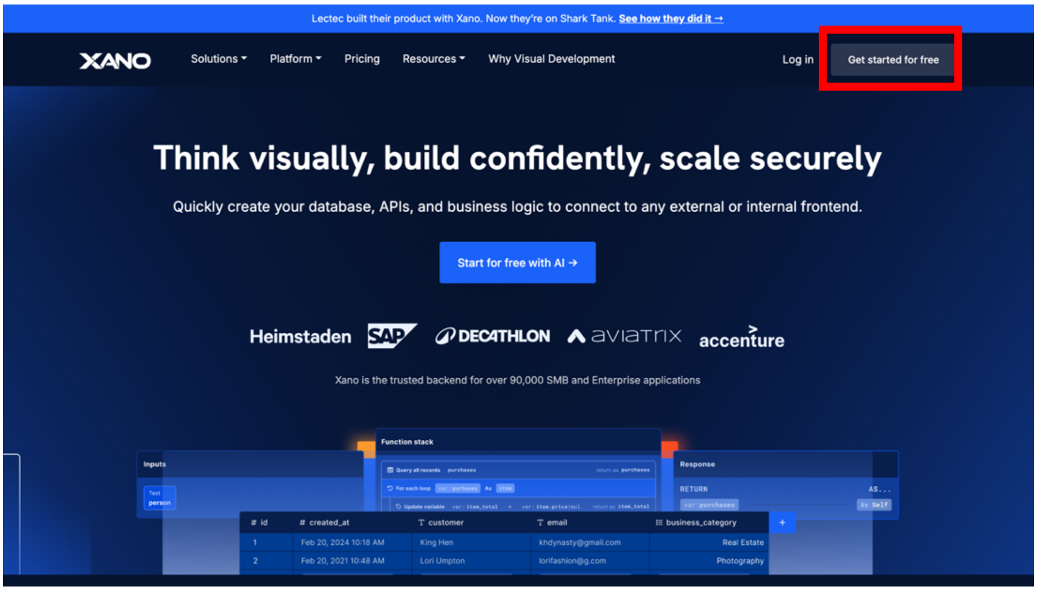

Figure 14. Xano's home page

3. In the registration form (Figure 15):

- Fill out the details
- Click ***Sign up***

(If preferred, you can also sign up with Google or Github to create your account)

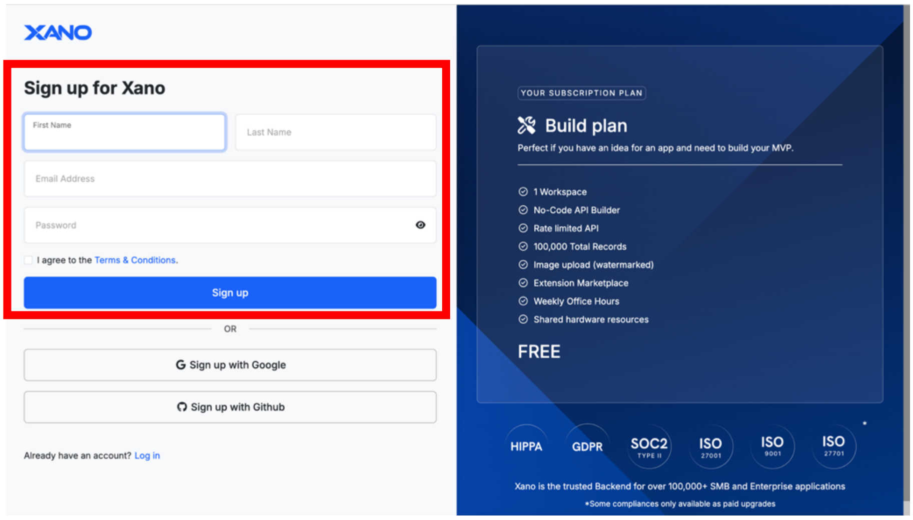

Figure 15. Xano's registration form page

4. Complete the set-up questions and click *Continue* (Figure 16).
   Refer to steps 4.1-4.4 for details.

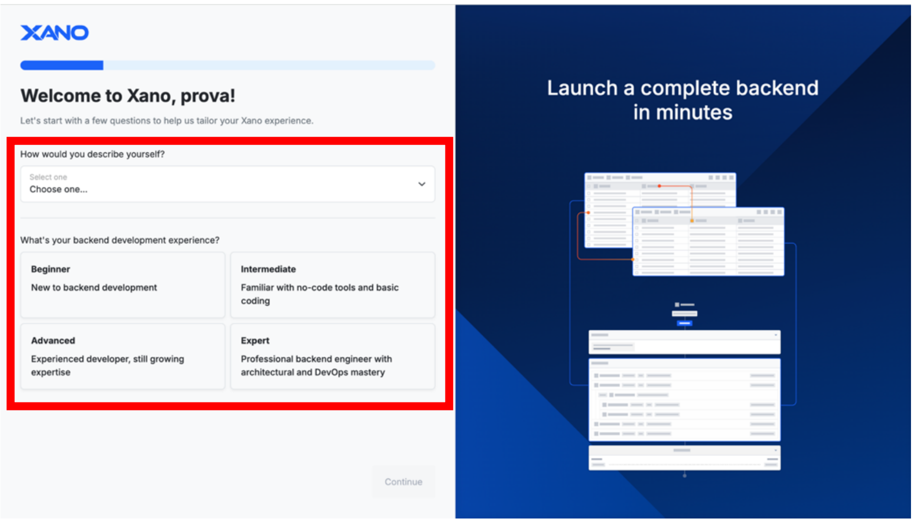

Figure 16. Xano's set-up questions

Note: If you see slightly different or additional questions, don't worry. Answer them, this won't affect the creation of our site.

4.1. Answer the following questions however you prefer:
- 'Where do you work?'
- 'What's your backend development experience?'

Your answers will not affect the process.

4.2. To the question:
- 'What would you like to do?'

Feel free to choose any option. For example, you can select ***Build an app***.

4.3. When asked:
- 'How would you like to get started?'

Answer ***Start from scratch***.

4.4. On the **'Finalize your Workspace'** page:
- Enter a name for your workspace. For example, **'Website'**.
- To the questions:
  - 'What tool or front-end will Xano be connected to?'
    Select ***Front-end***.
  - 'What front-end do you plan to use?'
    Select ***Webflow***.
- **Uncheck** the option **'Set up authentication for me'**.
- Click ***Create workspace***.

5. **Congrats!** You have successfully created your Xano account, where data will be stored.

2. Installing the Website

In this section, we will:

- **Clone the website in Webflow** – We will copy the visible part of the website.
- **Clone the backend in Xano** – This will contain the structure the website needs to function and store its data.
- **Connect the website and the backend (Webflow + Xano)** – We will link the website with its backend structure, in order to work properly.

2.1. Cloning the Website – Webflow

First, let's clone the visible part of the website to your Webflow account. To do this, please follow the steps below.

1. **Click** on the following link to access the website template.

2. Click ***Clone in Webflow*** (Figure 17).
   Note: If you are not logged in, you will need to sign in to your Webflow account and click on the link again.

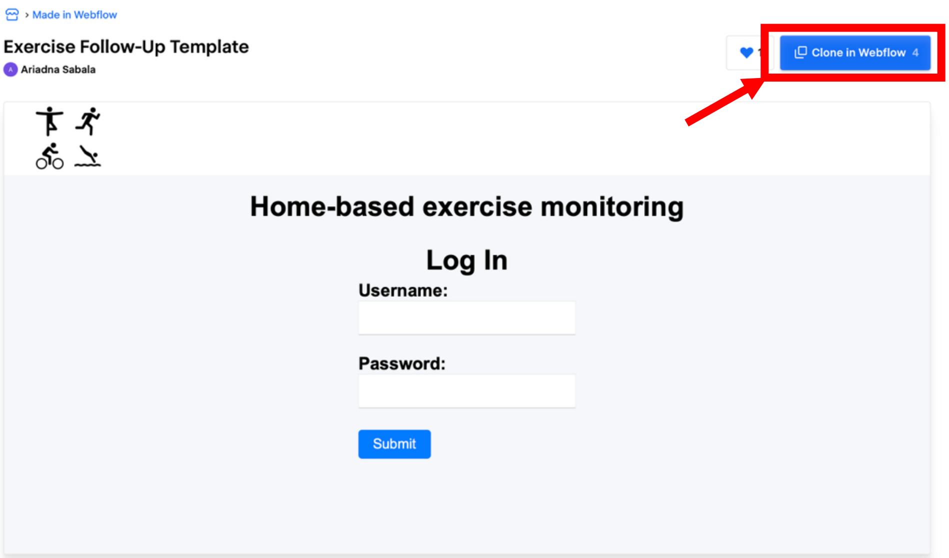

Figure 17. Template of the website

3. On the next page, click the ***Create site*** button.
   - **Enter the name of your website** (This is not a permanent name. If desired, it can be changed later).
   - Click ***Create site***.

   This will immediately create a copy of the website in your workspace.

Notes:

- ***If you have not verified your email*** *(Step 4 of 1.1. Creating a Webflow account), your original verification link has likely expired.*

- ***Then, to verify your email*** *and be able to clone the website, follow the next steps:*
    - *Log in to your Webflow account.*
    - *At the top of the screen, you will see a blue banner that says **'Take a moment to verify your email address by clicking inside the email we sent.'** Click **'Send Email Again'**.*
    - *Check your inbox for the new email. Verify it quickly before it expires again.*
    - *Once verified, the blue banner will no longer appear, and you will be able to clone the website.*

4. **Congrats! You have created a copy of the website in your workspace**.
   You should see something to Figure 18 - **keep this tab open**.
   This the website editor, which is used to make changes to the visual part of the website.

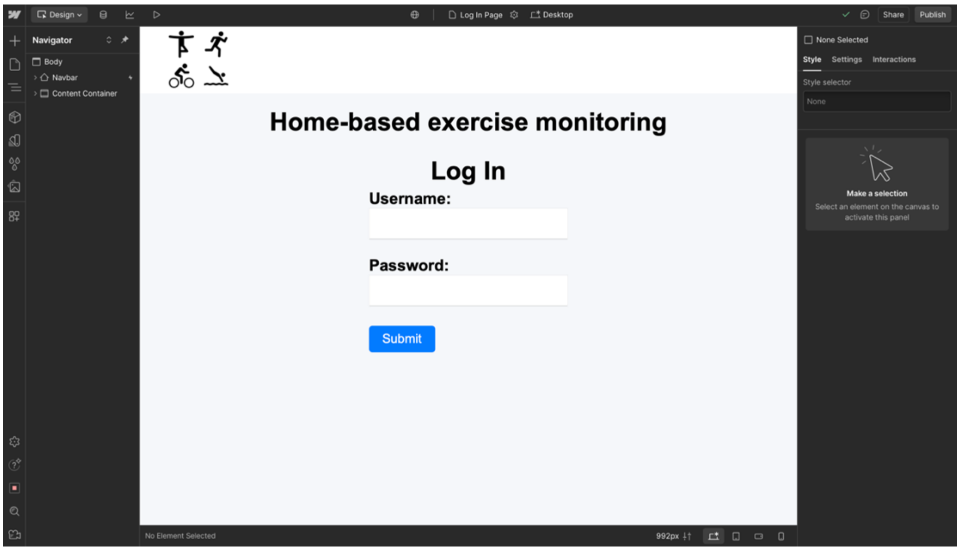

Figure 18. Copy of the website on your workspace

2.2. Cloning the Backend – Xano

Now, we will clone the backend of our website in Xano. To do so, please follow the steps below.

1. Click on this link to clone the backend.
   *Note: Make sure to use **Google Chrome** when accessing Xano.*

2. Click **'Add to your Xano Account'** (Figure 19).
   You will then be requested to **log in**; please do so.

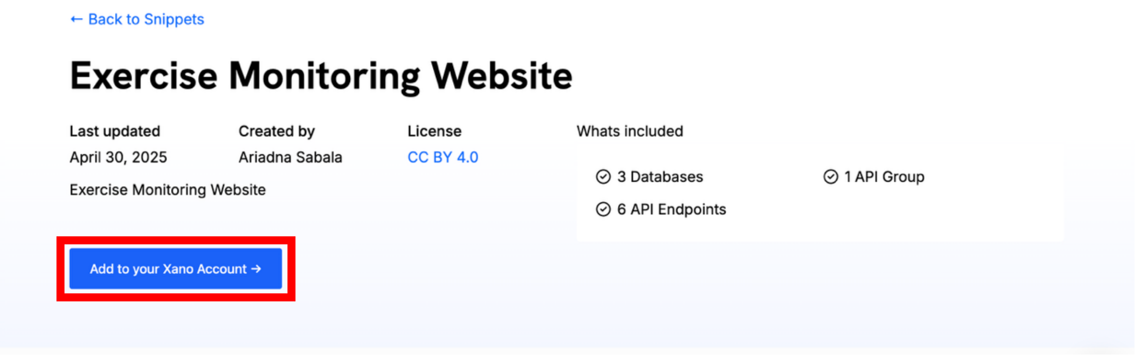

Figure 19. Template of the backend of the website

3. Click on **'Free instance'** (Figure 20).

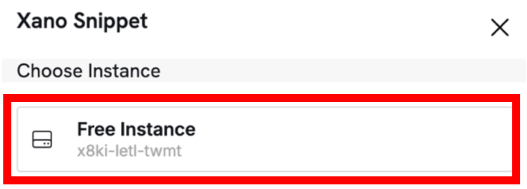

Figure 20. 'Free instance' on Xano

4. Then, click the **'Add to instance'** button in the bottom-right corner of the screen (Figure 21).

5. This may take a moment. Once done, a success message will appear. Click **'Go to instance'** (Figure 22).
    - You will be directed to your Xano workspace.

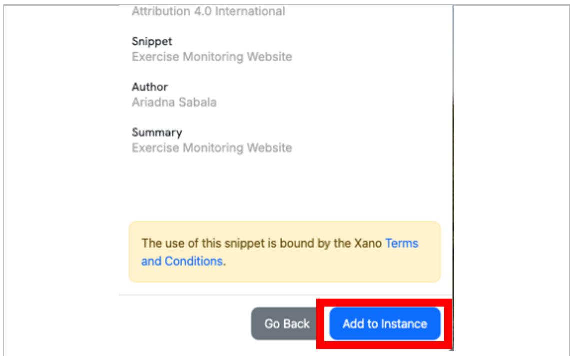

Figure 21. 'Add to instance' on Xano

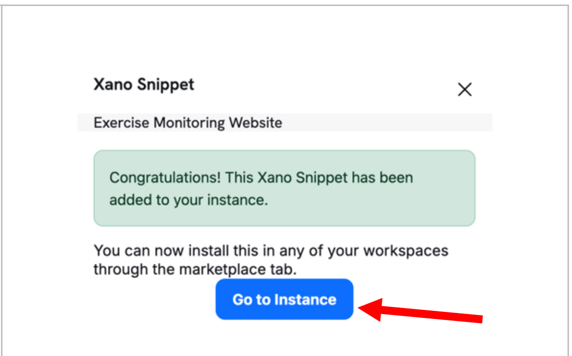

Figure 22. Success message on Xano

6. In the same Xano tab (you can close any other Xano tabs you have open), click ***Marketplace*** on the left side of the screen.

    - Different options will appear, click on ***Purchased*** (Figure 23).

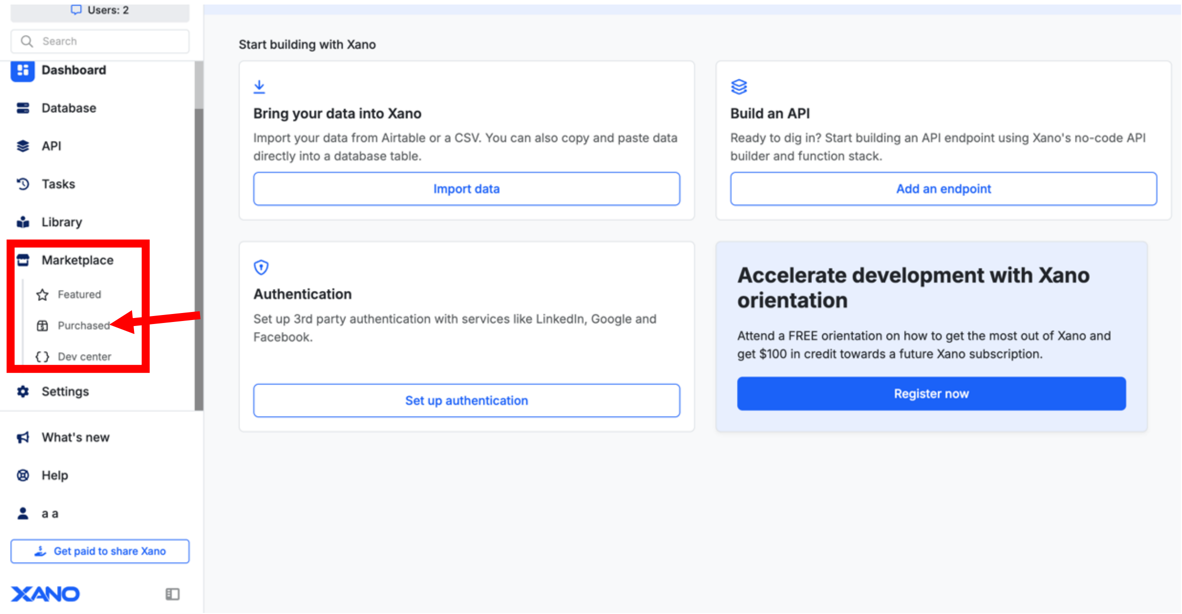

Figure 23. Xano's workspace

7. You are almost done! You should now see something like **Figure 24**.
   - **Click on it**.
   - Then, click ***Install Snippet***, on the right side of the screen (Figure 25).
   - Click the *Install* button again.

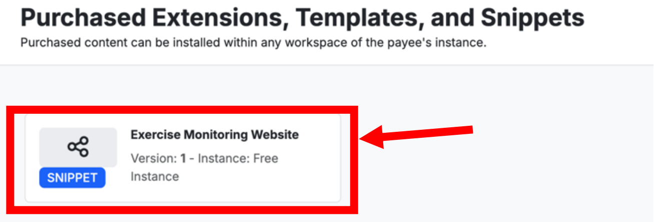

Figure 24. Newly added item appears on 'Purchased' page

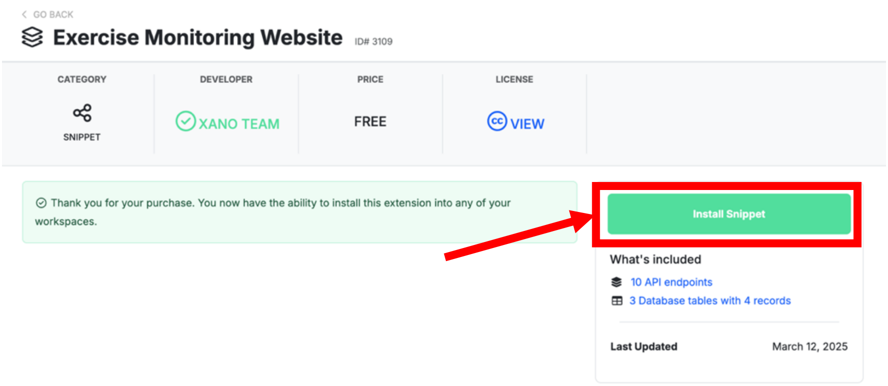

Figure 25. Click on 'Install Snippet' within the newly added item to proceed

8. To verify that you have successfully cloned the backend:
   - Click **'API'** in the left menu.

- A folder named **‘API Webflow website’** should appear (**Figure 26**). This folder contains some links that we will use later to connect Xano with Webflow.
    - If you do not see this folder, repeat Step 7.

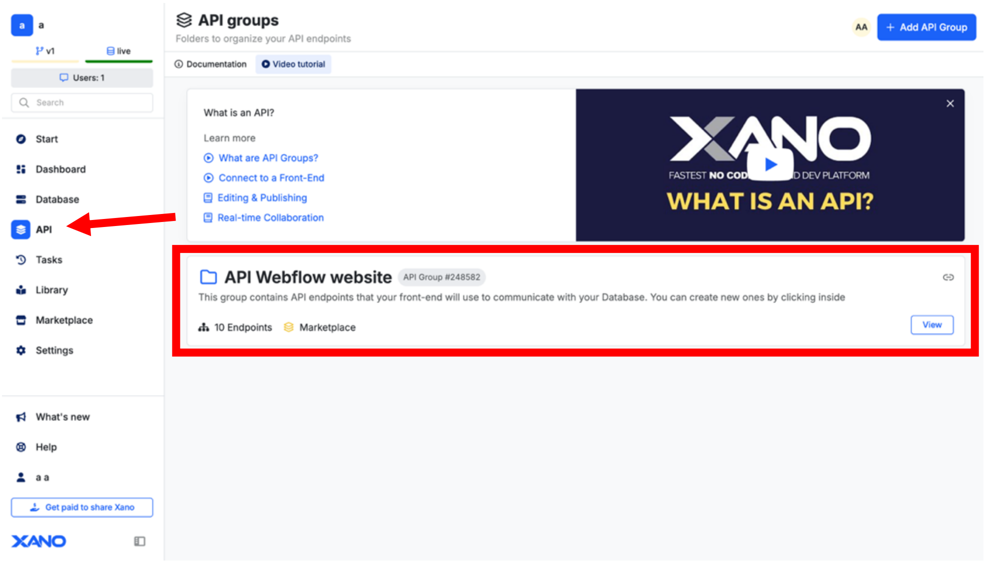

Figure 26. Folder ‘API Webflow website’ with website links in Xano

9. **Congrats!** You have successfully cloned the backend of the website.

2.3. Connecting the Website and Backend (Webflow + Xano)

In this step, we will connect your website (built in Webflow) to your database (in Xano) using special **links**. These links allow the website and the database to communicate with each other. This connection is what makes the website work properly.

Important: *In this section, we will use a feature of Webflow that is available only with a paid plan. Follow the steps below to upgrade your Webflow account, then continue with the linking process. This is the only payment required throughout this manual and for the use of the website.*

Upgrading the website plan

1. In the Webflow tab where you have cloned the website:

    - Hover your mouse over the **‘W’ logo** in the top-left corner of the screen.

    - A **menu icon** (three horizontal lines) will appear, click it (Step 1 – Figure 27).

    - Click on ***Site settings*** (Step 2 - Figure 27).

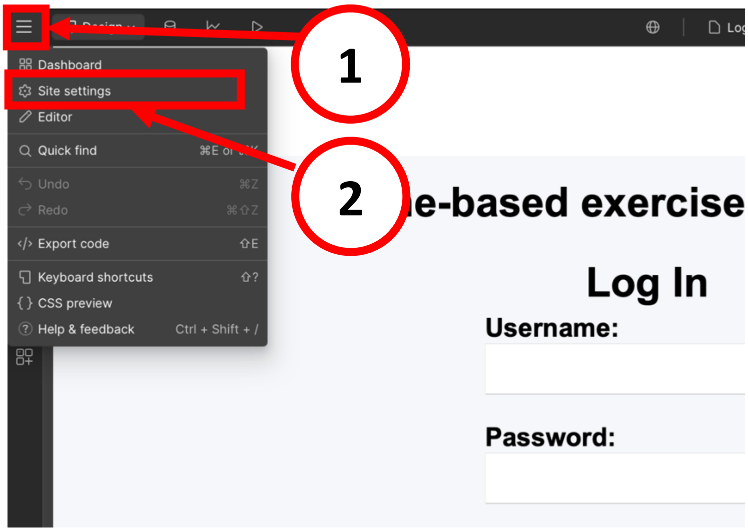

Figure 27. Accessing the Site settings from the website editor

2. Now, in the left menu, go to ***Plans*** **(Step 1- Figure 28)**.
   - The available plans for your website will appear.

   - Select the **'Basic'** plan by clicking on ***Upgrade to Basic*** **(Step 2 – Figure 28)**. *Upgrading to this plan is essential because it will allow us to access a section of Webflow that enables linking the website with Xano.*

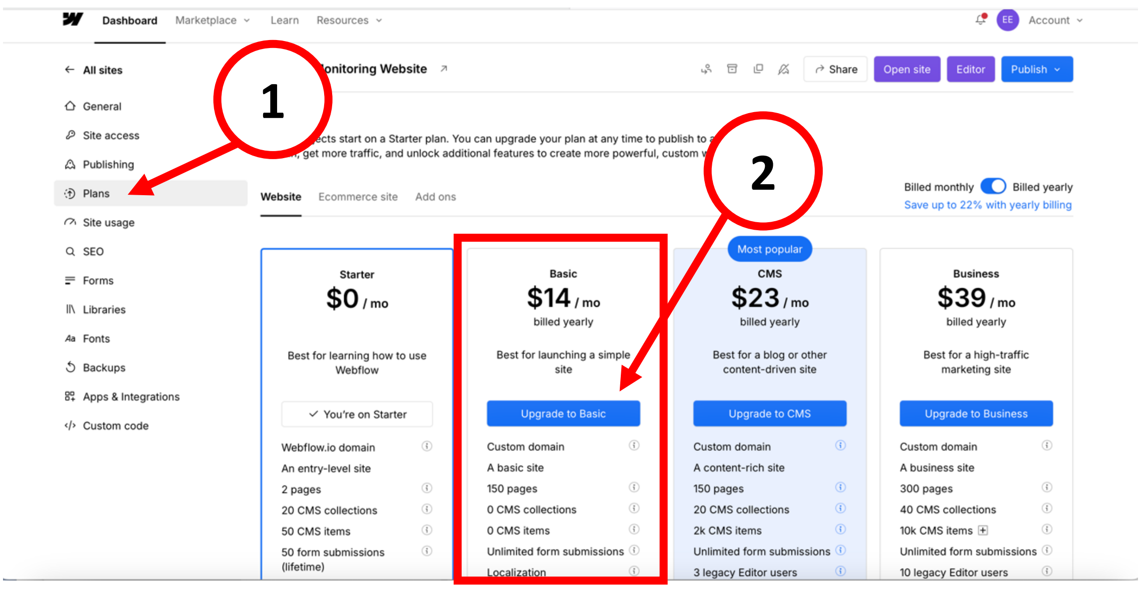

Figure 28. Upgrading the website's plan on Webflow

3. Follow the payment steps.
   Note: <u>You can choose to be billed monthly or yearly. We recommend opting for monthly billing initially</u>. Once confirmed that everything is working, as we will do later in this manual, you can switch to yearly billing, as it's more cost-effective in the long run.

Linking the Website with Xano

Now that you have upgraded the website, we can link it to Xano. Follow the seps below to complete the linking process.

As you follow the instructions, you will copy links from Xano and paste them into Webflow. You will repeat this process a few times. Once finished, Webflow and Xano will be fully connected and ready to work together.

Note: For this process, it is helpful to keep one tab open and logged into Xano (in Google Chrome) and another tab open and logged into Webflow.

1. Go to your **Xano** tab.
   Note: If you have accidentally closed the Xano tab, don't worry - Go to Xano, log in, and click on 'Free Instance' to get back where we were.

   - In the left-side menu, click ***API***.
   - Then, enter the folder **'API Webflow website'** (Figure X).
   - This is a folder we installed when cloning the backend and contains the 'special links' we will use to link in Webflow.

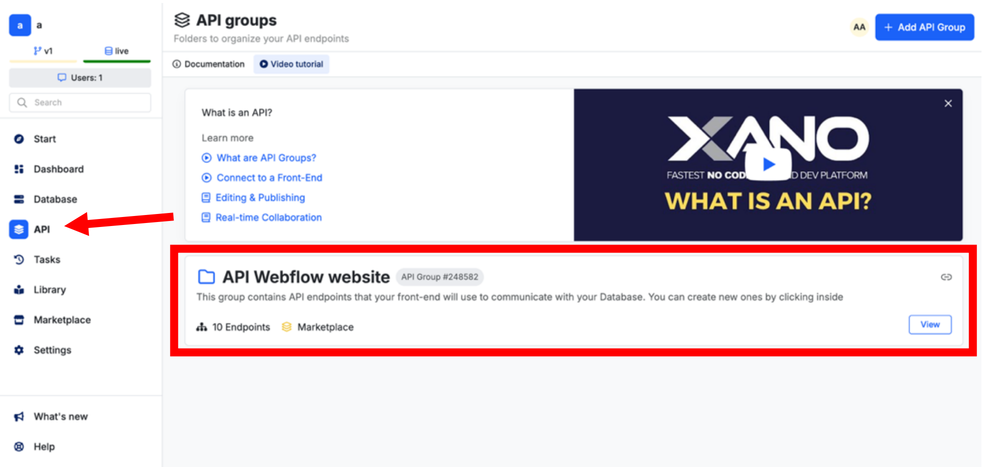

Figure 29. Folder 'API Webflow website' with website links in Xano

   - Inside the folder, you will see something like Figure 30.

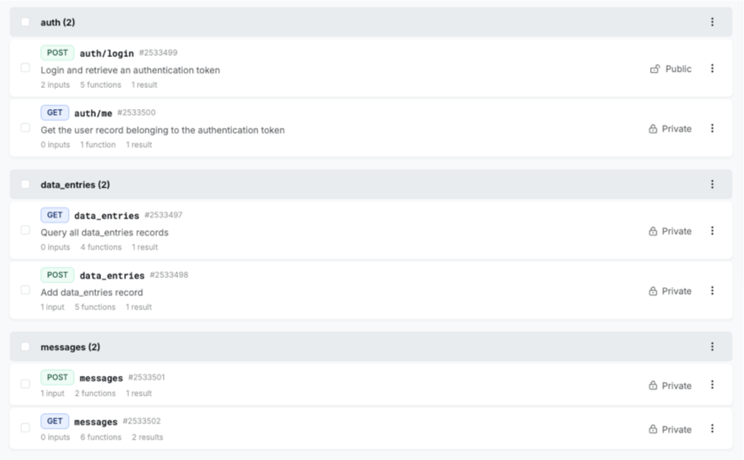

Figure 30. Inside the folder 'API Webflow website' in Xano

2. Inside the 'auth' section, you will see two subsections (Figure 31):
   - **POST auth/login**
   - **GET auth/me**

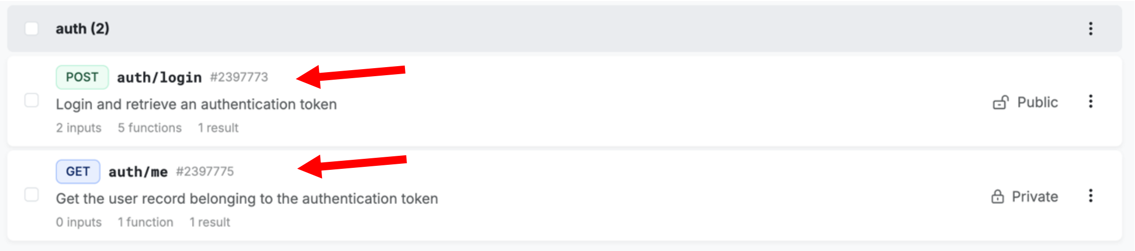

Figure 31. 'API Webflow website' folder in Xano - 'auth' section

3. Click on the '⋮' (three points) next to **POST auth/login (Figure 32)**.
   - Then, click ***Copy Endpoint Link*** **(Figure 32)**. This action copies the first link, which we will use in the next step to paste into Webflow.
   - This link will be one of the connections between Webflow and Xano.

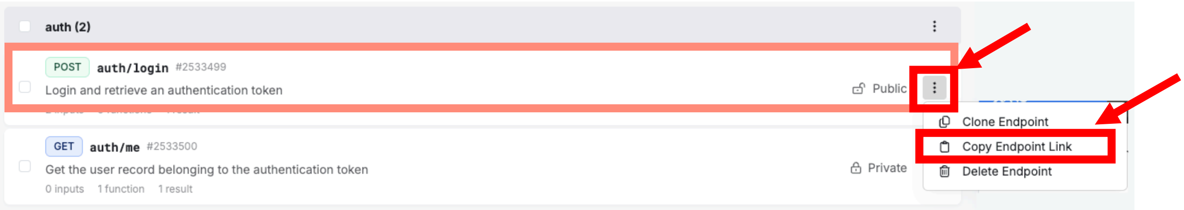

Figure 32. Clicking the three dots next to POST auth/login and 'Copy Endpoint Link'

4. Now, go your Webflow tab. Click on **'Open site'**, to open your cloned website.
   Note: If you have accidentally closed the Webflow tab, don't worry – Go to Webflow, log in, and click on 'Open site' to return to where we left off.

   - Make sure you are in ***Design*** **mode** by checking if **'Design'** appears in the top-left corner of the screen (**Figure 33**).
      - If it doesn't, click there and switch to **Design mode**.

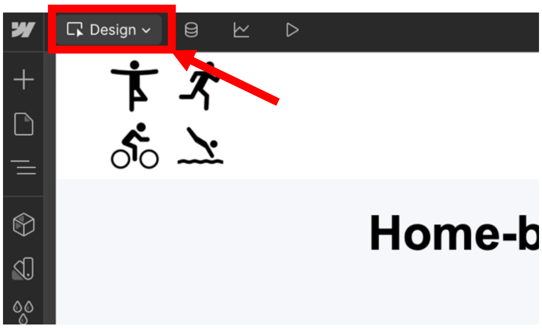

Figure 33. Verifying that you are in Design mode

- In the menu on the left-side of the screen:
  - Click on **‘Pages’ icon** (Step 1 – Figure 34).
  - The will open the Pages menu, which lets you navigate through the different pages of your website.
  - Find the **‘Log In Page’** page and click the **‘ ⚙ ’ icon next to it** (Step 2 – Figure 34).

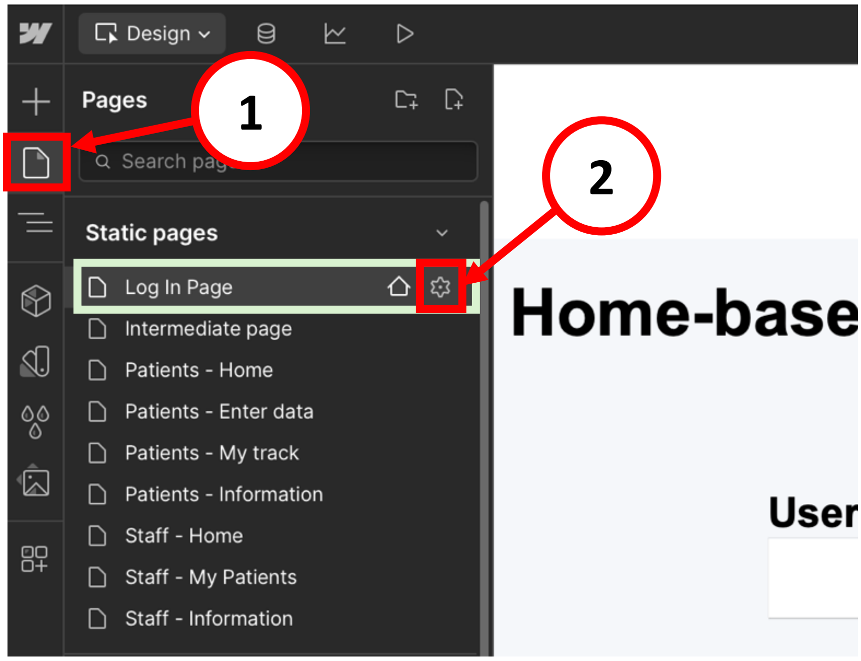

Figure 34. Navigate through your Webflow workspace to open and edit the 'Log In Page' settings.

- Scroll down until you see a section called **‘Custom code’ (Step 1 - Figure 35)**.
  - Don’t worry, you will not need to work with any code!
  - You will see something similar to Figure 35.
  - You will notice the words ‘name’ and ‘content’ written in orange.
  - On the second line, next to ‘content’, **paste (Ctrl + V) the link** we have copied earlier **between the quotation marks (“ ”) (Step 2 - Figure 35)**:

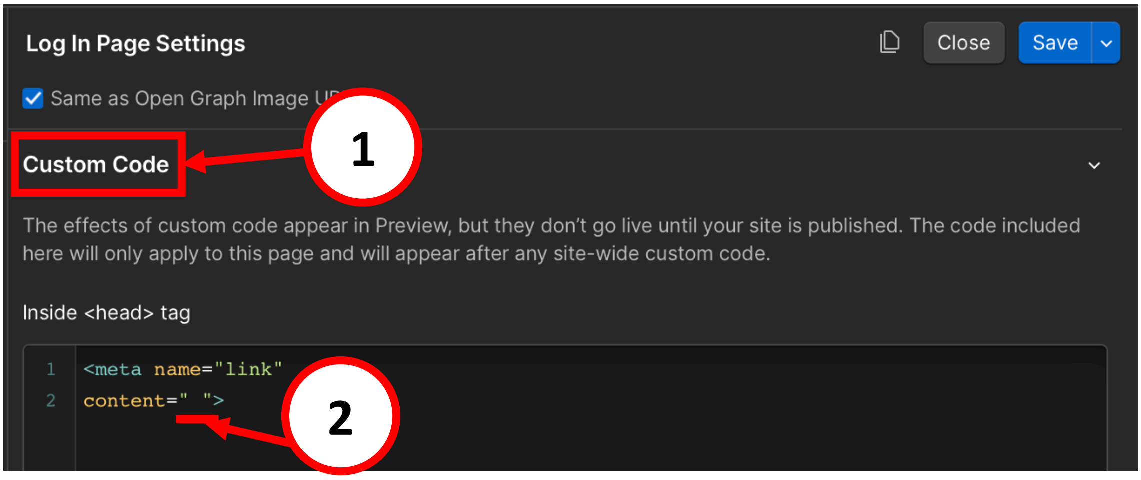

Figure 35. Location of the ‘Custom code’ section and the " " to place the link

- It should look similar to what is shown in **Step 1 - Figure 36**.

- Click ***Save*** on the top-right corner **(Step 2 - Figure 36)**

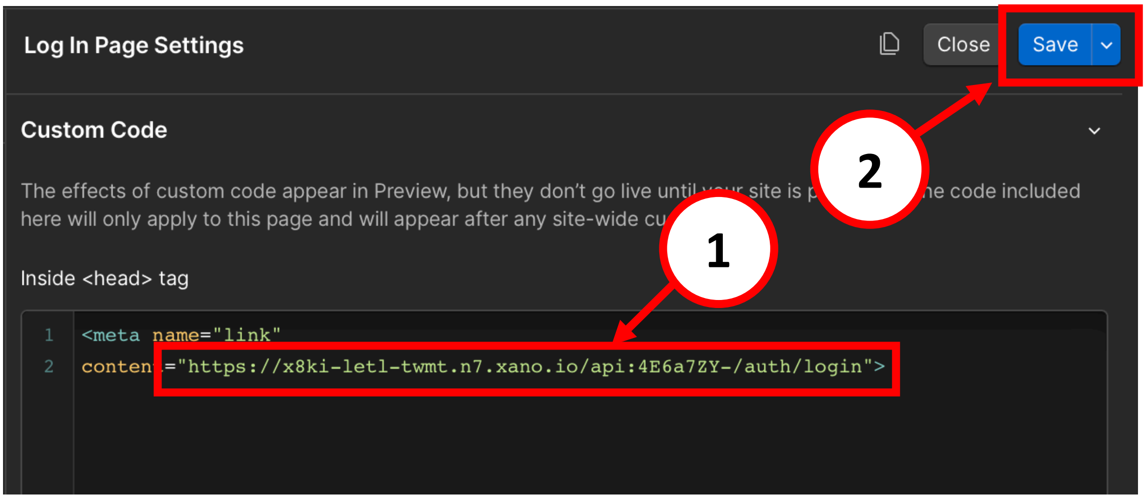

Figure 36. Correct placement of the link between the quotation marks

- **Great job!** You have successfully completed your first linking.

5. Now, let's repeat this process a few more times.
   - Go back to your Xano tab.
     - Click on the ' ⋮ ' (three points) next to **GET auth/me** (Figure 37).
     - Then, click ***Copy Endpoint Link*** (Figure 37), just like you did before. This action will copy the second link, which we will paste into Webflow in the next step.

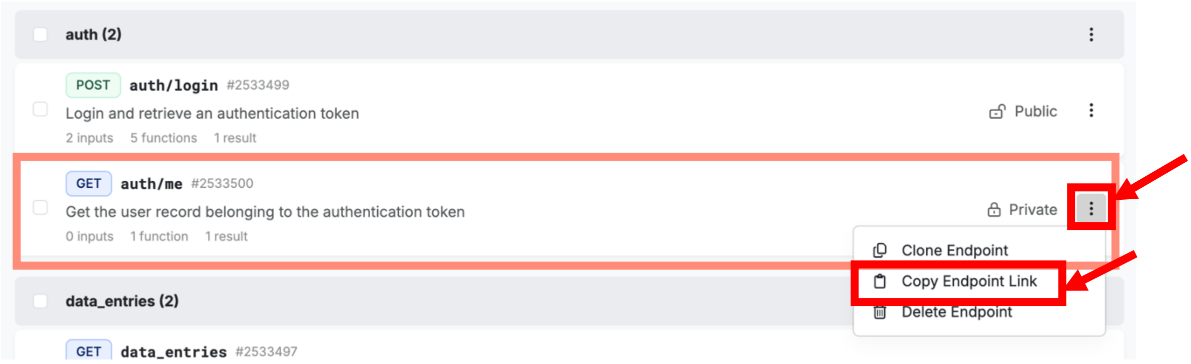

Figure 37. Clicking the three dots next to POST auth/me and 'Copy Endpoint Link'

- Go back to your Webflow tab.
  - Find the **'Intermediate Page'** page and click the **' ⚙ ' icon next to it.**
  - Scroll down and look for the **'Custom Code'** section.
  - As before, you will see the words 'name' and 'content' written in orange.
  - On the second line, next to 'content', **paste (Ctrl + V) the link** we just copied **between the quotation marks (" ")**.
  - Click ***Save*** in the top-right corner.

6. You are almost there! Let’s repeat it one more time.
   6.1. Go back to your Xano tab.
   - This time, look for the **‘data_entries’ section** (Figure 38).

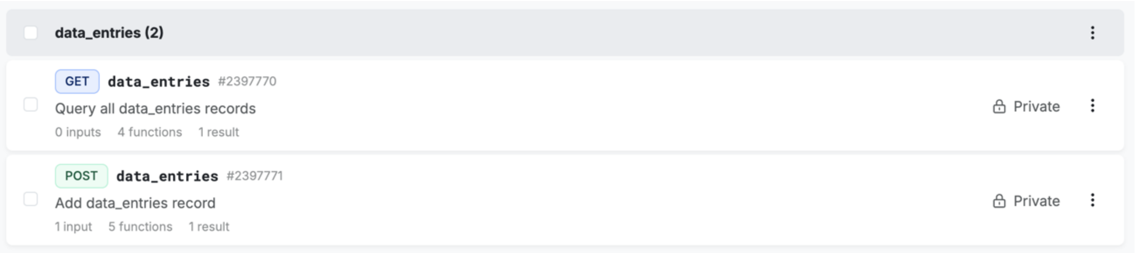

Figure 38. ‘API Webflow website’ folder in Xano - ‘data_entries’ section

- Click on the ‘ ⋮ ’ (three points) next to **GET data_entries** or **POST data_entries** (either option works).

- Then, click ***Copy Endpoint Link***. This action will copy the third link, which we will paste into three different pages in Webflow in the next step.

6.2. Go back to your Webflow tab. We will add this link in three pages.

6.2.1. Find the **‘Patients – Enter data’** page and click the **‘ ⚙ ’ icon next to it.**
   - Scroll down and look for the **‘Custom Code’** section.
   - As before, you will see the words ‘name’ and ‘content’ written in orange.
   - On the second line, next to ‘content’, **paste (Ctrl + V) the link** we copied **between the quotation marks (“ ”)**.
   - Click ***Save*** in the top-right corner.

6.2.2. **Repeat Step 6.2.1 for the ‘Patients – My track’ page.**

6.2.3. **Repeat Step 6.2.1 for the ‘Staff – My patients’ page.**

7. You’re almost there – just one last link to go! Follow the steps below:

   7.1. Go back to your Xano tab.
   - This time, look for the **‘messages’ section** (Figure 39).

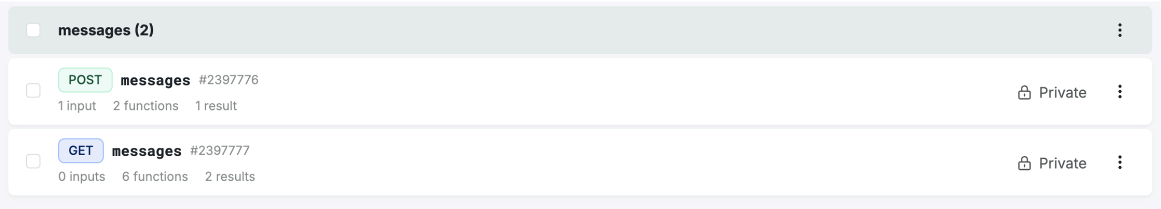

Figure 39. ‘API Webflow website’ folder in Xano - ‘messages’ section

- Click on the ' ⋮ ' (three points) next to **POST messages** or **GET messages** (either option works).

- Then, click ***Copy Endpoint Link***. This action will copy the fourth and last link, which we will paste into two different pages in Webflow in the next step.

7.2. Go back to your Webflow tab.

7.2.1. Find the **'Patients - Home'** page and click the **' ⚙ ' icon next to it.**

- Scroll down and look for the **'Custom Code'** section.
- As before, you will see the words 'name' and 'content' written in orange.
- On the second line, next to 'content', **paste (Ctrl + V) the link** we copied **between the quotation marks (" ")**.
- Click ***Save*** in the top-right corner.

7.2.2. **Repeat Step 7.2.1 for the 'Staff – Home' page.**

8. All set! Your website is now connected to Xano.

Note: If throughout these Steps at any point you do not find the 'Custom code' section, make sure you are in Design mode, as mentioned in Step 4. If you are not in Design mode, you will not be able to see this section.

3. Setting Up the Website's Address

To configure the website's address, there are two options:

- Option A: Use a free Webflow address (recommended for begginers)
- Option B: Buy a custom domain

In this section, follow either Option A or Option B. **We recommend following Option A if you are new to website development.** If you decide to switch to a custom domain later (Option B), you will be able do so at any time.

Option A: Use a free Webflow address

The free Webflow website address looks like this: https://xxx.webflow.io/
You can change the "xxx" to any name you prefer.

1. Hover your mouse over the **'W' logo** in the top-left corner of the screen (Figure 40).

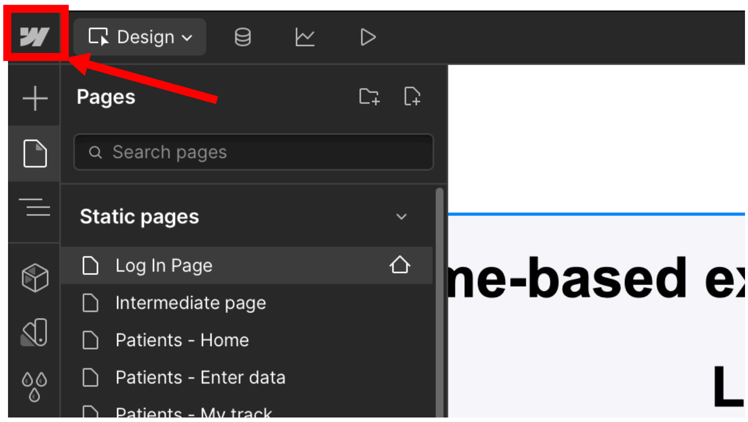

Figure 40. Accessing the Site settings from the website editor

2. A **menu icon** (three horizontal lines) will appear, click it (Step 1 – Figure 41).

3. Click on ***Site settings*** (Step 2 - Figure 41).

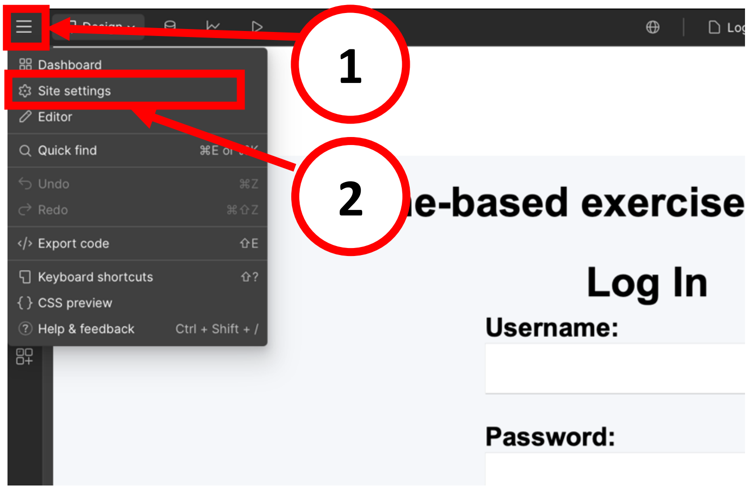

Figure 41. Accessing the Site settings from the website editor

4. Then, in the left menu go to ***Publishing*** (Step 1 – Figure 42).

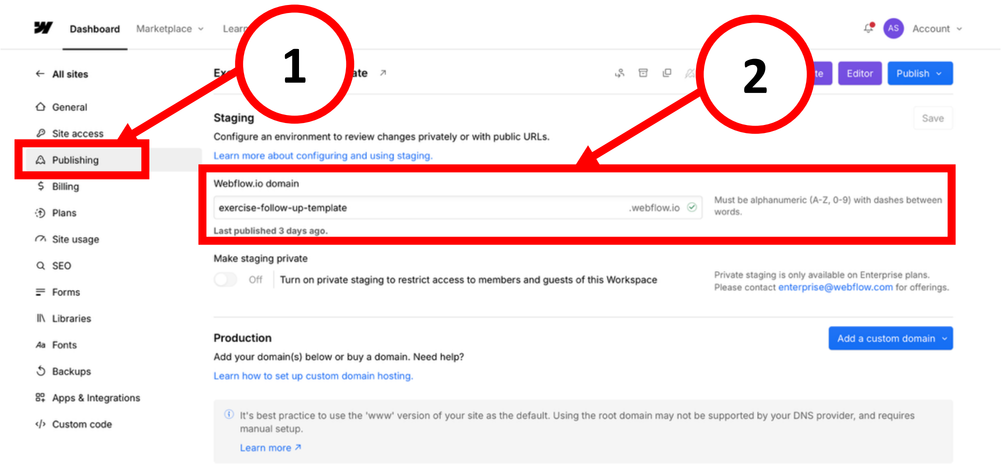

Figure 42. 'Publishing' section in Site settings

5. **Edit the text box** under 'Webflow.io domain' (**Step 2 – Figure 42**).
   - Enter the name you want for your website.
   - You don't need to type ".webflow.io", it is added automatically.
   - For example, in our case, in the text box contains: exercise-follow-up-template

     This means that the website address is:
     https://exercise-follow-up-template.webflow.io/

6. After editing the name, click ***Save*** (top-right corner of the screen).

Option B: Buy a custom domain

If you prefer that your website has different domain, like '.com', '.org', or others, you can buy a custom domain and connect it to your Webflow site.

For more information on how to do this, see this link.

4. Publishing the Website

In this section, we will publish the website to make it available online. Once the website is published, anyone with the link will be able to access it.

Also, whenever you make changes to the website in Webflow, you will use the publish option again to make the changes visible on the live website.

Follow the steps below to publish the website:

1. In Webflow, click ***Open site*** to open your website.

2. Click the **'Publish' button** in the top-right corner of the screen (Figure 43).

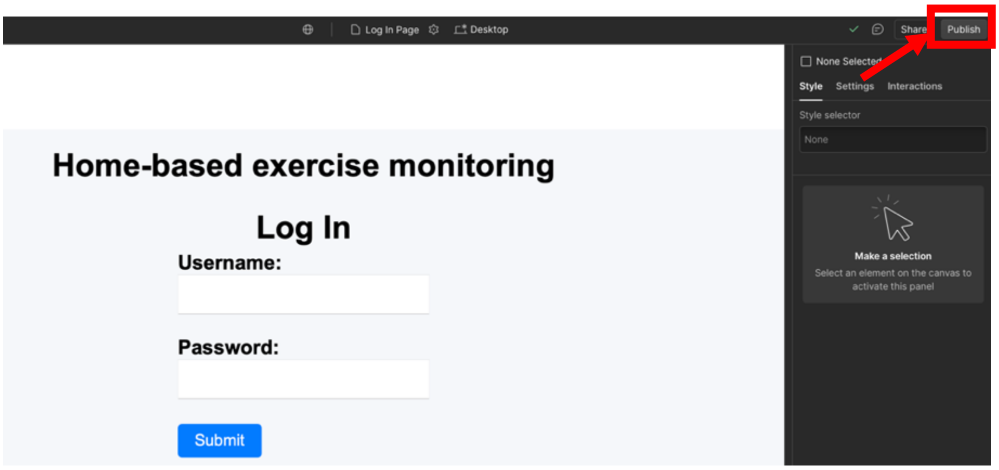

Figure 43. Location of 'Publish' button inside the Webflow's site

3. Click ***Publish to selected domains*** **(Figure 44)** and wait until it is published. Your website is now online!
   - If you followed Option B in the last section: select *Custom domain* and then click *Publish*.

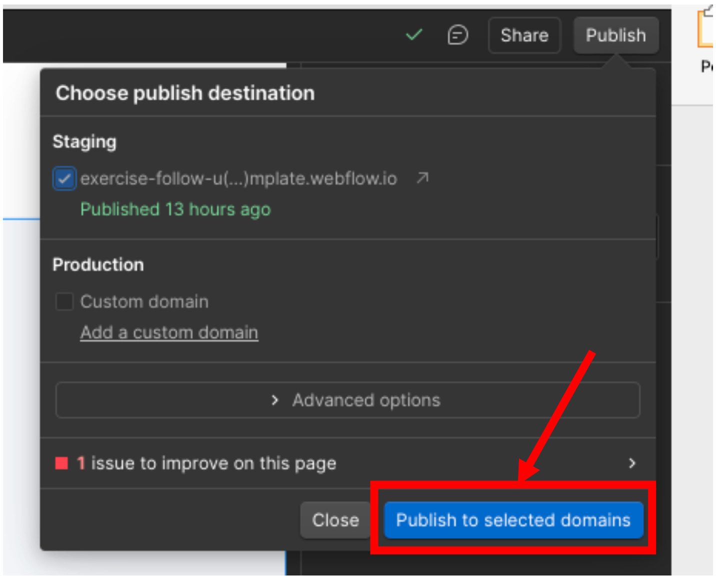

Figure 44. Clicking 'Publish to selected domains' to publish the website.

4. **To access the online website, click the ‘↗’ (arrow) icon** next to the link (Figure 45).

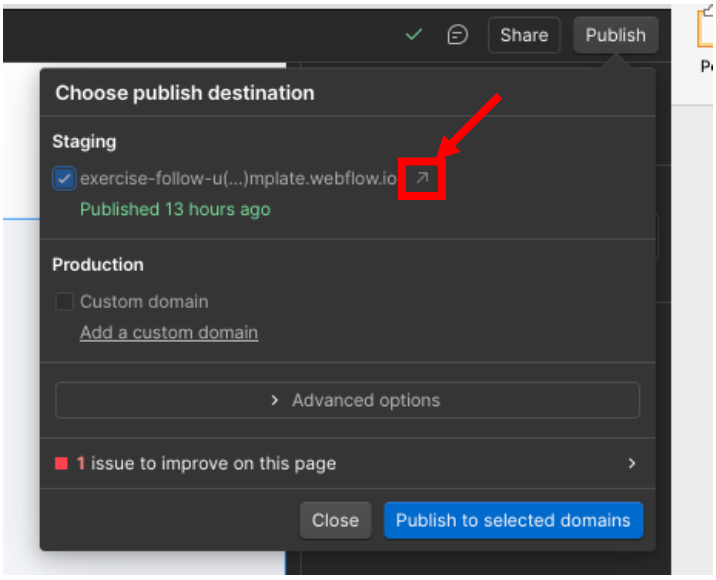

Figure 45. Location of the arrow icon to access the webpage

Note: The link of your site is the link of the newly opened tab, <u>when you are on the log in page</u> (Figure 46).

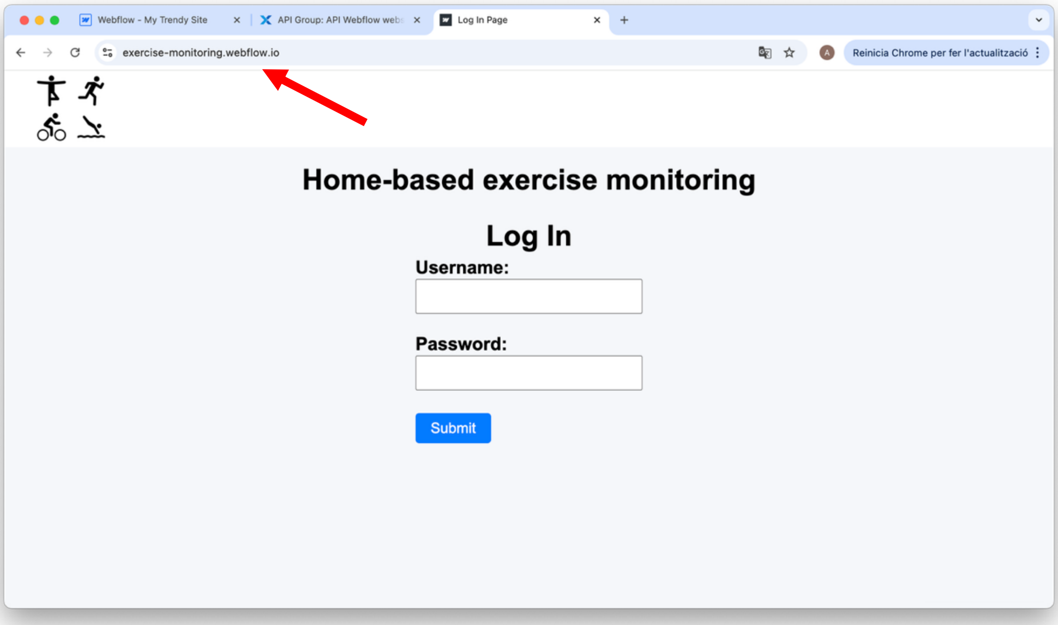

Figure 46. Login page of the website

You will notice that you cannot access the different pages of the website because you do not have a username or password. In the next section, we will learn how to create them.

5. Creating User Profiles

You will notice that on the published website, you cannot access inside the website because you do not have a username or password yet. Let's fix that! In this section, we will go over how to create user profiles.

1. Go to **Xano**.

2. Click ***Database*** **(Step 1 – Figure 47)** in the left menu.
   Note: If you do not find 'Database' because you have just logged into Xano, click on 'Free Instance' to get to where we are.

3. Click on the '**user**' table **(Step 2 – Figure 47)**.
   This table will contain all the users that can log into our website.

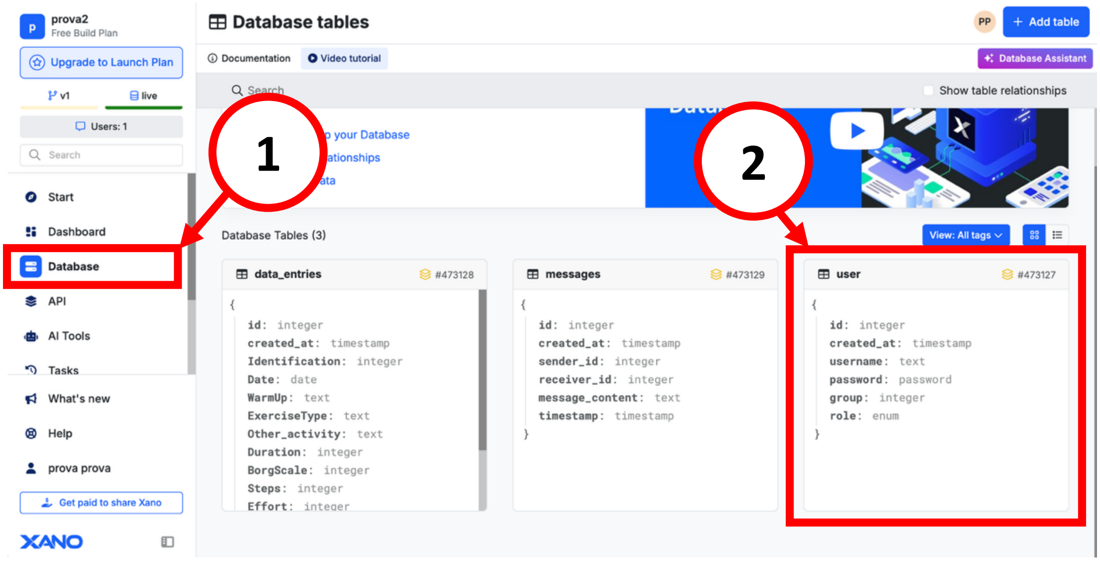

Figure 47. Location of the steps to follow to access the *user* table

4. To create a new user, click **'Add new record' (Step 1 – Figure 48)**.

5. Then, fill following fields **(Step 2 - Figure 48)**:

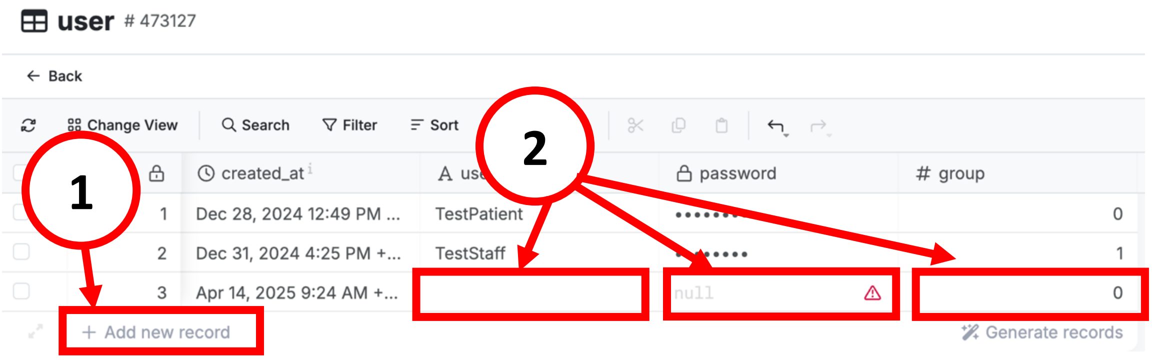

Figure 48. Adding a new record to the 'user' table

- ***username***: Enter the desired username for the profile.

  Note: Avoid using names that indicate whether the user is staff or a patient, or any personal information (See more in section 7. Security Best Practices).
  - *Example of what **not** to use: '1234staff' → This reveals the user is a 'staff' member*
  - *Example of a **better choice**: 'aw156' → This is a non-identifiable username.*

- ***password***: Set a password for the user.
  To create a strong password, it should include:
  - At least **8 characters**
  - At least **1 number**
  - At least **1 symbol**
  - At least **1 lowercase letter**
  - At least **1 uppercase letter**

  The password cannot include accented characters.

  Note: Make sure to write it down somewhere. If you forget it, you will not be able to view the password later; you will only be able to change it.

- **'group'**: By default, a '0' will appear on this field. Click on it and **update the value based on the type of user**:
  - Enter '**0**' if the user is a **patient**
  - Enter '**1**' if the user is **staff**

6. We recommend that you **try logging in on your website with the username and password** you just created to ensure they work.
   - The published website is the one we accessed in last step of the last section.
   - If you cannot log in, don't worry, as we will verify the this later in section *6.1. Verifying Login Functionality*

7. Congrats! You have successfully created new user profiles for your website.

Test Users

The '**user'** table in Xano initially contains two users. These are their usernames:
- TestPatient
- TestStaff

These users will be useful for testing purposes throughout this manual. Update their passwords: **Double-click on the password field and set a password** you will remember, as we will use these users later in Section *6. Verifying Website Functionality* .

What to Do if a User Forgets Their Password

If a user forgets their password, you will need to change it to a new one in Xano:

1. Go to **Xano**.
2. In the left menu, go to **'Database'**.
   Note: If you do not find 'Database' because you have just logged into Xano, click on 'Free Instance' to get to where we are.
3. Open the **'user'** table.
4. Find the username of the User in the table.
5. Then, **double-click its password** field and **enter a new password**.
6. Give the new password to the user.

How to Find the Username–ID Patient Equivalence

As a staff member, you will not see the patient's name or username directly on the website, you will see their **ID**.

So, how can you find the equivalence between a patient's username and their ID?

1. Go to **Xano**.
2. In the left menu, go to **'Database'**.
   Note: If you do not find 'Database' because you have just logged into Xano, click on 'Free Instance' to get to where we are.
3. Open the **'user'** table.
4. **Locate the username** of the patient **(Step 1 – Figure 49)**
5. **Find the corresponding number in the 'id' column (Step 2 – Figure 49)**.
   This number represents the patient's ID.

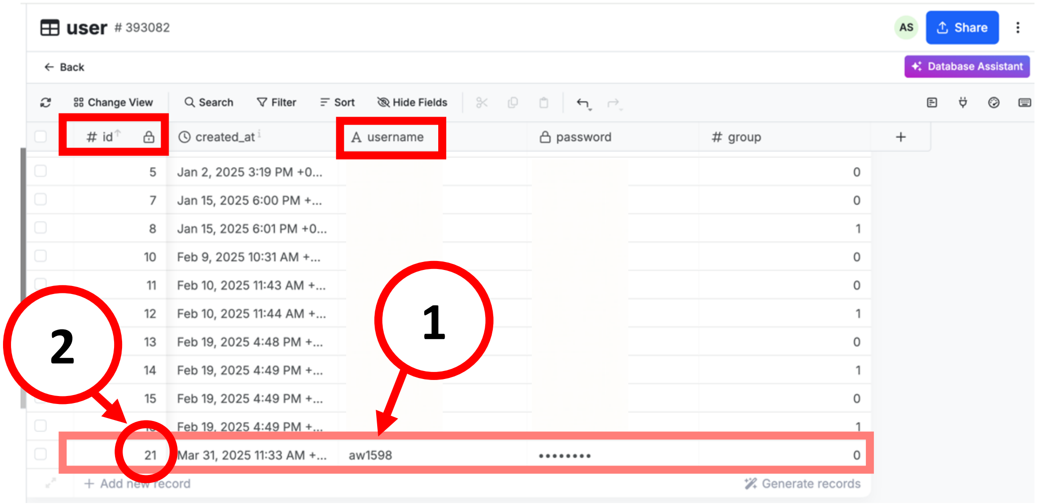

Figure 49. Identifying a patient's ID in the Xano database.

Note: We recommend maintaining a separate document with a table containing the ID, patient usernames, and their corresponding names. This will save time and prevent the need to check Xano repeatedly. For example:

Name	ID	Username
George	21	aw1598
…	…	…

6. Verifying Website Functionality

In this section, we will **verify that all website functionalities are working** properly.

To do this, **we will use the 'test patient' user profile and the 'test staff' user profile** mentioned in the last section. For more details, refer to the *Test Users* section.

6.1. Verifying Login Functionality

Go to the link of your published website and log in using the username and password of the 'test patient'. Can you log in?

- **If yes:**
  You should see something like **Figure 50**. This means the login functionality is working properly. **Skip to section *6.2. Verifying Messaging Functionality*.**

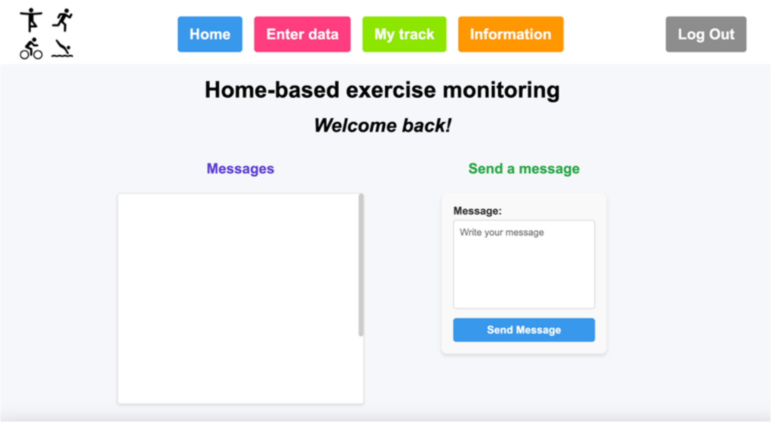

Figure 50. Home page of a newly logged-in user

- **If yes but stuck on an intermediate page (like Figure 51):**
  This suggests that ***Step 5 in Linking the Website with Xano*** may not have been completed correctly.
  - **Repeat** Step 5.
  - Then, **publish** the website to apply and save your changes (For detailed instructions, refer to section *4. Publishing the Website*).
  - **Refresh** the website and **attempt to log in again**.

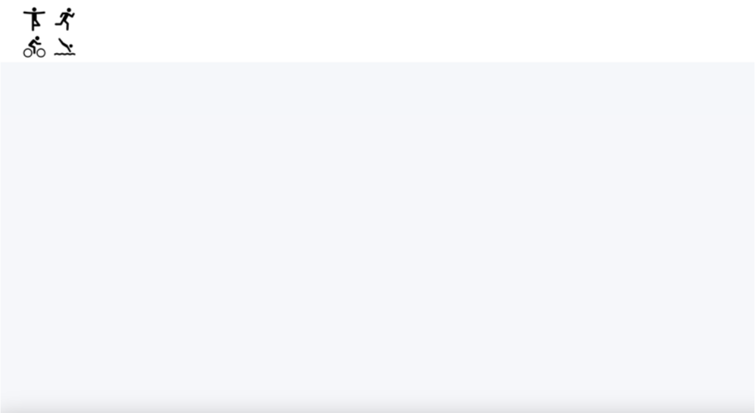

Figure 51. Intermediate page (incomplete log in functionality)

- **If no:**

    - And a banner appears on your screen that says **'Invalid username or password' (Figure 52)**:
      This means that you are either introducing the wrong username or password.
      Please revisit section ***5. Creating User Profiles*** and make sure you are entering the right username and password.

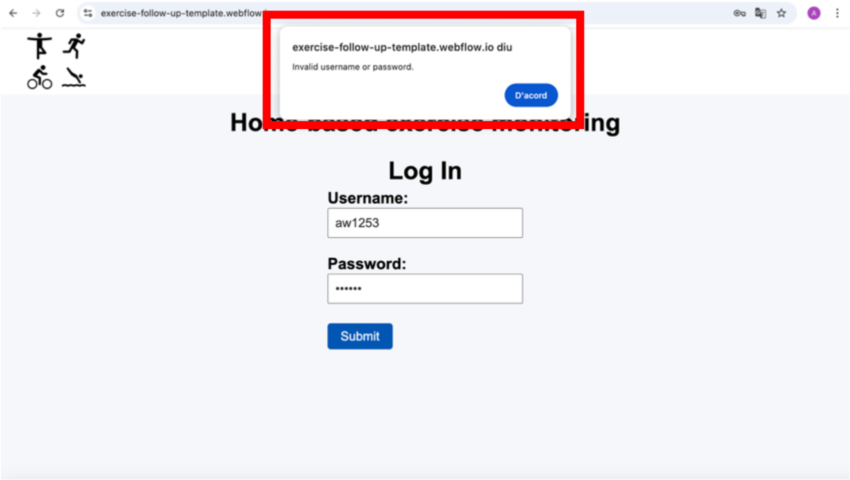

Figure 52. Screenshot of banner 'Invalid username or password'

    - **If no banner appears** when clicking the 'submit' button but you are still unable to log in:
      This indicates that the ***Steps 3 and 4 in 'Linking the Website with Xano'*** may not have been completed correctly.
        - **Repeat these steps** (Steps 3 and 4)
        - Then, **publish the website** to apply and save your changes (For detailed instructions, refer to section *4. Publishing the Website*).
        - **Refresh** the website and **attempt to log in again**.

6.2. Verifying Messaging Functionality

Now, we are going to verify the messaging functionality for patients and for staff.

Verify Messaging for a Patient User

1. **Log in** using the username and password of the **test patient** user profile.

2. In the Home page, **try writing a message** in the message box **and sending it** (Figure 53):

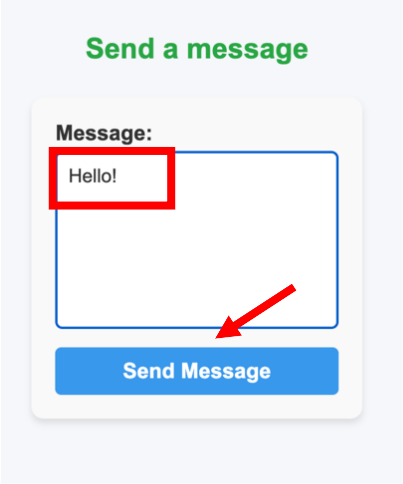

Figure 53. Sending a message in the message box

- **If the message appears in the left message inbox (Figure 54):**
  The messaging functionality for patients is working properly. **Skip to section *Verifying Messaging for a Staff User*.**

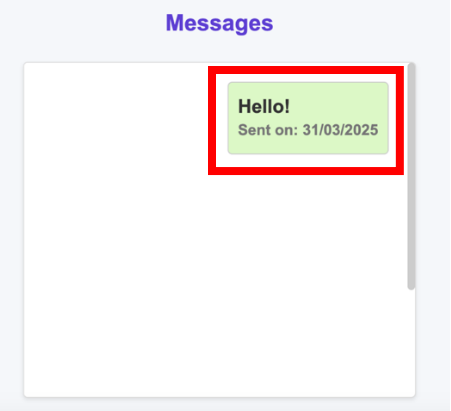

Figure 54. Sent message appearing in the message inbox

- **If the message does not appear:**
  This indicates that the ***Steps 7.1 and 7.2.1 in 'Linking the Website with Xano'*** section may not have been completed correctly.
  - **Repeat these steps** (Steps 7.1 to 7.2.1)
  - Then, **publish the website** to apply and save your changes (For detailed instructions, refer to section *4. Publishing the Website*).
  - **Refresh** the website and **try sending a message again**.

Verify Messaging for a Staff User

1. Log out of the **test patient** user profile.

2. Log in using the **test staff** user profile.

- **If the message sent by the test patient in the previous section appears (Figure 55):**
  Great! This confirms that staff users can properly receive and send messages to and from patients. **Skip to section** *6.3. Verifying Data Submission and Visualization*.

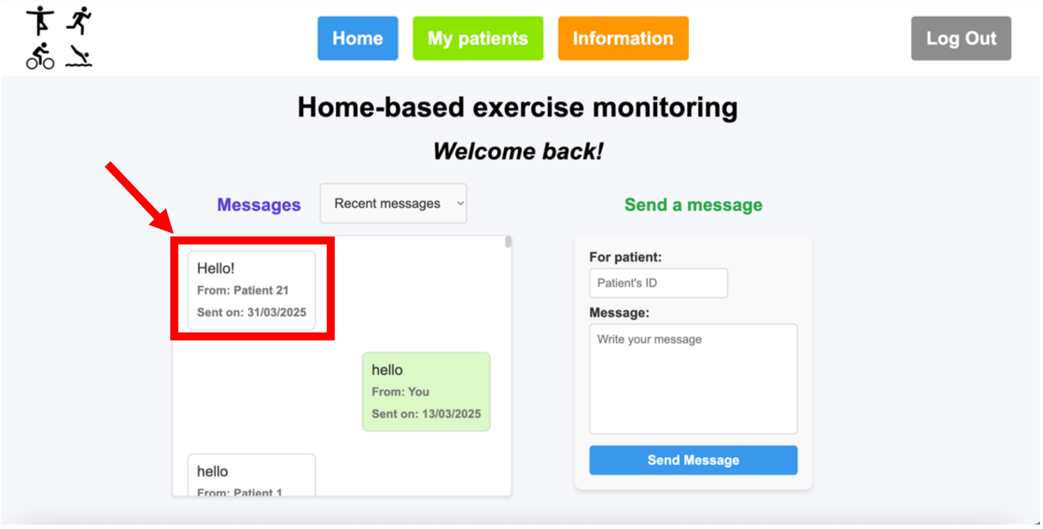

Figure 55. Home page of a logged-in staff user displaying the message received

- **If the message sent by the test patient in the previous section does not appear (Figure 56):**
  This suggest that *Steps 7.1 and 7.2.2 in 'Linking the Website with Xano section'* may not have been completed correctly.
  - **Repeat the steps** (*Steps 7.1 and 7.2.2*)
  - Then, **publish the website** to apply and save your changes (For detailed instructions, refer to section 4. Publishing the Website).
  - **Refresh** the website and **check if the message appears**.

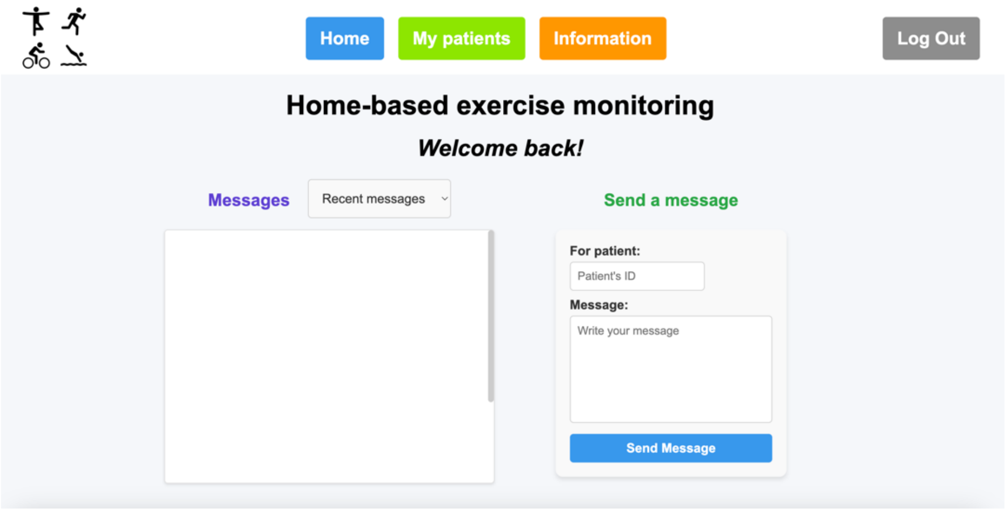

Figure 56. Home page of a logged-in staff user with no messages displayed

6.3. Verifying Data Submission and Visualization

Finally, let's verify that the data submission and visualization works.

Verifying Data Submission and Visualization for a Patient User

1. Log out of the **test staff** user profile.

2. Log in using the **test patient** user profile.

3. **Click 'Enter data'** in the top menu to navigate to the data entry page.

4. Fill out the form as you want and click 'Send data' (Figure 57).

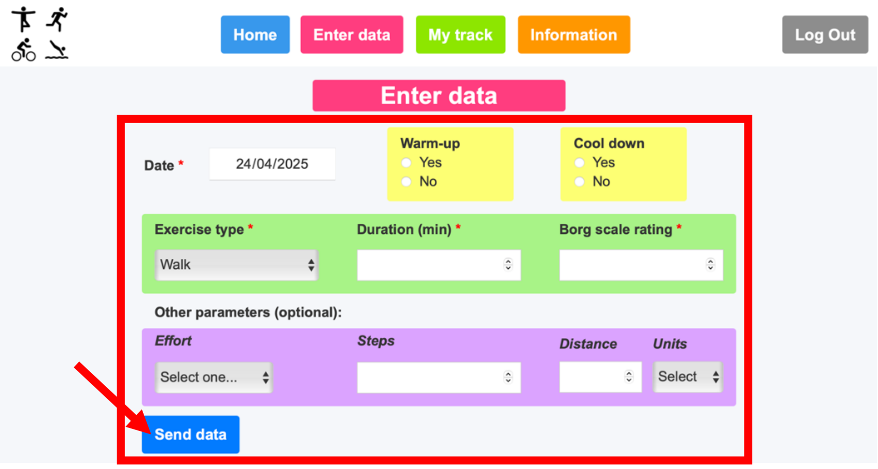

Figure 57. Filling out the form on the 'Enter data' page

- **If the form is submitted successfully (Figure 58):**
  This means that the data submission is working properly. **Go to Step 5.**

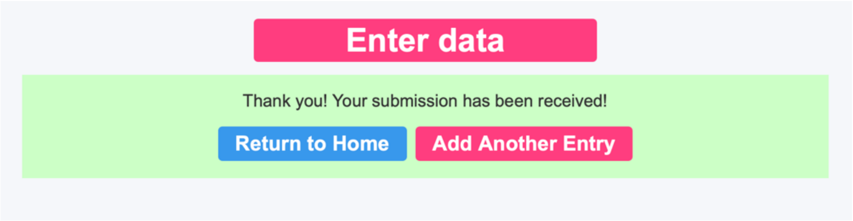

Figure 58. Confirmation page after successfully submitting a form

- **If the form is not submitted (error message like in Figure 59):**
  You will see an **error message** like in **Figure 59**.
  This indicates that the ***Steps 6.1 and 6.2.1 in 'Linking the Website with Xano'*** section may not have been completed correctly.
  - **Repeat these steps** (Steps 6.1 and 6.2.1)
  - Then, **publish the website** to apply and save your changes (For detailed instructions, refer to section *4. Publishing the Website*).
  - **Refresh** the website and **try submitting the form again**.

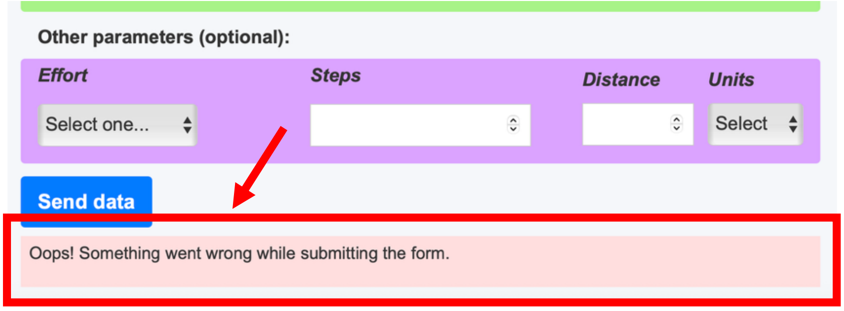

Figure 59. Error message displayed when there is an issue with data submission functionality

5. Now, go to **'My track'** page (by clicking the button in the top menu).

6. **Check if the data entry submitted** before **appears** on the screen.

   - **If yes (Figure 60):**
     Perfect! This confirms that patients can properly enter and visualize their data. **Go to section** ***Verifying Data Visualization for a Staff User***.

   - **If not (Figure 61):**
     This indicates that the Steps 6.1 and 6.2.2 in ***'Linking the Website with Xano'*** section may not have been completed correctly.
     - **Repeat these steps** (Steps 6.1 and 6.2.2)
     - Then, **publish the website** to apply and save your changes (For detailed instructions, refer to section *4. Publishing the Website*).
     - **Refresh** the website and **try visualizing the data again**.

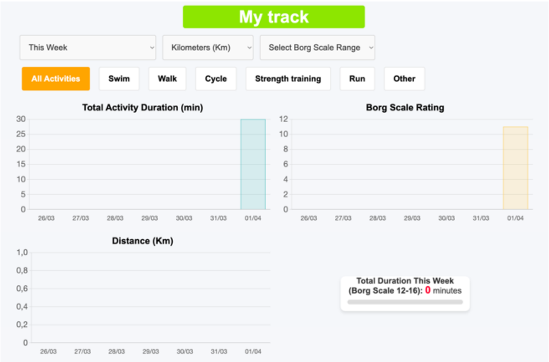

Figure 60. Example of a submitted data entry displayed on the 'My Track' page

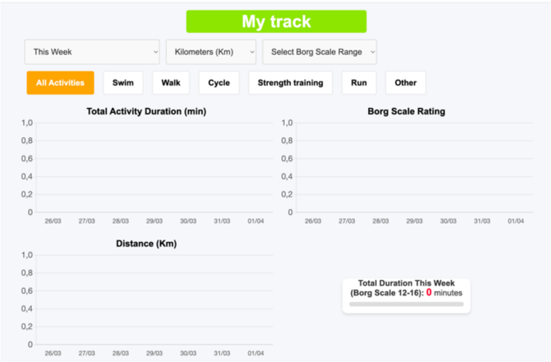

Figure 61. 'My Track' page with no entries displayed

Verifying Data Visualization for a Staff User

1. Log out of the **test patient** user profile.

2. Log in using the **test staff** user profile.

3. **Click 'My patients'** in the top menu, to navigate to the page where the data entries of the patients appear.

4. In the **'Select a patient' dropdown**, select the patient with the **ID** corresponding to the **test patient user** (See ***How to Find the Username–ID Patient Equivalence***)

5. Check if the data entry you submitted earlier by the patient profile appears on the screen:

   - **If yes:**
     Great! This means staff users can properly view patient data entries.

   - **If not:**
     This suggests that the Steps 6.1. and 6.2.3 in ***'Linking the Website with Xano'*** section may not have been completed correctly.
     - **Repeat these step** (Step 6.1 and 6.2.3)
     - Then, **publish the website** to apply and save your changes (For detailed instructions, refer to section *4. Publishing the Website*).
     - **Refresh** the website and **try visualizing the data again**.

7. Security Best Practices

No personal or clinical data related to the patient's health is introduced into the website, only anonymous data on daily exercise.

However, there are still some measures you should follow to protect user information.

As mentioned in the ***5. Creating User Profiles*** section, the usernames and passwords should meet the following guidelines:

- Usernames:
    - Do not include personal information.
    - Do not reveal whiter the username belongs to a patient or a staff member.
    - Use random combinations of letters and numbers.

- Passwords:
  To create strong passwords, they should include:
    - At least **8 characters**
    - At least **1 number**
    - At least **1 symbol**
    - At least **1 lowercase letter**
    - At least **1 uppercase letter**

Additionally, the document linking each patient to their ID and username, as recommended in section ***How to Find the Username–ID Patient Equivalence*** should be kept in a private file. It can be stored on a local computer (not in the cloud) or as physical copy outside any computer.

As for data stored in Xano, periodic reviews should be conducted to ensure that patient information that is no longer needed is not stored online. To learn how to delete a user's data, refer to section ***6. Deleting a User's Data*** in the **Annex**.

Website Template Customization

Before proceeding with the customisation of the website template, you must have completed all the sections within *Webflow Template Implementation*.

This section contains two examples to help you learn how to customize the website template:

- **Example 1:**
  In this example, we will **keep the website for physical rehabilitation monitoring**, and we will apply some changes:
  - Change the website's logo
  - Remove the option for patients to send messages
  - Modify and add activity types in the *Exercise Type* dropdown (on the *Enter data* page)
  - Delete the existing information documents and add new ones

- **Example 2:**
  In this example, we will modify the template and turn it into a **patient behaviour and wellbeing monitoring site**, where we will track:
  - Objective behaviours: medication compliance, sleep time, exercise duration, smoking, alcohol drinking, and eating
  - Subjective perceptions: sleep quality, exercise effort and general wellbeing

  Additionally, we will:
  - Change the website's logo
  - Change and add new information documents

Choose the example that best suits your needs and follow it.
For additional modifications, you can refer to the sections in the Annex.

Example 1: Adapting the Physical Rehabilitation Monitoring Template *(30min – 45min)*

As mentioned before, in this example we will:

- Change the website's logo
- Remove the option for patients to send messages
- Modify and add activity types in the *Exercise Type* dropdown (on the *Enter data* page)
- Delete information documents and add new ones.

Note: Every time you open your website in Webflow (Figure 62), it is important to make sure you are in ***'Design' mode****, to be able to modify the website.*

- *To check it, look if* ***'Design'*** *appears in the top-left corner of the screen (Figure 63).*
- *If it doesn't, click there and switch to* ***'Design' mode****.*

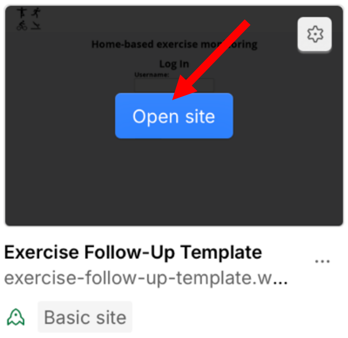

Figure 62. Opening the website

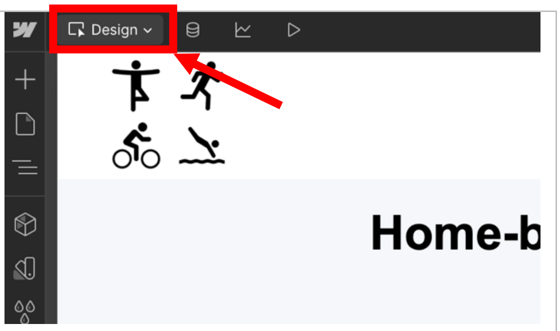

Figure 63. Verifying that you are in Design mode

1. ***Changing the website's logo***
   1. **Choose the logo image** you would like to use and save it in your computer.

   2. Next, follow all the steps in section ***1. Changing the website's logo*** in the **Annex**.

   3. Once you have completed the steps, you will have successfully updated the logo of the website with your chosen image.

2. ***Restricting patients from sending messages***
   1. This will prevent patients from sending messages, while still allowing staff to send them messages.

   2. To apply it, follow all the steps in section ***3.1. Restricting patients from sending messages***.

3. ***Modifying the Exercise Type dropdown***
   1. Add the option **'Dance' (or another activity)** to the Exercise Type dropdown on the 'Enter data' page (patient view).

2. To do so, follow the steps in section *Editing Exercise Type Options*.

3. If you also want to delete some of the existing activities, you can learn how to do so in the same section.

4. Managing information documents

1. The website has two 'Information' pages:
   - One that only patients can see: ***Patients – Information*** page

   - And one that only staff members can see: ***Staff – Information*** page

2. To view the information documents available for patients, log into the published website as a patient and click on the documents on the *Information* page.
   You can do the same with a test staff user to view their documents.

3. Now, follow the steps in steps in section *How to delete a document, image, link or video* to delete an information document.

4. Perfect! Now, let's add a new one. **Choose an information document, image, link or video** you would like to add to the website.

5. **Decide** whether you want to add the document to the patient information page, the staff information page, or both.

6. If you want to add it to:

   - The patient information page:
     - Follow the steps in section *How to add a document, image, link or video* and, in Step 2, click ***Patients – Information*** page.

   - The staff information page:
     - Follow the steps in section *How to add a document, image, link or video* and, in Step 2, click ***Staff – Information*** page.

   - Both pages:
     - Follow the steps in section *How to add a document, image, link or video* **twice**:
       - First time: In Step 2, click ***Patients – Information*** page.
       - Second time: In Step 2, click ***Staff – Information*** page.

Once you have followed these steps, you will have successfully adapted the physical rehabilitation monitoring website.

Example 2: Modifying the Website for Patient Behaviour and Wellbeing Monitoring *(1h 15min – 1h 30min)*

In this example we will modify the website and convert it into a patient behaviour and wellbeing monitoring website. To do so, we will make the following modifications:

- Change the website's logo
- Change the form in the 'Enter data' page
- Adapt the graphs in the 'My track' page (patient's view) and 'My patients' page (staff view)
- Delete information documents and add new ones.

The new 'Enter data' form will look like Figure 64.

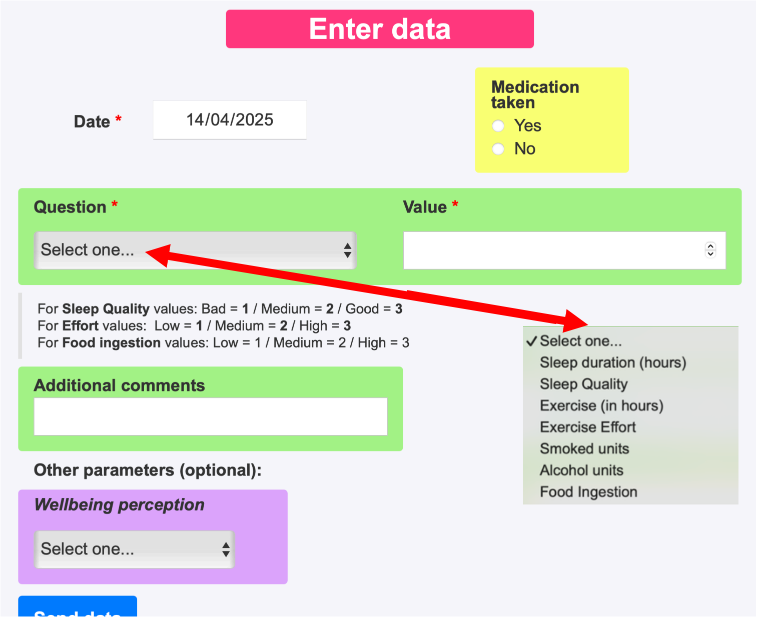

Figure 64. 'Enter data' form on the wellbeing monitoring website

Pages with graphs (the 'My track' page for patients and the 'My patients' page for staff) will look like Figure 65.

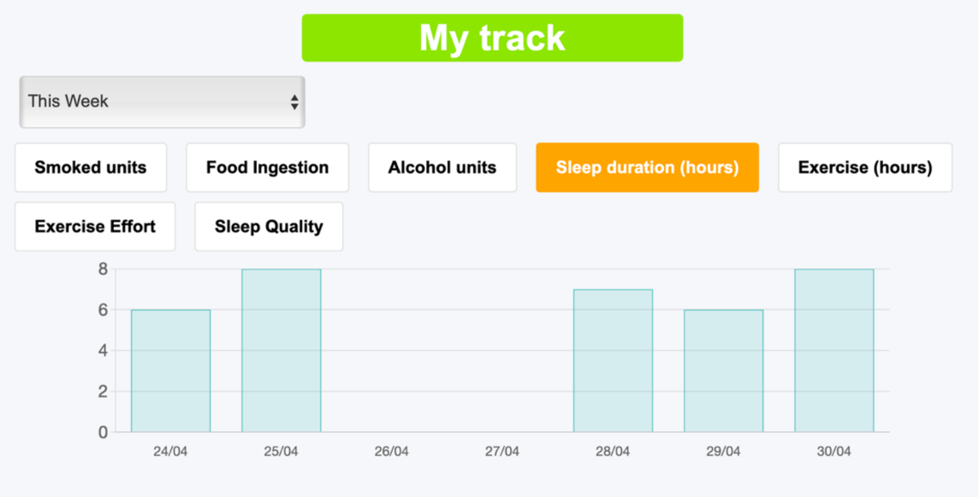

Figure 65. 'My track' page on the wellbeing monitoring website

*Note: Every time you open your website in Webflow (Figure 66), it is important to make sure you are in **'Design' mode**, to be able to modify the website.*

- *To check it, look if **'Design'** appears in the top-left corner of the screen (Figure 67).*
- *If it doesn't, click there and switch to **'Design' mode**.*

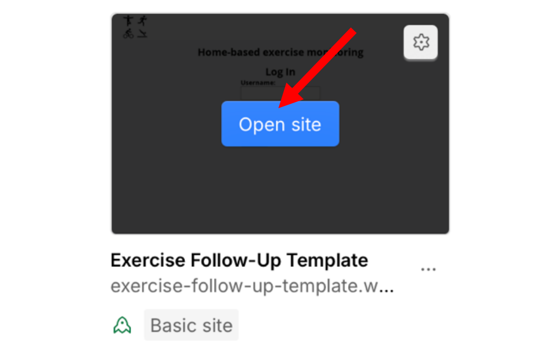

Figure 66. Opening the website

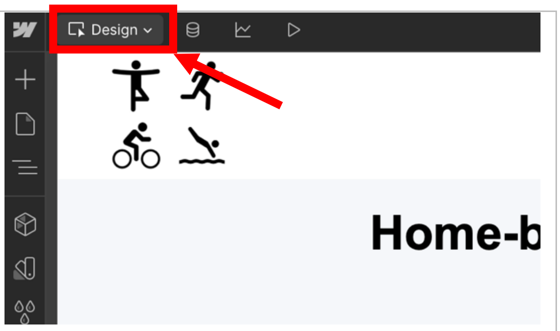

Figure 67. Verifying that you are in Design mode

1. *Changing the website's logo*

1. **Choose the logo image** you would like to use and save it in your computer.
2. Next, follow all the steps in section ***1. Changing the website's logo*** in the **Annex**.
3. Once you have completed the steps, you will have successfully updated the logo of the website with your chosen image.

2. *Changing the form in the 'Enter data' page*

1. In the left menu in Webflow, click the **'Pages'** icon.
2. Click the ***Patients – Enter data*** page.
3. Let's adjust the Yes/No options fields:
   - Hover over the **yellow container** that contains the **'Cool down'** question until you see **'Container Yes_No'** (Step 1 – Figure 68), then **click** to select it.
   - **Right-click** the container and select ***Delete*** (Step 2 – Figure 68).

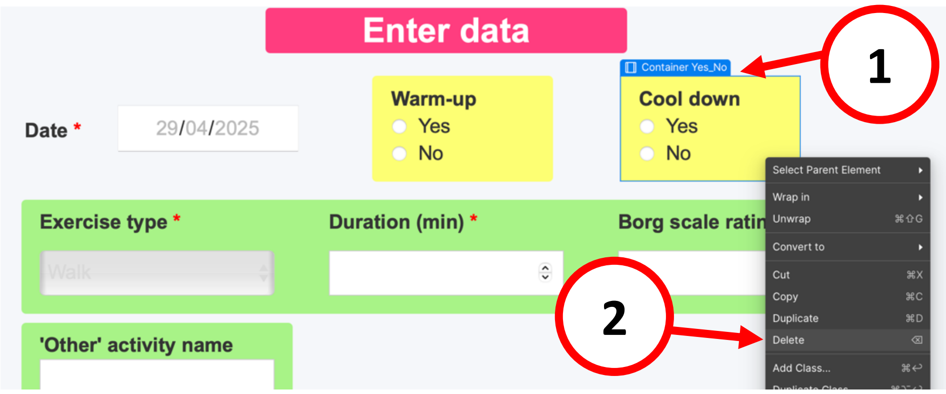

Figure 68. Deleting the 'Cool Down' question

- Now, **double-click** the **‘Warm-up’ label** (Figure 69).
    - Delete the existing text and replace it with **‘Medication taken’** (Figure 70).

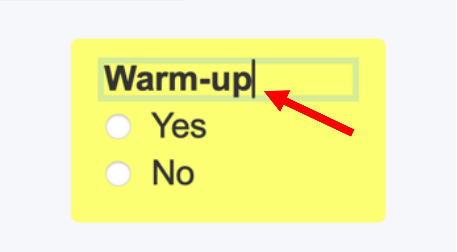

Figure 69. Modifying the ‘Warm-Up’ question

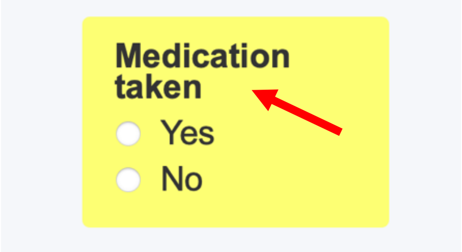

Figure 70. Replacing the label of the ‘Warm-Up’ question

4. Next, on the green container with three columns:
    - Click it to select it.
    - In the right panel, click ***Style*** (Figure 71).
    - Then, change the ‘Layout’ to ***Block*** (Figure 71). We are only changing this to make it easier to edit.

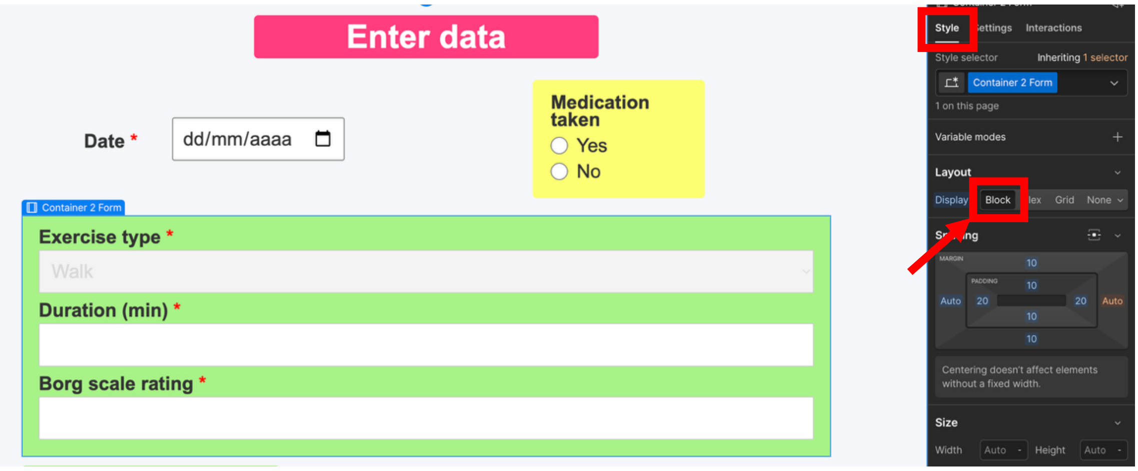

Figure 71. Changing the Layout of the green container

- Now, **double-click** the **‘Exercise type’ label**.
    - Delete the existing text (but **leave the red asterisk**) and replace it with: ***Question***

- **Double-click** the **‘Duration (min)’ label**.
    - Delete the existing text (but **leave the red asterisk**) and replace it with: ***Value***

- **Double-click** the **‘Other activity name’ label**.
    - Delete the existing text and replace it with: ***Additional comments***

The first part of the form should now look like Figure 72.

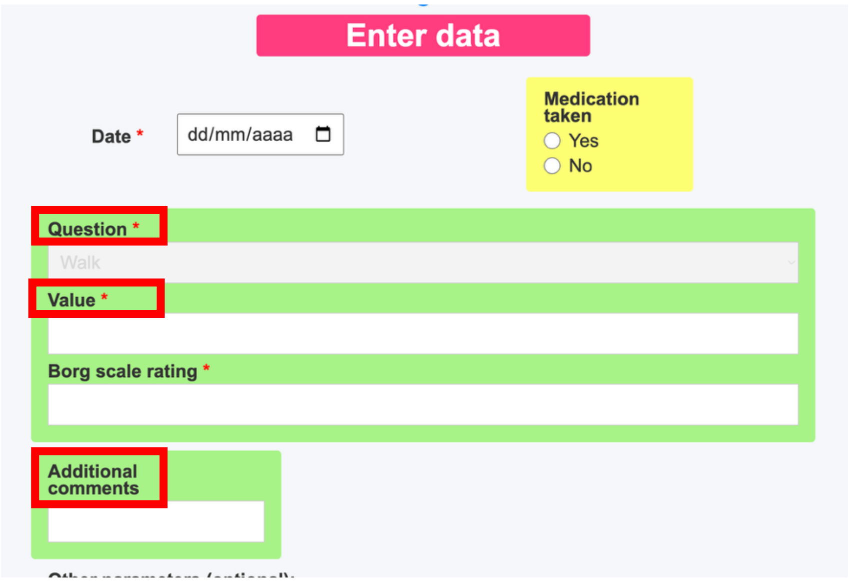

Figure 72. First part of the form with modifications

- Click the **field under 'Borg scale rating'** (Figure 73).
  - **Right-click** it and select ***Delete***.

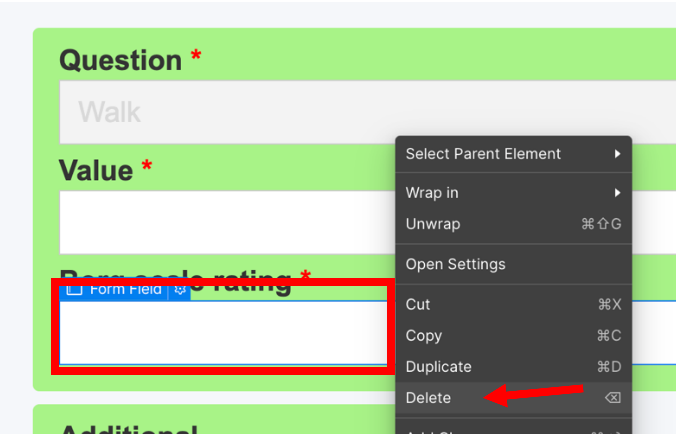

Figure 73. Deleting the 'Borg scale rating' label

- Click the **label 'Borg scale rating'**
  - **Right-click** it and select ***Delete*** (Figure 74).

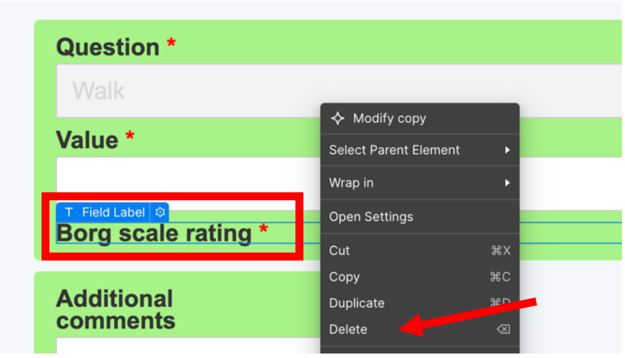

Figure 74. Deleting the 'Borg scale rating' label

5. Now, hover over the same **green container** until you see **‘Container 2 Form’** (Step 1 – Figure 75), then **click** to select it.

- In the right panel, click ***Style*** (Step 2 – Figure 75).
- In ‘Display’, under *Layout*, click ***Grid*** (Step 3 – Figure 75).
- Then, next to *Grid*: update the value for **‘Columns’** to **2** (Step 4 – Figure 75), then press Enter to apply the change.

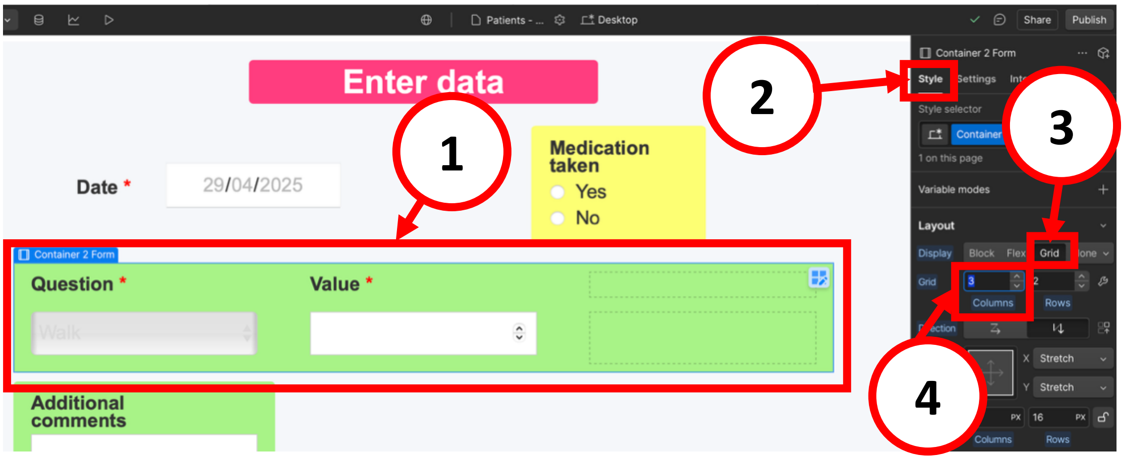

Figure 75. Changing the layout of ‘Container 2 Form’ to two columns

- Now, the container form should look like Figure 76.

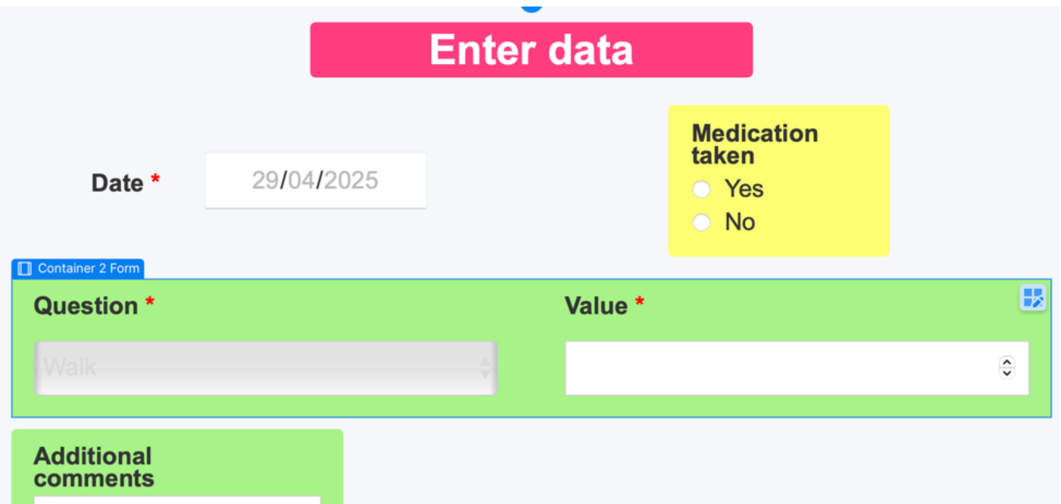

Figure 76. New layout of ‘Container 2 Form’

6. Well done! Now, hover over the **green 'Additional comments' container** until you see **'Other_activity Container'** (Step 1 – Figure 77), then **click** to select it.

   - In the right panel, make sure you are on the ***Style*** tab (Step 2 – Figure 77).

   - Under *Size*, update the value next to *Width* to **320** (Step 3 – Figure 77), then press Enter to apply the change.

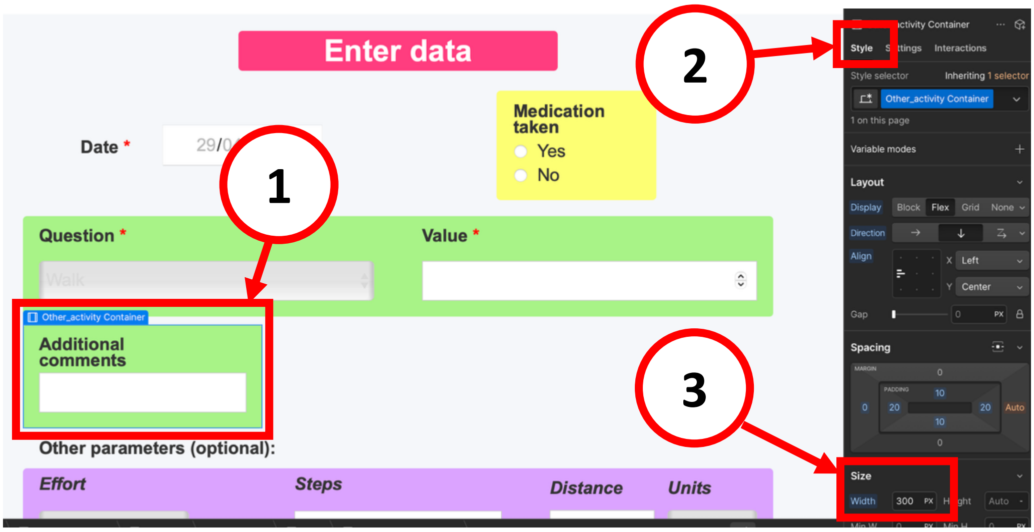

Figure 77. Updating the 'Width' value of the 'Additional comments' container

   - Perfect! We have just made the container bigger, so it fits the new label better.

7. Now**, double-click** the **field under 'Question'** (Step 1 – Figure 78).
   - The options of the dropdown will appear.

   - **Delete all the current options** by clicking the **trash icon** (Step 2 – Figure 78).

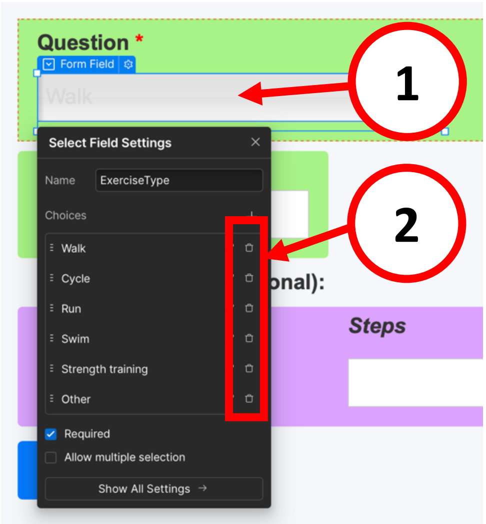

Figure 78. Deleting the dropdown options

- Now, click the **‘+’** icon to add a new option (Figure 79):
    - Update the **‘Text’** field with: ***Select one...***
    - Leave the **‘Value’** field empty.

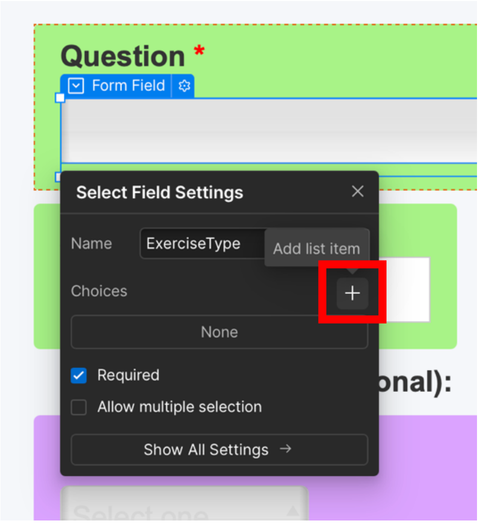

Figure 79. Adding a dropdown option

- Next, let’s add the remaining options for the dropdown.
  Note: If you don’t want to track some of the following options, you can skip them.

- Click the **‘+’** icon again to add another option:
    - Update the **‘Text’** field with: ***Sleep duration (hours)***
    - Update the **‘Value’** field with: ***Sleep duration (hours)***

- Click the **‘+’** icon to add another option:
    - Update the **‘Text’** field with: ***Sleep Quality***
    - Update the **‘Value’** field with: ***Sleep Quality***

- Click the **‘+’** icon to add another option:
    - Update the **‘Text’** field with: ***Exercise (in hours)***
    - Update the **‘Value’** field with: ***Exercise (hours)***

- Click the **‘+’** icon to add another option:
    - Update the **‘Text’** field with: ***Exercise Effort***
    - Update the **‘Value’** field with: ***Exercise Effort***

- Click the **‘+’** icon to add another option:
    - Update the **‘Text’** field with: ***Smoked units***
    - Update the **‘Value’** field with: ***Smoked units***

- Click the **‘+’** icon to add another option:
    - Update the **‘Text’** field with: ***Alcohol units***
    - Update the **‘Value’** field with: ***Alcohol units***

- Click the **‘+’** icon to add another option:
    - Update the **‘Text’** field with: ***Food Ingestion***
    - Update the **‘Value’** field with: ***Food Ingestion***

The dropdown options should now look similar to those in Figure 80.

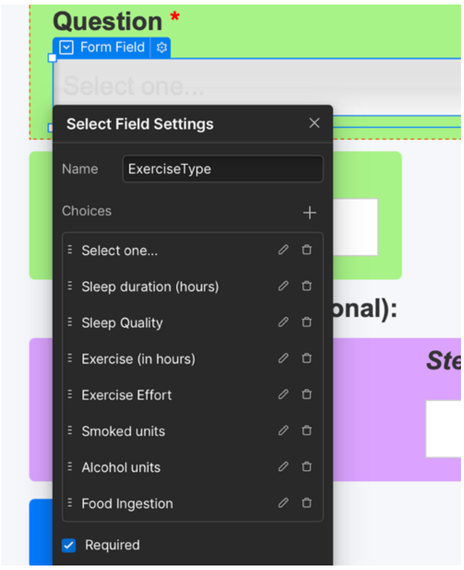

Figure 80. ‘Question’ dropdown with new options

8. Perfect! Now, let’s delete some of the questions of the **purple container**:
- Hover over the **‘Steps’ question** until you see **‘Container Optional Field’** (Figure 81).
    - Then, **click** to select it.
    - **Right-click** it and select ***Delete***.

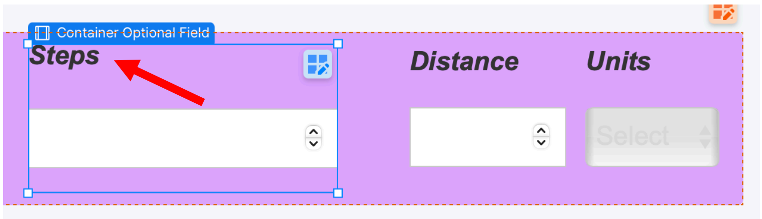

Figure 81. Selecting the ‘Container Optional Field’

- Then, hover over the **‘Distance’ and ‘Units’ questions** until you see **‘Container Distance’** (Figure 82).
    - Then, **click** to select it.
    - **Right-click** it and select ***Delete***.

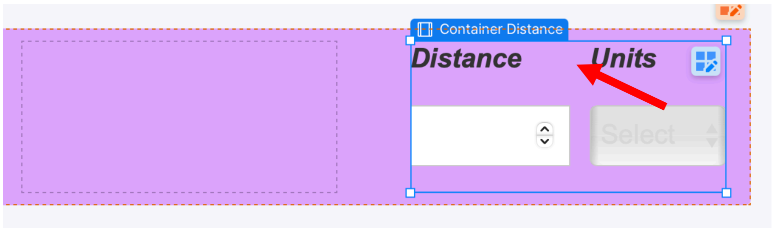

Figure 82. Selecting the ‘Container Optional Field’

- Now, hover over the **purple container** until you see **‘Container Other Parameters’** (Step 1 - Figure 83).
    - Click to select it.

    - In the right panel, make sure you are on the ***Style*** tab (Step 2 – Figure 83).

    - Under *Layout*, next to *Grid*: update the value for **‘Columns’** to **1** (Step 3 – Figure 83), then press Enter to apply the change.

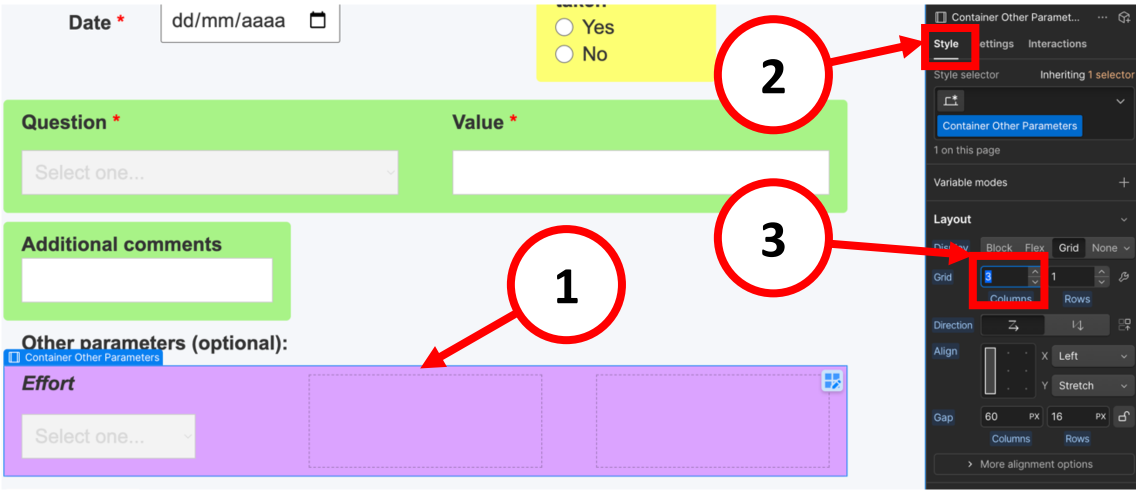

Figure 83. Modifying the ‘Container Other Parameters’

- Now, scroll down in the right panel:
    - In *Spacing*, click on the ‘Auto’ under *Margin* and select **0** (Step 1 - Figure 84).

    - Under *Size*, update the value next to *Width* to **320** (Step 2 - Figure 84)

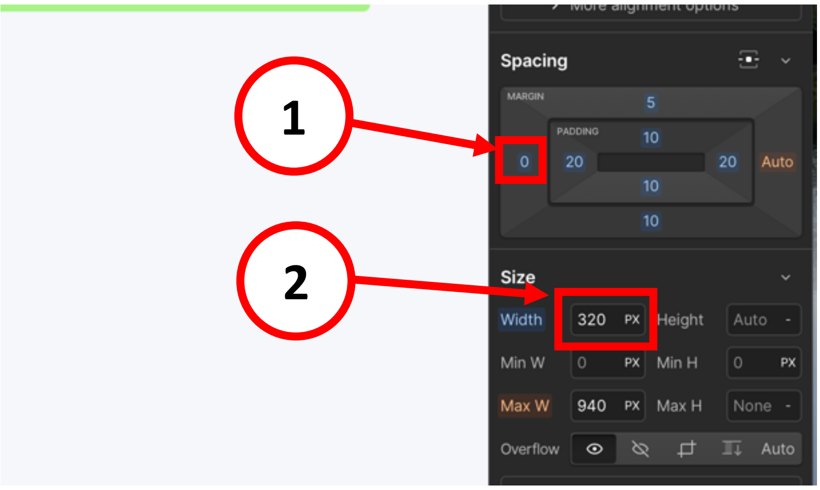

Figure 84. Modifying the ‘Container Other Parameters’

9. We are almost done! Now, let's change the 'Effort' question to 'Wellbeing perception'. To do so, follow the next steps:
   - Hover over the **'Effort' question** until you see **'Container Optional Field'** (Figure 85).
     - **Click** to select it.
     - In the right panel, make sure you are on the ***Style*** tab.
     - Then, under *Layout*, change the *Display* to ***Block*** (Figure 86).

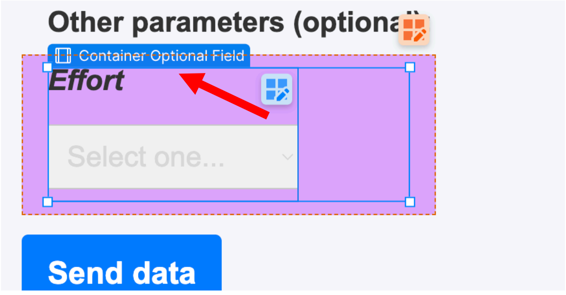

Figure 85. Selecting the 'Container Optional Field'

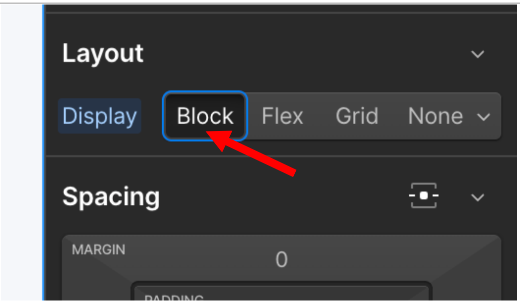

Figure 86. Changing the Display to 'Block'

   - Now, **double-click** the **'Effort' label** (Figure 87).
     - Delete the existing text and replace it with **'Wellbeing perception'** (Figure 80).

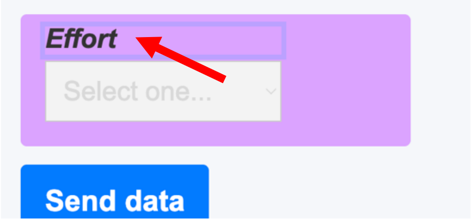

Figure 87. Modifying the 'Effort' label

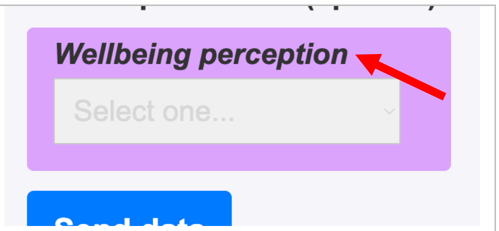

Figure 88. Replacing the label of the 'Effort' question

   - **Double-click** the **field under 'Wellbeing perception'** to modify the dropdown options:
     - **Delete all the current dropdown options (except the 'Select one…' option)** by clicking the **trash icon**.

     - Click the **'+'** icon to add a new option:
       - Update the **'Text'** field with: **'Poor'**
       - Update the **'Value'** field with: **'Poor'**

     - Click the **'+'** icon to add another option:
       - Update the **'Text'** field with: **'Medium'**
       - Update the **'Value'** field with: **'Medium'**

     - Click the **'+'** icon to add another option:
       - Update the **'Text'** field with: **'Good'**
       - Update the **'Value'** field with: **'Good'**

The dropdown options **under 'Wellbeing perception'** should now look like in Figure 89.

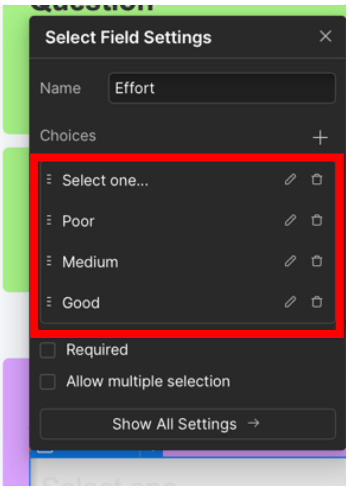

Figure 89. New 'Wellbeing perception' dropdown options

- Close the *Select Field Settings* panel by **clicking the 'X'**.

10. In the left menu, click the **'+' icon** (Step 1 – Figure 90).
    - Search for the element ***Block Quote*** (Step 2 – Figure 90).
    - Drag the element ***Block Quote*** into the form and place it between the two green containers (Figure 91).

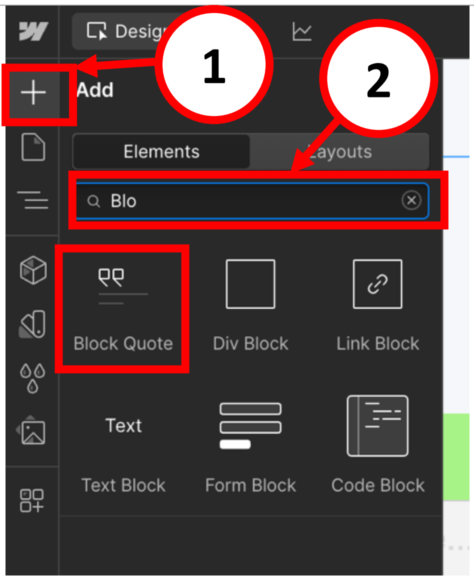

Figure 90. Adding the element 'Block Quote'

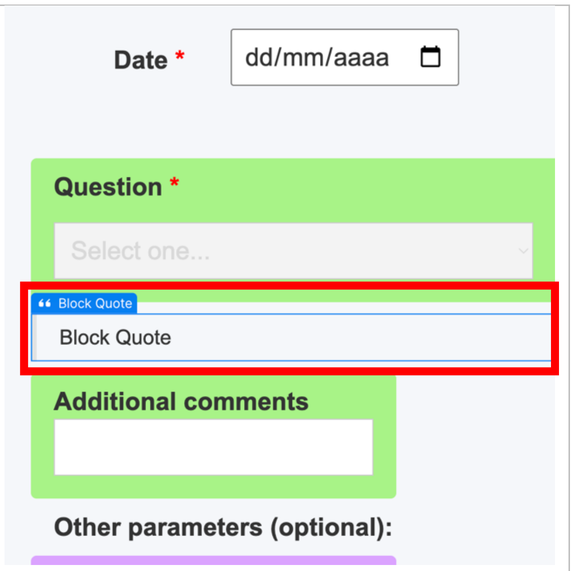

Figure 91. Placing the 'Block Quote' element

- **Double-click** the *Block Quote* in the form to edit it. Replace the text with the following (you can copy and paste it, and then adjust the spacing if needed):
  For **Sleep Quality** values: Bad = **1** / Medium = **2** / Good = **3**
  For **Effort** values: Low = **1** / Medium = **2** / High = **3**
  For **Food ingestion** values: Low = **1** / Medium = **2** / High = **3**

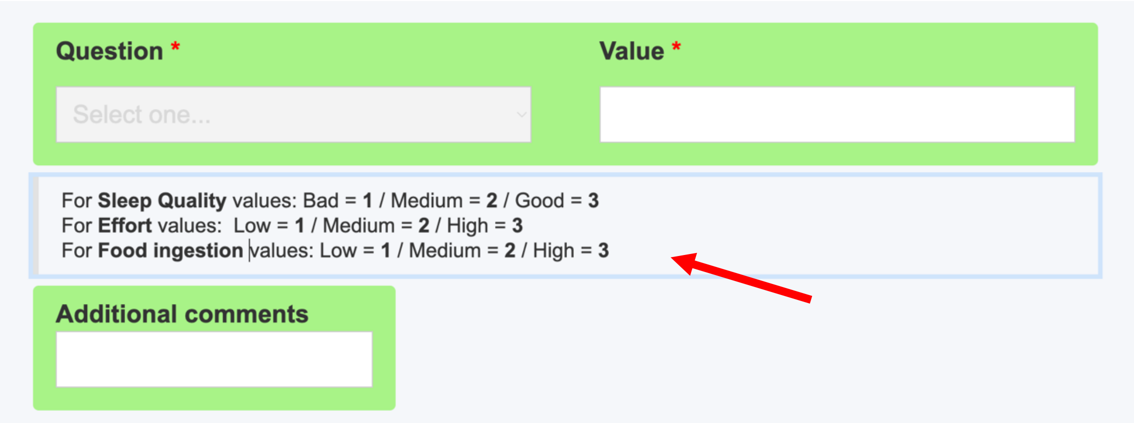

Figure 92. Form with the final 'Block Quote' element

11. **Congrats!** The form is now adapted.
    Next, follow the steps in ***3. Adapting the graphs*** section to update the corresponding graph views.

3. *Adapting the graphs*

1. In the left menu, click on the **'Pages'** icon.
2. Click the ***Patients – My track*** page.
3. Follow **Steps 3 to 5 from section *5.3. Deleting a filter*** **twice**:
    - First to **delete the *'Kilometers (Km)'* filter** (Figure 93).
    - And then to **delete the *'Select Borg Scale Range'* filter** (Figure 93).

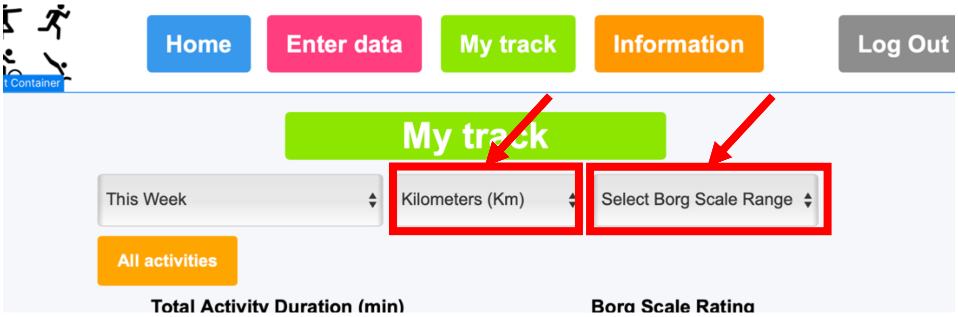

Figure 93. Filters to delete in the *Patients – My track* page.

4. Click the **'All activities'** button to select it (Figure 94).
    - **Right-click** it and select ***Delete***.

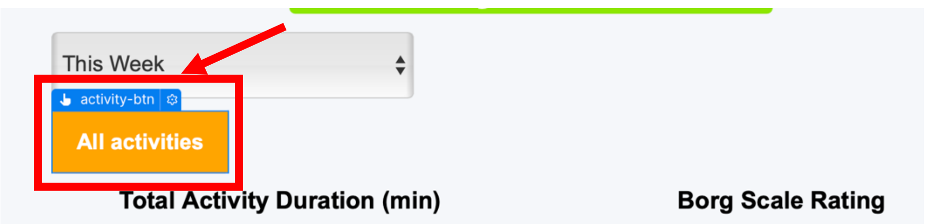

Figure 94. Deleting the 'All activities' button

5. Now, hover over the **'Borg Scale Rating'** graph area until you see **'Container Graphs Grid'** (Figure 95).

   - **Click** to select it.
   - **Right-click** it and select ***Delete***.

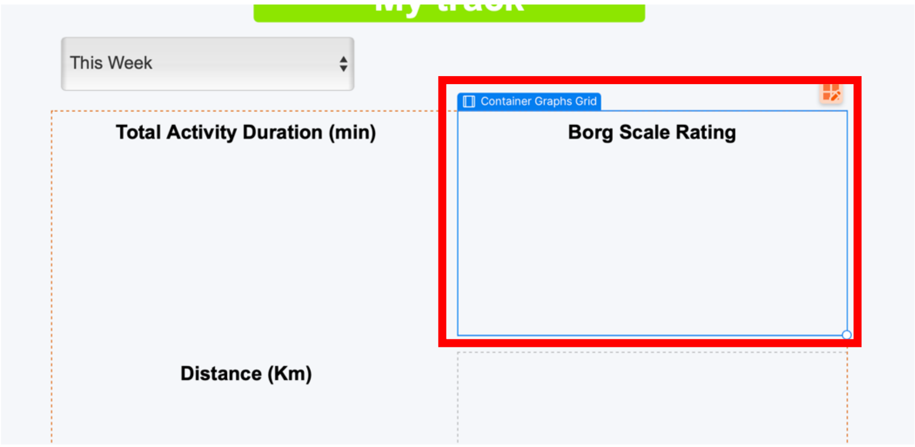

Figure 95. Deleting the 'Container Graphs Grid' of 'Borg Scale Rating'

6. Repeat the previous step (Step 5) for the **'Distance (Km)'** graph area (Figure 96).

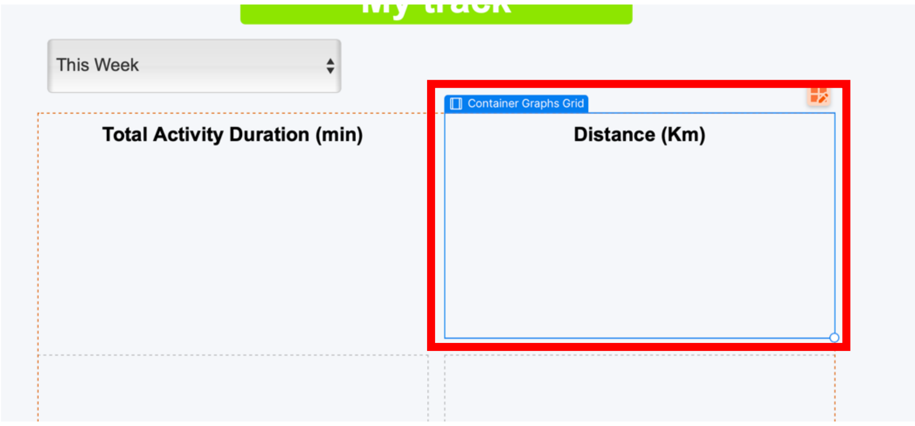

Figure 96. Deleting the 'Container Graphs Grid' of 'Distance (Km)'

7. Perfect! Now we have the graph we want on the screen, but we need to adjust its position to center it.
   - To do this, hover over the area until you see **'Container Grid'** (see Step 1 - Figure 97) and **click** to select it.

   - In the right panel, click ***Style*** (Step 2 - Figure 97).

   - Under *Layout*, next to *Grid*: set **'Columns'** to **1** and **'Rows'** to **1** (Step 3 – Figure 97). In Figure 97, both values are currently 2 – these are the values you need to change to 1.

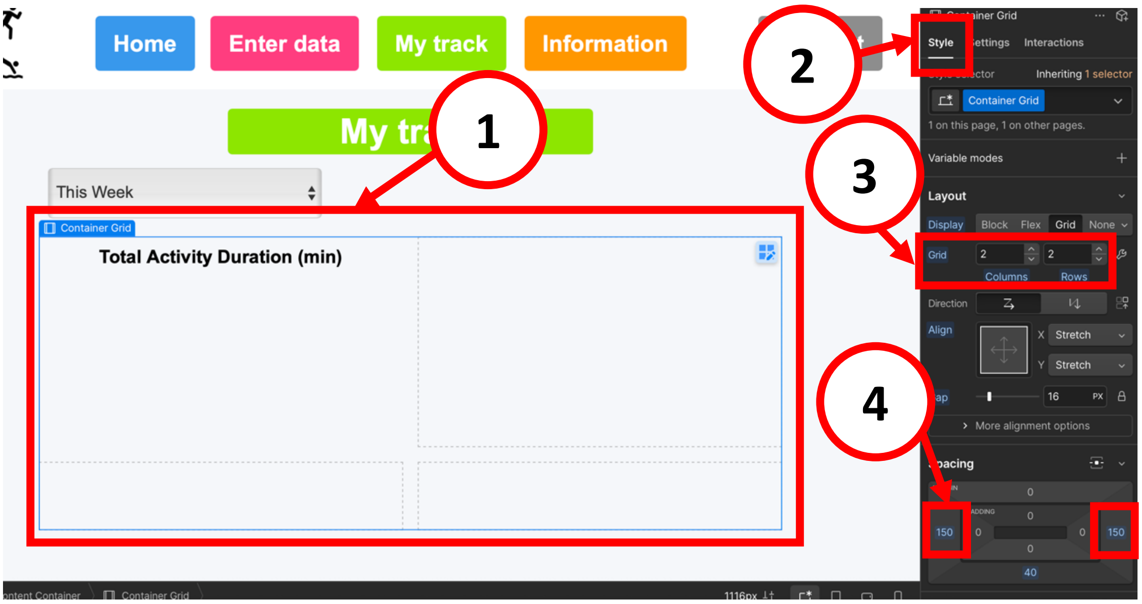

Figure 97. Modifying 'Container Grid'

   - Then, under *Spacing*, click the **left margin field** (Step 4 – Figure 97), set it to **240** (Figure 98) and press Enter to apply the change.
     Do the same for the **right margin field**.

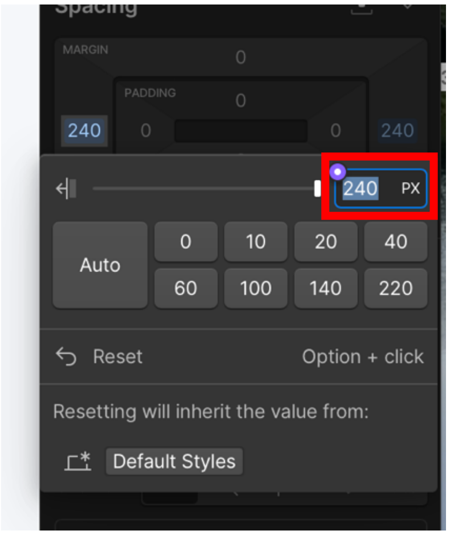

Figure 98. Setting the left and right margins to 240 px

8. Next, click on the **'Total Activity Duration (min)' title**.
   - **Right-click** it and select ***Delete*** (Figure 99).

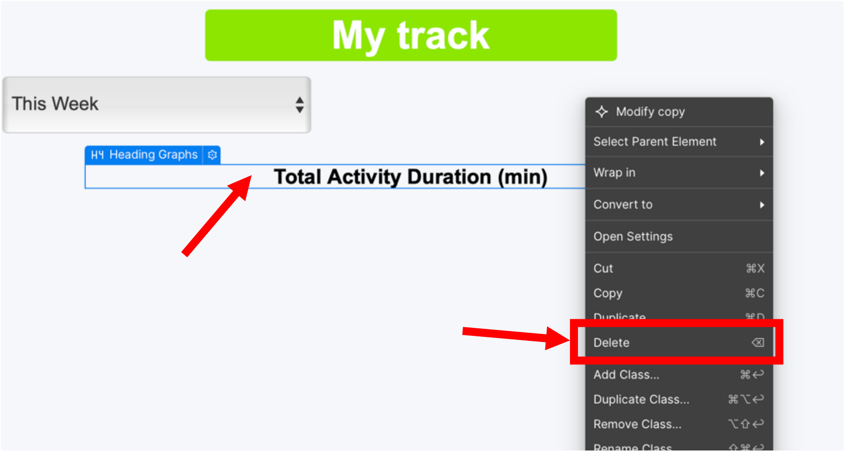

Figure 99. Deleting the 'Total Activity Duration (min)' title

9. Perfect. Now that we have modified the graphs for the desktop view of the website, let's move on to adjusting the graphics for the phone version.

- On the **top menu**, click **'Desktop'**. Then, click **'Mobile (L)'** to switch the display view (Figure 100).

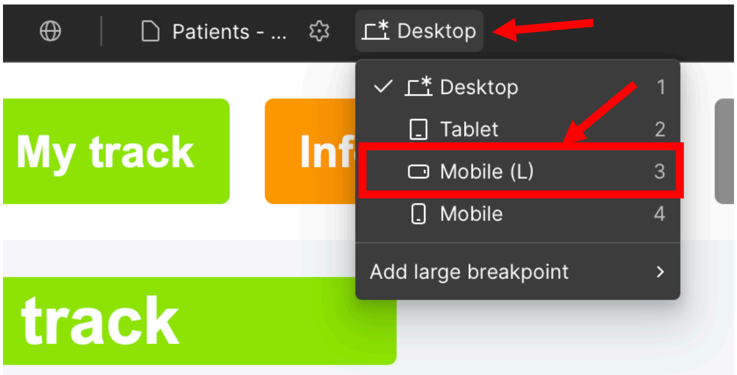

Figure 100. Switching the display view to 'Mobile (L)'

10. Next, click on the **Navigator icon** in the left-menu **(Step 1 – Figure 101)**.

    *Note: The Navigator contains the structure of the different elements that appear on the page. The **arrow icons (>)** are used to view the different components inside each element.*

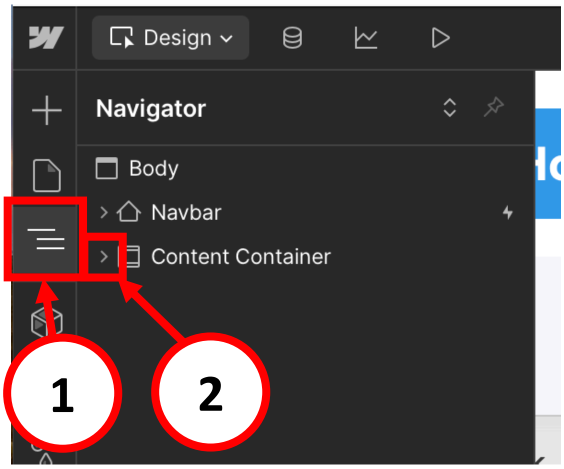

Figure 101. Opening the Navigator menu

11. Click the **arrow icon (>)** next to **'Content Container'** (Step 2 - Figure 101), if it is not already open.

12. Click the **arrow icon (>)** next to **'Slider'** in the Navigator (Step 1 – Figure 102), if it is not already open.

- Then, click the **arrow icon (>)** next to **'Mask'** (Step 2 – Figure 102).

- Right-click ***Slide 3*** and select ***Delete*** (Step 3 – Figure 102).

- Right-click ***Slide 2*** and select ***Delete***.

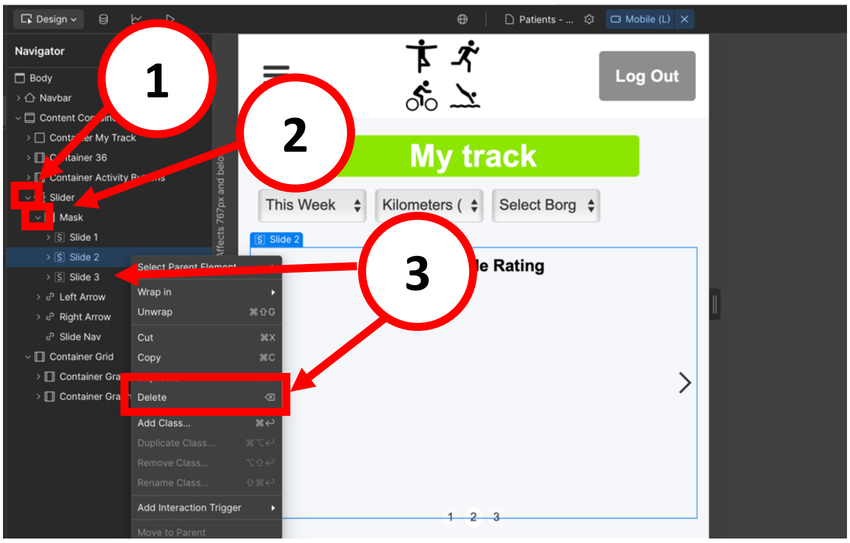

Figure 102. Deleting graphs in mobile display view

- Click the **arrow icon (>)** next to **'Slide 1'** (Step 1 – Figure 103).

- Right-click on **'Heading Graphs'** and select ***Delete*** (Step 2 – Figure 103).

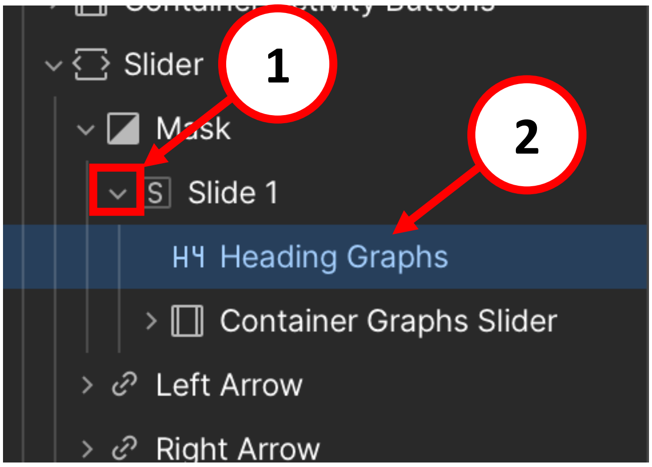

Figure 103. Deleting 'Heading Graphs' in 'Slide 1'

- Perfect! You have successfully adapted the graphs for both the desktop and mobile views. **Click the 'X' next to 'Mobile (L)'** at the top of the screen to exit the mobile view.

13. Great! Now, in the left menu, click on the **'Pages'** icon.

14. Click the ***Staff – My patients*** page.

15. Repeat Steps 3 to 6 and 8 to 12 for this page (Skip Step 7).

16. Publish the website to apply and save your changes (For detailed instructions, refer to section *4. Publishing the Website*).

17. Great. Now, just a few more steps to complete! Go to **Xano**.
Let's update the database to align with the form.

18. Click on ***Database*** in the left menu.
Note: If you do not find 'Database' because you have just logged into Xano, click on 'Free Instance' to get to where we are.

19. Then, click on the **'data_entries'** table.

20. Right-click the column name **'WarmUp'**.
- Then, select ***Rename*** (Figure 104).
- Rename it to **'MedicationTaken'** (Figure 105).
- Click ***Save***.
- A confirmation banner will appear, click ***Confirm***.
- A panel in the right side of the screen will appear, click ***Update references*** (Figure 106).

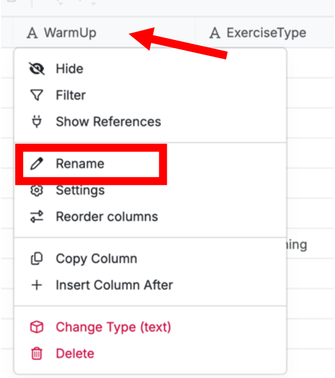

Figure 104. Renaming a column in Xano

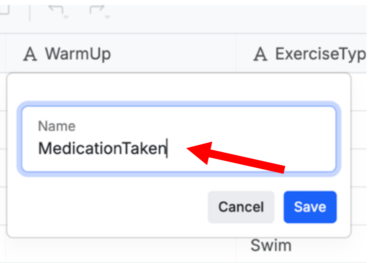

Figure 105. Renaming 'WarmUp' column to 'MedicationTaken'

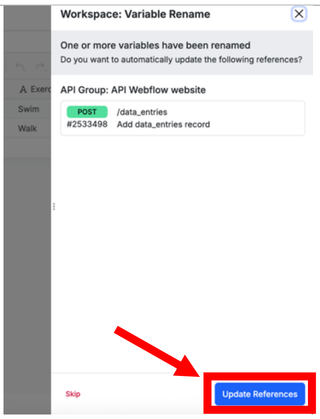

Figure 106. Clicking 'Update References'

21. Scroll to the right and **right-click** the column name **'Other_activity'**.
- Then, select ***Rename*** and rename it to **'Comments'**.
- Click ***Save***.
- A confirmation banner will appear, click ***Confirm***.
- A panel in the right side of the screen will appear, click ***Update references***.
- Click at the **'⇄' next to the name of the column 'Comments'**.
- Reorder the column 'Comments' by dragging it under 'Duration'.
- Click ***Save***.

22. Right-click the column name **'BorgScale'**.
- Then, select ***Delete***.
- A confirmation banner will appear, click ***Delete*** again.

23. Right-click the column name **'Steps'**.
- Then, select ***Delete***.
- A confirmation banner will appear, click ***Delete*** again.

24. Right-click the column name **'Effort'**.
- Then, select ***Rename***.
- Rename it to **'Wellbeing'**.
- Click ***Save***.
- A confirmation banner will appear, click ***Confirm***.
- A panel in the right side of the screen will appear, click ***Update references***.

- **Right-click** the new column name **'Wellbeing'**.
- Then, select ***Change Type (integer)*** (Figure 107).
- Under *Type* select **'text'** (Figure 108).
- Click ***Save***.

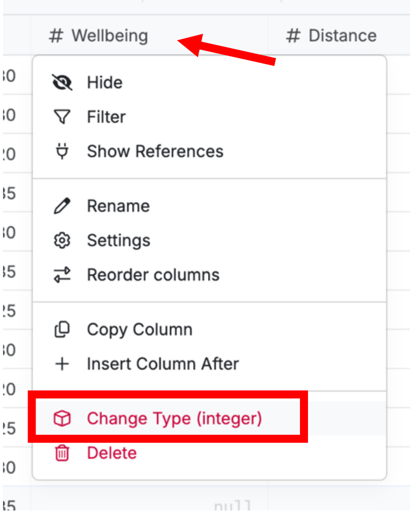

Figure 107. Clicking 'Change Type (integer)' in 'Wellbeing' column

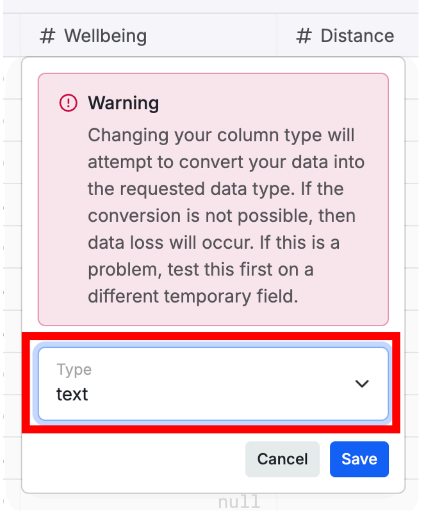

Figure 108. Changing Type to 'text' in 'Wellbeing' column

25. Right-click the column name **'Distance'**.
- Then, select ***Delete***.
- A confirmation banner will appear, click ***Delete*** again.

26. Right-click the column name **'Units'**.
- Then, select ***Delete***.
- A confirmation banner will appear, click ***Delete*** again.

27. Right-click the column name **'CoolDown'**.
- Then, select ***Delete***.
- A confirmation banner will appear, click ***Delete*** again.

28. Now, **check the box next to '# id'** (Step 1 – Figure 109) to select all the entries.
- Then, click ***Delete*** (Step 2 – Figure 109)
- A confirmation banner will appear, click ***Confirm***.

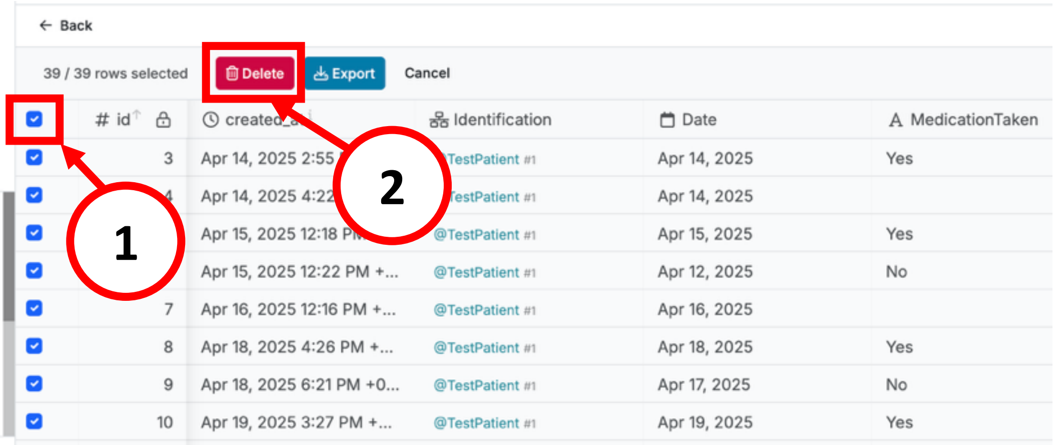

Figure 109. Deleting all existing entries

29. Great! Now, while still in Xano:
- Click on **API** in the left menu (Step 1 – Figure 110).

- Then, click on the folder named **'API Webflow website'** (Step 2 - Figure 110).

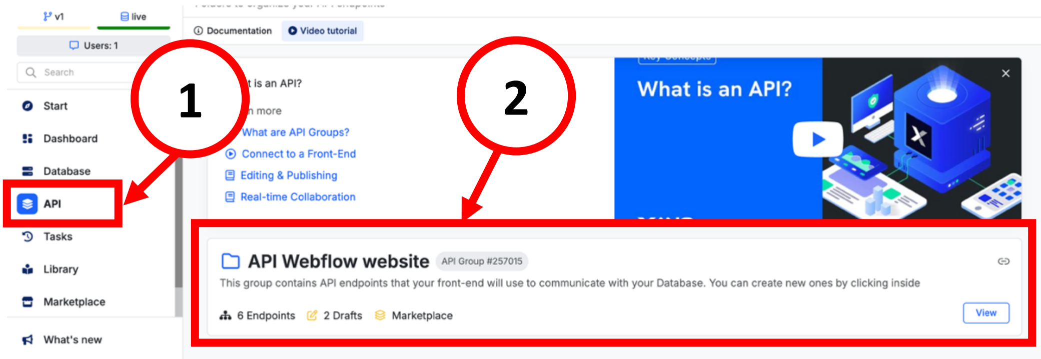

Figure 110. Accessing the 'API Webflow website' folder in Xano

- Inside the folder, there is a section named **'data_entries'**. In that section, you will see **POST data_entries** marked as ***'Draft'*** (Figure 111).

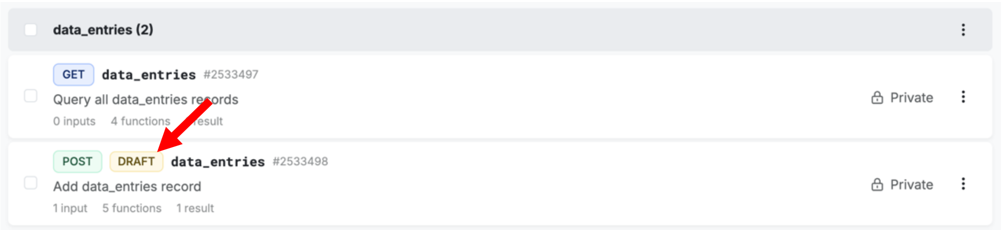

Figure 111. Section 'data_entries' inside the 'API Webflow website' folder in Xano

30. Click on **POST data_entries**

31. Then, click the **'Publish'** button **(Figure 112).**

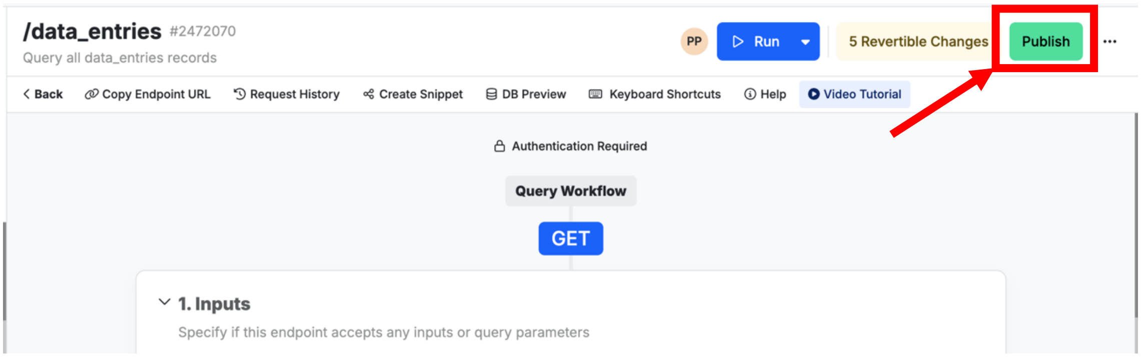

Figure 112. 'Publish' button inside a subsection

32. A tab will appear in the right side of the screen.
- Click the ***Publish*** button again (See Figure 113).

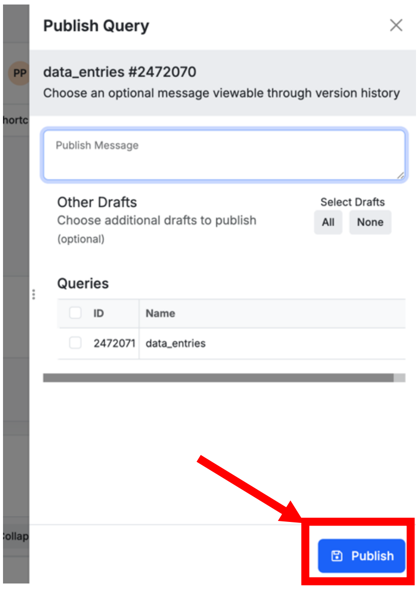

Figure 113. 'Publish' button in the new tab

33. Return to the previous screen.

34. **Congratulations!** You have successfully adapted the graphs and the corresponding database structure.
Now you can go to the published website and try sending information as a patient, and viewing it as a patient and as a staff user, to make sure everything is working as expected.

4. *Managing information documents*

1. The website has two 'Information' pages:
   - One that only patients can see: ***Patients – Information*** page
   - And one that only staff members can see: ***Staff – Information*** page

2. To view the information documents available for patients, log into the published website as a patient and click on the documents on the *Information* page.
   You can do the same with a test staff user to view their documents.

3. Now, follow the steps in steps in section ***How to delete a document, image, link or video*** to delete an information document.

4. Perfect! Now, let's add a new one. **Choose an information document, image, link or video** you would like to add to the website.

5. **Decide** whether you want to add the document to the patient information page, the staff information page, or both.

6. If you want to add it to:

   - The patient information page:
     - Follow the steps in section ***How to add a document, image, link or video*** and, in Step 2, click ***Patients – Information*** page.

   - The staff information page:
     - Follow the steps in section ***How to add a document, image, link or video*** and, in Step 2, click ***Staff – Information*** page.

   - Both pages:
     - Follow the steps in section ***How to add a document, image, link or video*** **twice**:
       - First time: In Step 2, click ***Patients – Information*** page.
       - Second time: In Step 2, click ***Staff – Information*** page.

Once you have followed these sections, you will have successfully converted the website into a patient behaviour and wellbeing monitoring website.

Additionally, if you would like to change the title of the website in the Log In page, you can:

- In Webflow, click the **'Pages' icon**.
- Click ***Log In Page***
- **Double-click** the title 'Home-based exercise monitoring' and edit it to the title you prefer.

Annex A: How to Modify the Website

*Note: To make the modifications in the website (in Webflow) that are explained in this Annex, it is important to make sure you are in **'Design' mode** (Figure A.1).*

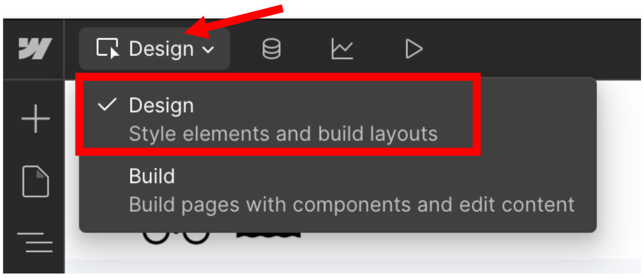

Figure A.1. Verifying that 'Design' mode is enabled in Webflow

1. Changing the website's logo (level of difficulty: **1**/5)

1. In Webflow, click ***Open site*** to start editing your website (if you are not already doing so).

2. Access ***Assets*** from the left-menu (Step 1 – Figure A.2)

3. Click the **'Upload' icon** (Step 2 – Figure A.2)

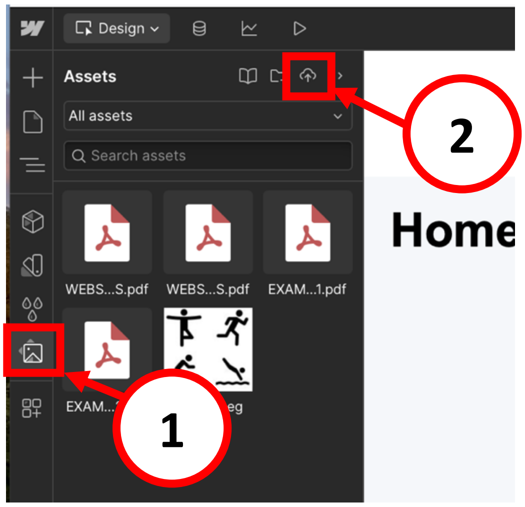

Figure A.2. Uploading 'Assets' in Webflow

4. **Select the image** from your computer that you would like to use as logo **and upload it**.

5. Click the ***Assets*** icon again to close the panel.

6. Now, we will replace the existing logo on the page with the new image. This change will automatically be applied across all the pages of the website.

6.1. **Double-click** the **current logo image** until it is outlined in blue (Step 1 – Figure A.3).

6.2. Click the **' ⚙ ' icon** next to *Image* (Step 2 – Figure A.3).

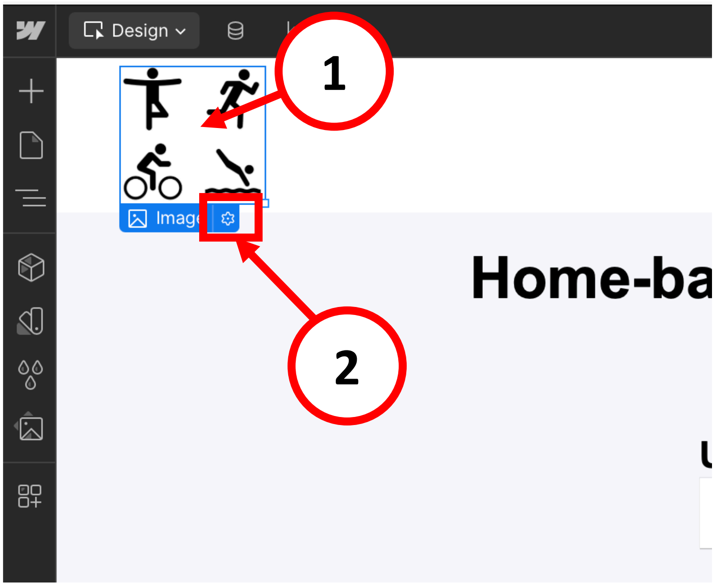

Figure A.3. Opening the logo's image settings

6.3. Click on ***Replace Image*** (Figure A.4).

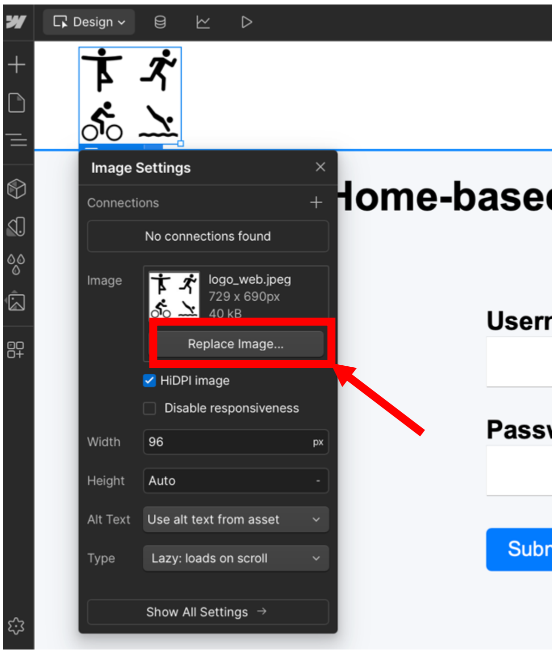

Figure A.4. *Replace Image* location in Image Settings

6.4. **Select the image** you uploaded earlier from the asset library.

6.5. Close the Image Settings panel by clicking the 'X'.

6.6. **Adjust the image size** by dragging the bottom-right corner until it reaches the desired dimensions (Figure A.5).

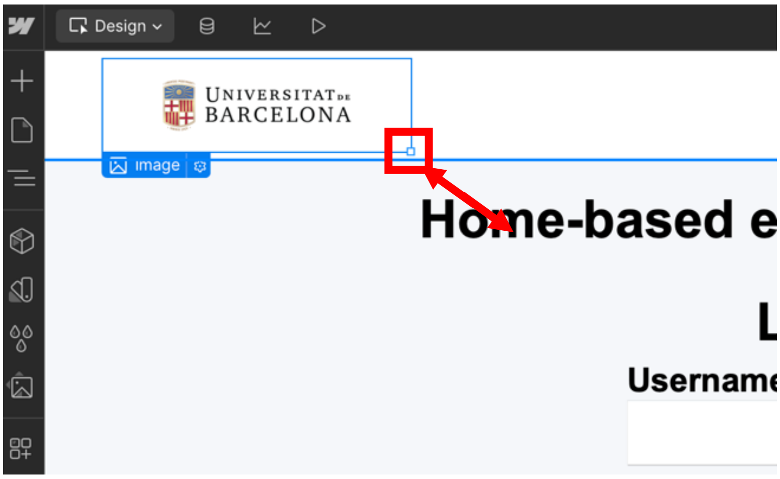

Figure A.5. Adjusting the logo image size using the bottom-right corner.

7. Double-click anywhere on the page to stop selecting the image.

8. **Publish the website** to apply and save your changes (For detailed instructions, refer to section *4. Publishing the Website*).

9. Congrats! You have successfully changed the website's logo.

Note:

- *If you arrived here while following Example 1, click here to return to Example 1.*
- *If you arrived here while following Example 2, click here to return to Example 2.*

2. Modifying the 'Information' pages (level of difficulty: **1**/5)

The website has two 'Information' pages:

- One that only patients can see: ***Patients – Information*** page
- And one that only staff members can see: ***Staff – Information*** page

Thus, each Information page can contain different documents.

This section shows *How to add a document, image, link or video* and *How to delete a document, image, link or video*.

2.1. How to add a document, image, link or video

1. Open your site in Webflow. Click on **'Pages'** in the left menu (Step 1 – Figure A.6)**.**

2. If you: (Step 2 – Figure A.6)

- Want to **update the patients' information documents**:
  → click on ***Patients – Information*** page

- Want to **update the staff's information documents**:
  → click on ***Staff – Information*** page

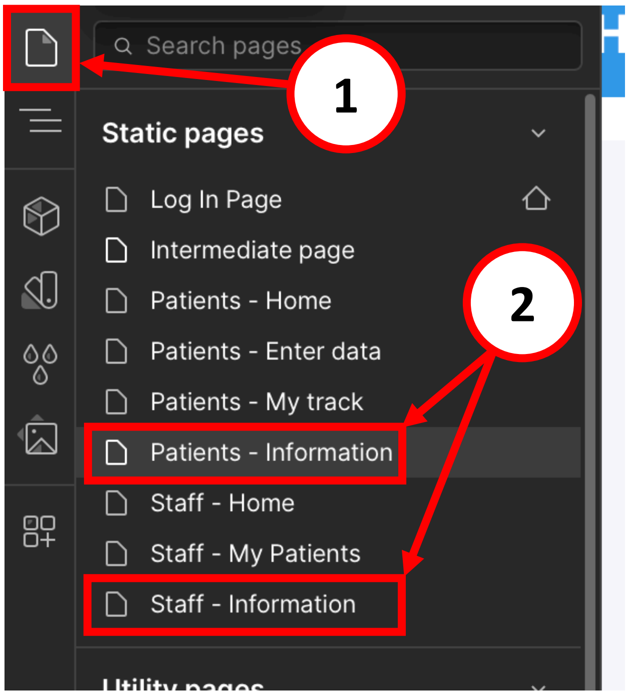

Figure A.6. Locating the Patients' and Staff's Information pages in the 'Pages' menu

3. Hover your mouse over the screen until you see **'Container Info' (Step 1 – Figure A.7)**, then **click to select it**.

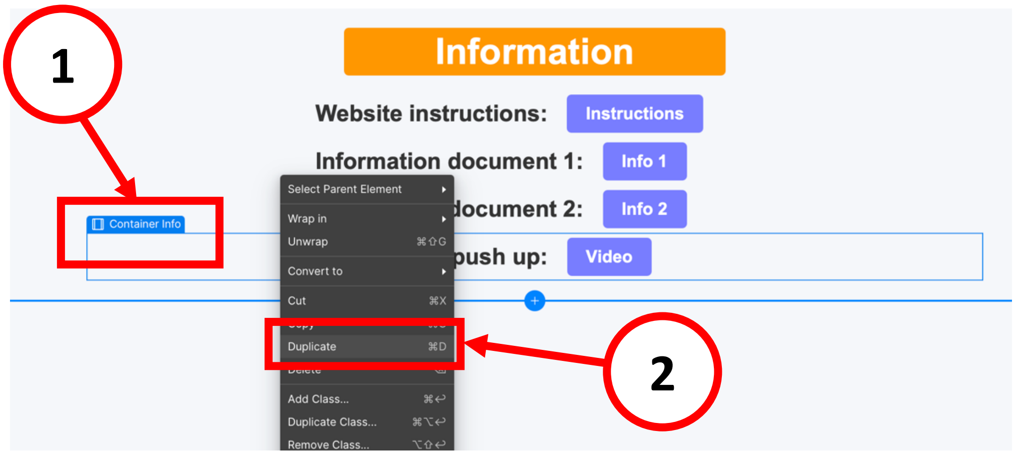

Figure A.7. Duplicating the 'Container Info'

4. **Right-click** on the selected container.

5. **Select 'Duplicate' (Step 2 – Figure A.7)**.
This will create a copy of the container, which includes a title and a button (linked to the document or link). We are going to edit this duplicate to add our new document, link or video.

6. In the duplicate container:
- Double-click the **title** to edit it (Figure A.8)
- Double-click the **button** to change its name (Figure A.9).

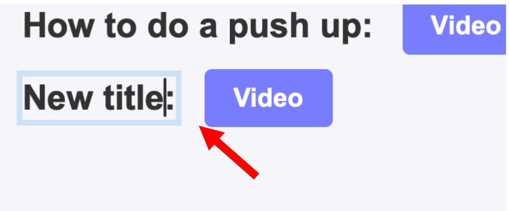

Figure A.8. Editing the title of the information document

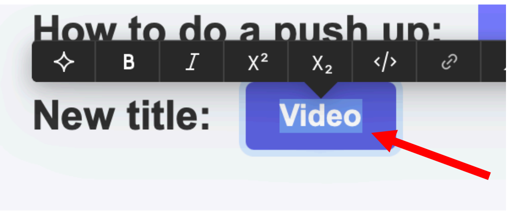

Figure A.9. Editing the name of the information document button

7. Now, let's change where the button links to.
- **Click the button**.
- Then, click the **' ⚙ ' icon** next to *Info button* (Figure A.10).

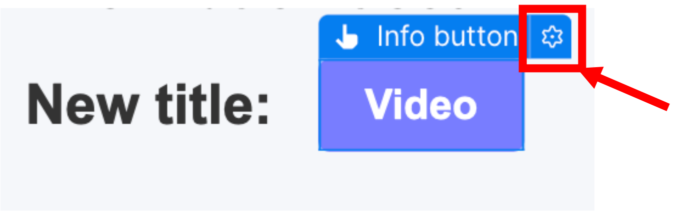

Figure A.10. Accessing the settings of a button

- Follow the steps within:
    - Step 7.1. – If you want to add a **document or image**
    - Step 7.2. – If you want to add a **link**
    - Step 7.3. – If you want to add a **video**

7.1. Adding a Document or Image

- Click on the **'File' icon** (Step 1 – Figure A.11)

- Click on **'Choose Attachment'** (Step 2 – Figure A.11)**, or 'Replace Attachment'** (Figure A.12) if a document is already linked, and the 'Assets' panel will open.

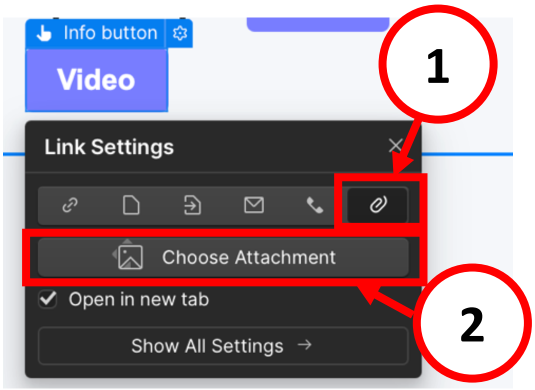

Figure A.11. Adding a file attachment to a button

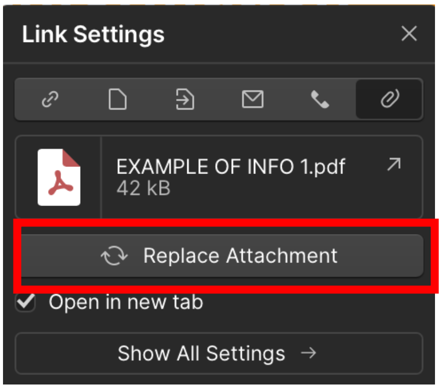

Figure A.12. Replacing attachment in a button

- Click the **'Upload' icon** (Figure A.13) and **upload the document or image** that you want to attach.
    - If you already have the file in the 'Assets' panel, just select it.

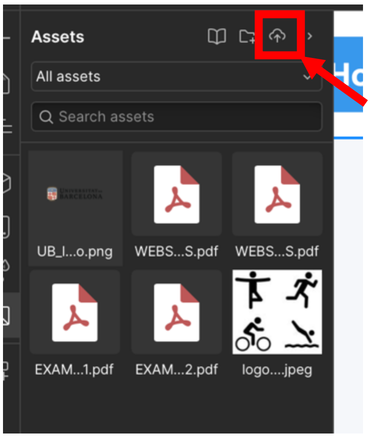

Figure A.13. Uploading a file in the assets panel

- **Publish the website** to apply and save your changes (For detailed instructions, refer to section *4. Publishing the Website*).

- Done! The document or image is now linked.

Note:
- *If you arrived here while following Example 1, click here to return to Example 1.*
- *If you arrived here while following Example 2, click here to return to Example 2.*

7.2. Adding a Link
- Click on the 'Link' icon (Step 1 – Figure A.14)

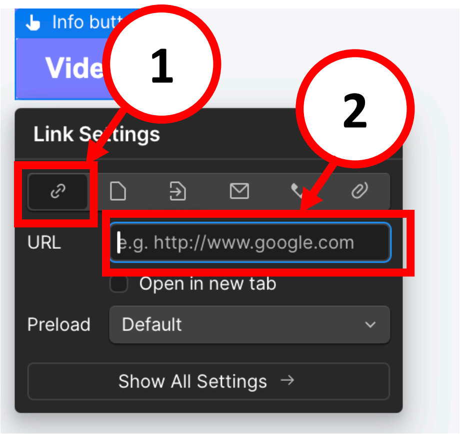

Figure A.14. Adding a link to a button

- Enter the desired link in the URL field (Step 2 - Figure A.14).
  - If you want the link to open in a new tab, check the option ***Open in a new tab***.

- **Publish the website** to apply and save your changes (For detailed instructions, refer to section *4. Publishing the Website*).

- Done! The link has been successfully added.

Note:
- *If you arrived here while following Example 1, click here to return to Example 1.*
- *If you arrived here while following Example 2, click here to return to Example 2.*

7.3. Adding a Video
There are two ways to add a video:

- **YouTube**
  - Upload the video on YouTube.
  - Set its visibility to **'Unlisted'**. This means only people with the link can view it.
  - Add video link as shown in **Step 7.2**.

- **Google Drive**
    - Upload you video to Google Drive.
    - Click **Share**.
    - Under "General access", click the down arrow.
    - Select 'Anyone with the link'.
    - Choose the role **'Viewer'**.
    - Click **Copy link**.
    - Click **Done**.
    - Add the link as shown in **Step 7.2**.

Publish the website to apply and save your changes (For detailed instructions, refer to section *4. Publishing the Website*).

Note:

- *If you arrived here while following Example 1, click here to return to Example 1.*
- *If you arrived here while following Example 2, click here to return to Example 2.*

2.2. How to delete a document, image, link or video

1. Open your site in Webflow. Click on **'Pages'** in the left menu (Step 1 – Figure A.15)

2. If you: (Step 2 – Figure A.15)

- Want to **delete an information document from the patients' page**:
  → Click ***Patients – Information***

- Want to **delete an information document from the staff's page**:
  → Click ***Staff – Information***

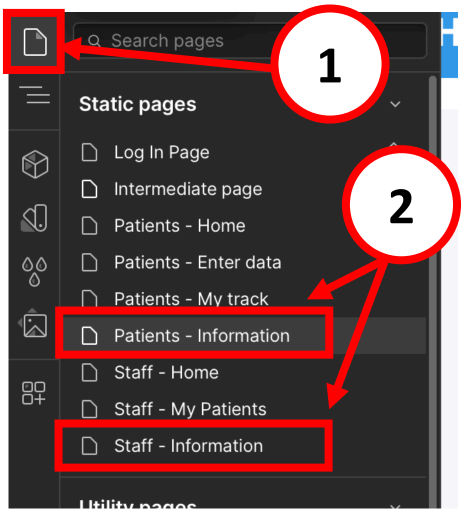

Figure A.15. Locating the Patients' and Staff's Information pages in the 'Pages' menu

3. Hover your mouse over the screen until you see **'Container Info' for the container you want to delete** (Step 1 – Figure A.16).

- **Click to select it.**

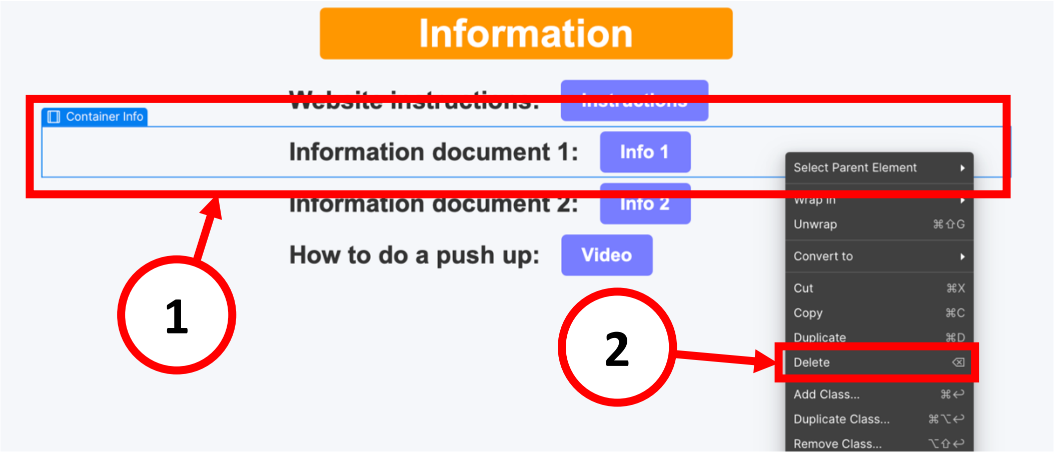

Figure A.16. Deleting a container containing a document, image, link or video

4. **Right-click** on the selected container.

5. Click ***Delete*** (Step 2 – Figure A.16).

6. **Publish the website** to apply and save your changes (For detailed instructions, refer to section *4. Publishing the Website*).

7. Done! The item has been removed.

Note:

- *If you arrived here while following Example 1, click here to return to Example 1.*
- *If you arrived here while following Example 2, click here to return to Example 2.*

3. Modifying the message system (level of difficulty: **2**/5)

Currently, the website allows:

- Patients to send messages to staff.
- Staff to read all messages sent by patients.
- Staff to send messages to individual patients.

In this section, we will go over how to:

- Restrict patients from sending messages (if you only want staff to communicate or send feedback to the patient)
- Remove the messaging system entirely (if you no longer need this feature)

3.1. Restrict patients from sending messages

1. In Webflow, click ***Open site*** to start editing your website. In the left menu, click on the **'Pages'** icon.

2. Click on ***Patients – Home*** page.

3. Click the **'Navigator' icon** (Step 1 – Figure A.17) in the left-menu.
This contains the structure of the different elements that appear on the page.

4. Click on the **'Send a message Heading'** (Step 2 – Figure A.17)

- Press the **Delete** key on your keyboard
- Or, right-click and select the option *Delete*.

Note: If you cannot find the 'Send a message Heading' in your screen's navigator, ***click on the arrow icons (>) to view the different elements inside each element***.

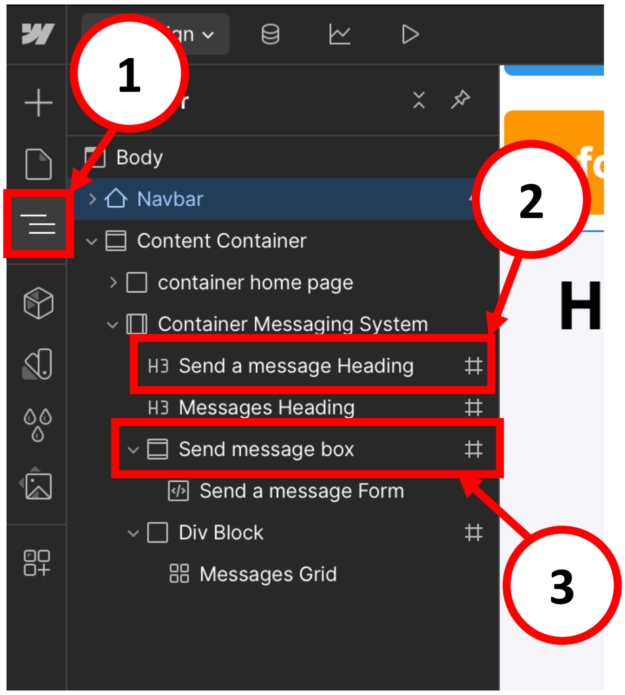

Figure A.17. Navigator menu on Webflow

5. **Repeat Step 4** for the **‘Send message box’** (Step 3 – Figure A.17). Figure A.18 shows how the page should look like now.

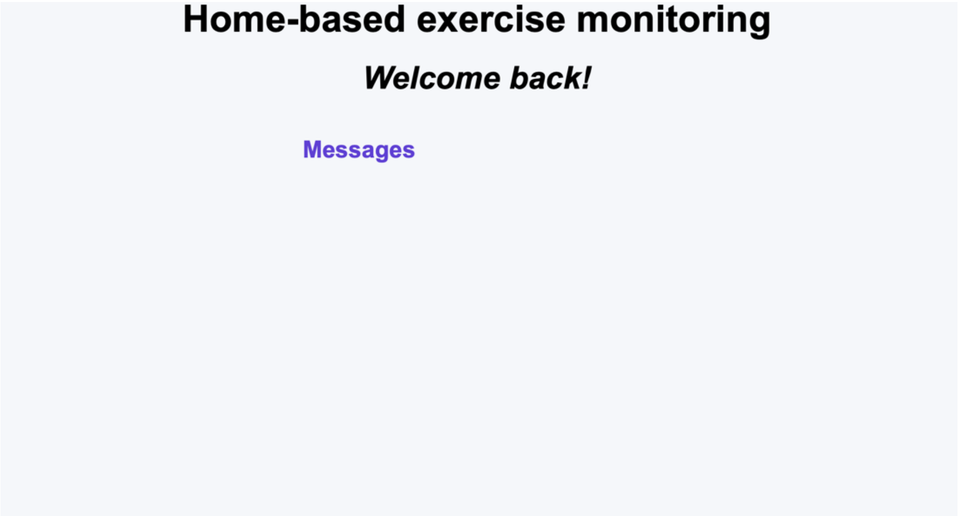

Figure A.18. ‘Patients – Home’ page without the ‘Send a message’ heading and box

6. Click the ‘Navigator’ icon again to close it.

7. Now, let’s modify the display of the remaining elements on the page: **Click on the ‘Container Messaging System’** (Figure A.19) to select it.

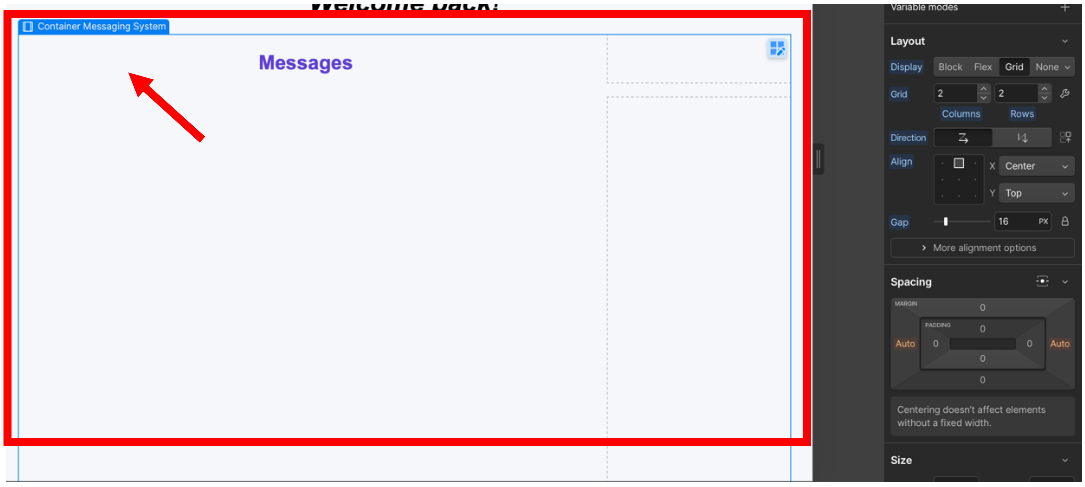

Figure A.19. Selecting the ‘Container Messaging System’

8. In the right panel, click ***Style*** to see the Style settings.

9. Then, change the **'Layout' settings**:
   - In 'Display', select **'Flex'**.
     (Step 1 – Figure A.20)
   - In 'Direction', choose **'↓'**.
     (Step 2 – Figure A.20)
   - In the **'x'** section of the 'Align' settings, choose **'Center'**.
     (Step 3 – Figure A.20)

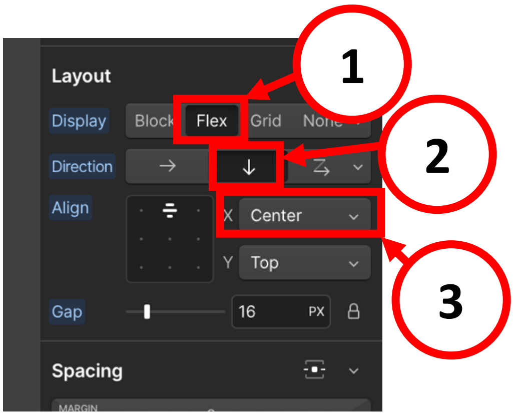

Figure A.20. Modifying the layout of the 'Container Messaging System'

10. **Publish the website** to apply and save your changes (For detailed instructions, refer to section *4. Publishing the Website*).

11. Perfect! You have successfully removed the patient's ability to send messages. Now, if you log into the website as a patient, you won't be able to send messages.

Note: If you arrived here while following Example 1, click here to return to Example 1.

3.2. Remove the messaging system

To remove entirely the messaging system, we will need to:
- Remove the patient's messaging system (in the 'Patients – Home' page)
- Remove the staff's messaging system (in the 'Staff – Home' page)

1. Open your site in Webflow. In the left menu, click on the **'Pages' icon**.

2. Click on the ***Patients – Home*** page.

3. Click on the 'Container Messaging System' to select it (Figure A.21).

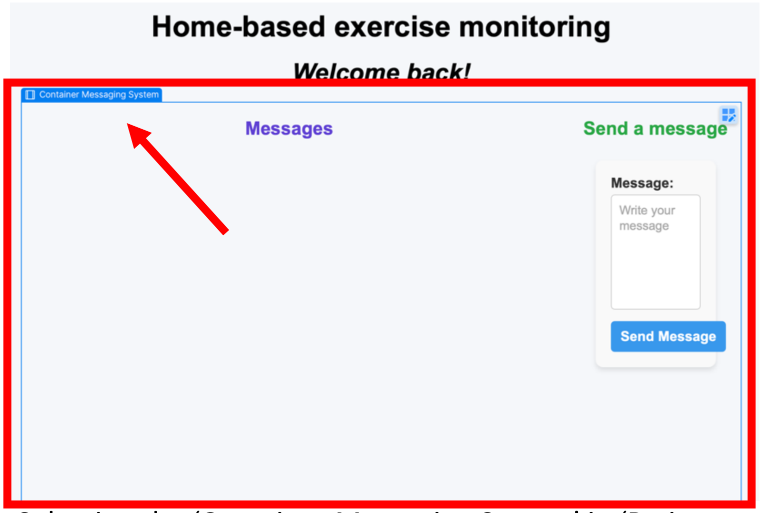

Figure A.21. Selecting the 'Container Messaging System' in 'Patients – Home' page

4. Once the container is selected, **right-click and** select the option ***Delete***.

5. In the left menu, click on the **'Pages' icon**.

6. Now, click on the ***Staff – Home*** page.

7. Click on the **'Container Messaging System Staff'** to select it (Figure A.22).

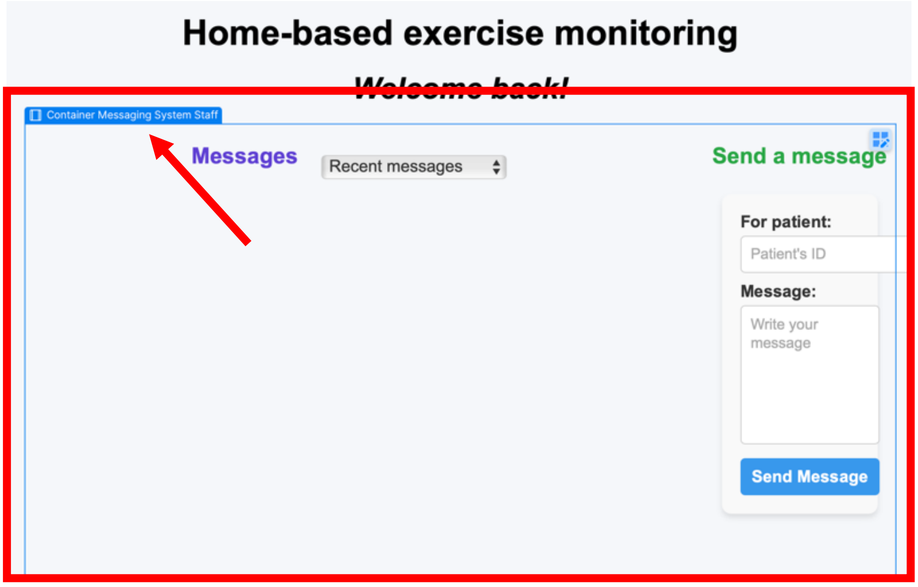

Figure A.22. Selecting the 'Container Messaging System Staff' in 'Staff – Home' page

8. Then, **right-click and** select the option ***Delete*.**

9. **Publish the website** to apply and save your changes (For detailed instructions, refer to section *4. Publishing the Website*).

10. Perfect, now both messaging systems have been deleted!

4. Modifying the form (level of difficulty: **4**/5)

Before making any kind of changes to the form, read section 4.1. Explaining the form's behaviour.

4.1. Explaining the form's behaviour

The form on the ***Patients – Enter data*** page (Figure A.23) is what allows patients to submit their daily information.

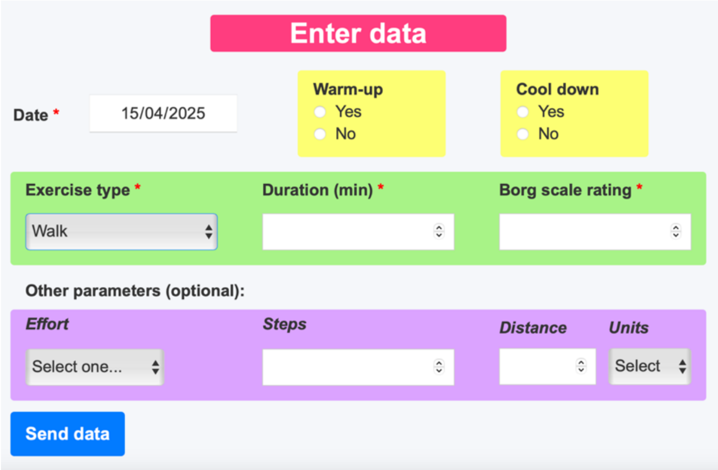

Figure A.23. Form in 'Patients – Enter data' page

The form can be modified, but here are some **key things to know** first:

1) **Required fields**
   The following fields must be filled to be able to submit the form:
   - Date
   - Exercise type
   - Duration (min)
   - Borg scale rating

   A required field can be changed to optional, or other fields can be made required. See section ***4.2. Managing Required Fields*** for more details.

2) **Graph connected fields**
   The values entered in these fields are used to generate graphs on the ***Patients – My track*** and ***Staff – My patients*** pages:
   - Duration (min)
   - Borg scale rating
   - Distance

   If not interested in tracking one of these values, you can:
   - Delete the field from the form (see section ***4.5. Deleting a field***)
   - And delete the matching graph in the patients and staff interface (see section ***5.2. Deleting a graph***)

Or, if interested in tracking a different numerical value, you can modify the 'Duration (min)' or 'Borg scale rating' fields (see section *Modifying a field*).

Lastly, if interested in tracking the data but not displaying it in a graph, you can delete the graph and leave the field (see section *5.2. Deleting a graph*).

Important: The **'Distance' field is different**. It is the only field that cannot be modified, it **must track distance**. So, if you do not want to track distance, you should:

- Delete the **Distance field** from the form (see section *4.5. Deleting a field*)
- Delete the **Units field** from the form (see section *4.5. Deleting a field*)
- Delete the **Distance graph** in ***Patients – My track*** and ***Staff – My patients*** (see section *5.2. Deleting a graph*)
- Delete the **Units filter** from ***Patients – My track*** and ***Staff – My patients*** pages (see section *5.3. Deleting a filter*)

3) **Exercise Type Field**
The options inside Exercise type dropdown **generate the activity buttons** in ***Patients – My track*** and ***Staff – My patients*** pages.

- The options in the Exercise type dropdown can be modified (See section *Editing Exercise Type Options*)
- When the 'Other' option from the dropdown is selected, the 'Other activity name' field appears for the user to fill in (Figure A.24).

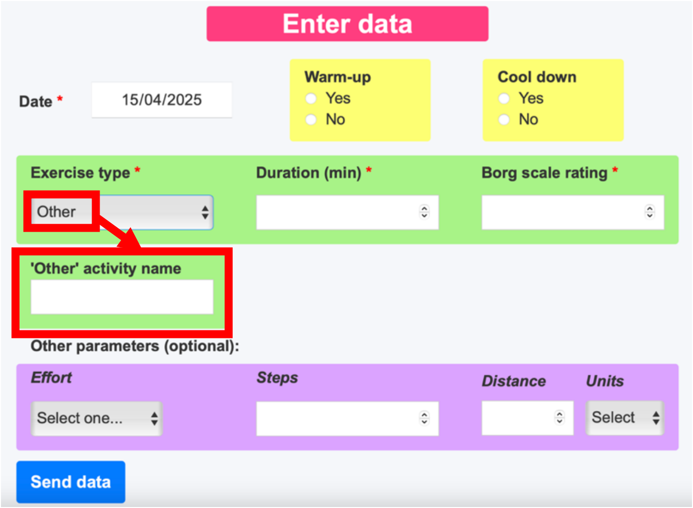

Figure A.24. Form in 'Patients – Enter data' page when 'Other' in 'Exercise type' is selected

- If needed, the Exercise Type field can be deleted (see section *Deleting Exercise Type Field*).

4) **Do not add new fields in the form.** Instead, **modify or delete the existing fields** as necessary.

4.2. Managing Required fields

The required fields in the form are (Figure A.25):

- *'Date'*
- *'Exercise type'*
- *'Duration (min)'*
- *'Borg Scale Rating'*

These are the fields that the user must fill out to submit the form. They are also marked with a red asterisk (*) to show that they are mandatory.

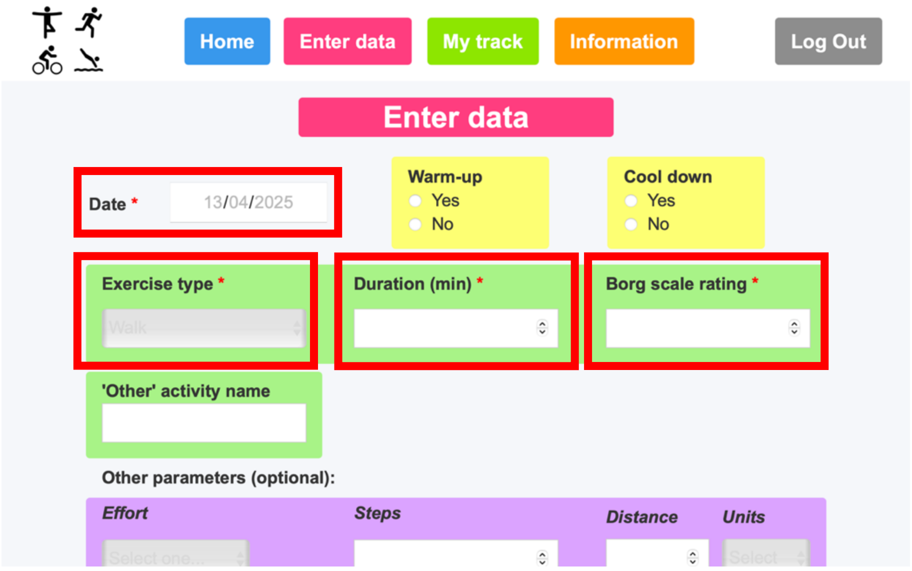

Figure A.25. 'Enter data' page with required fields marked

How to make a field required

1. Open your site in Webflow. In the left menu, click on the **'Pages'** icon.

2. Go to the ***Patients – Enter data*** page.

3. **Click on the 'box' of the field** you would like to make required (Step 1 – Figure A.26).

4. In the right menu, go to ***Settings*** (Step 2 – Figure A.26).

5. Then, check the option ***Required*** (Step 3 – Figure A.26).

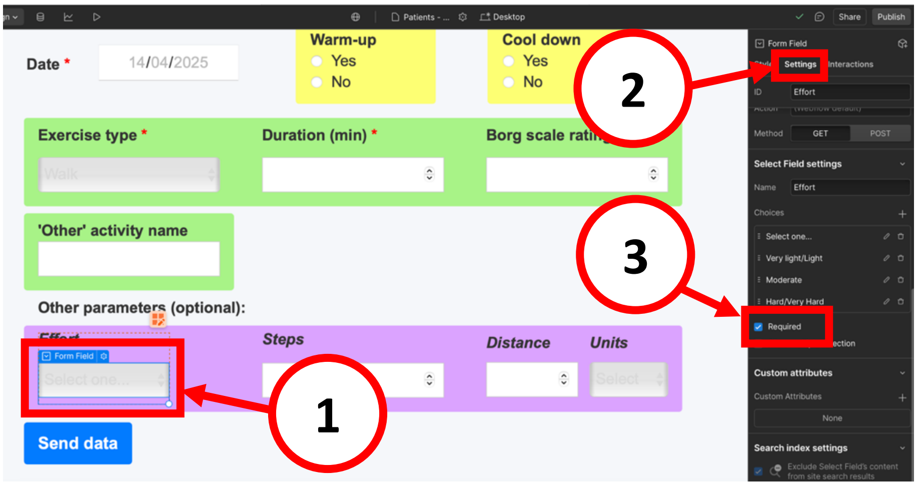

Figure A.26. Making a field in the form required

6. Perfect! Now, **double-click** on the **label** on top of the field:
- And **write an asterisk (*)**, to let know the users that the field is required.
- Then, **select the asterisk** and click in the **brush icon** (Figure A.27).

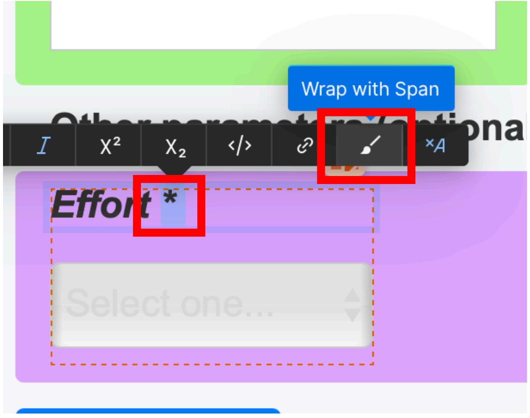

Figure A.27. Selecting the asterisk in the label

7. A 'Text Span' will be created to customize the asterisk:
- In the **Style** section of the right menu write **'Text Span' (Figure A.28)**
- And **the asterisk will turn red** like the other asterisks in the form.

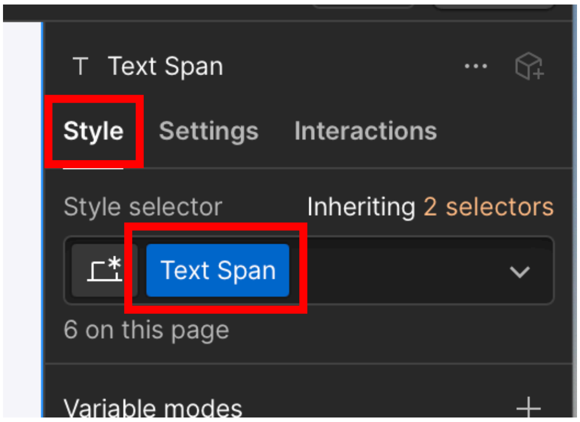

Figure A.28. Styling the asterisk in red

8. **Publish the website** to apply and save your changes (For detailed instructions, refer to section *4. Publishing the Website*).

9. Go to Xano and login.

10. Click on ***Database*** in the left menu.
    Note: If you do not find 'Database' because you have just logged into Xano, click on 'Free Instance' to get to where we are.

11. Then, click on the **'data_entries'** table.

12. Find the **column that matches the field** you made **required**.

13. **Right-click** on the name of the column and click on ***Settings***.

14. Scroll down the settings until you see the 'API configuration' section and enable **'Required'**. Then click on **'Update Column' (Figure A.29)**.

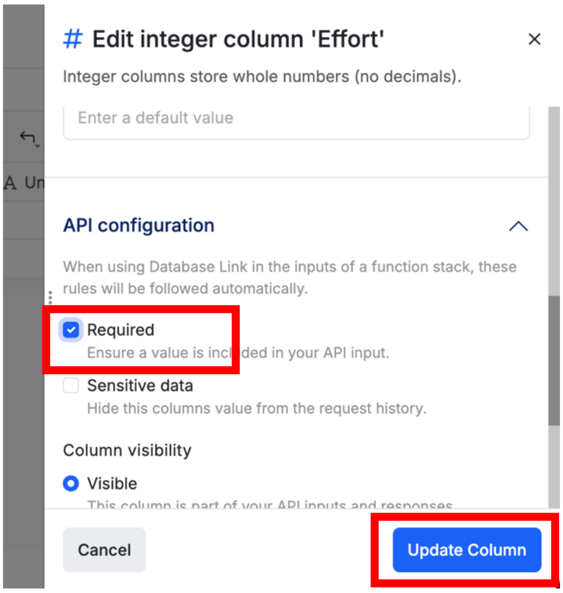

Figure A.29. Making required the corresponding column of the field in Xano.

15. Done! The field is now required.
    Note: If there are entries that do not have values in the field you made required, you will get a warning. To fix it, you can either add a value to those entries from Xano or delete them.

How to turn a required field into an optional one

1. Open your site in Webflow.

2. In the left menu, click on the **'Pages' icon**.

3. Click on the ***Patients – Enter data*** page.

4. **Click on the 'box' of the field** you would like to make optional (Step 1 – Figure A.30).

5. In the right menu, select ***Settings*** (Step 2 – Figure A.30).

6. Then, scroll down until you find **'*Required*'** and uncheck it (Step 3 – Figure A.30).

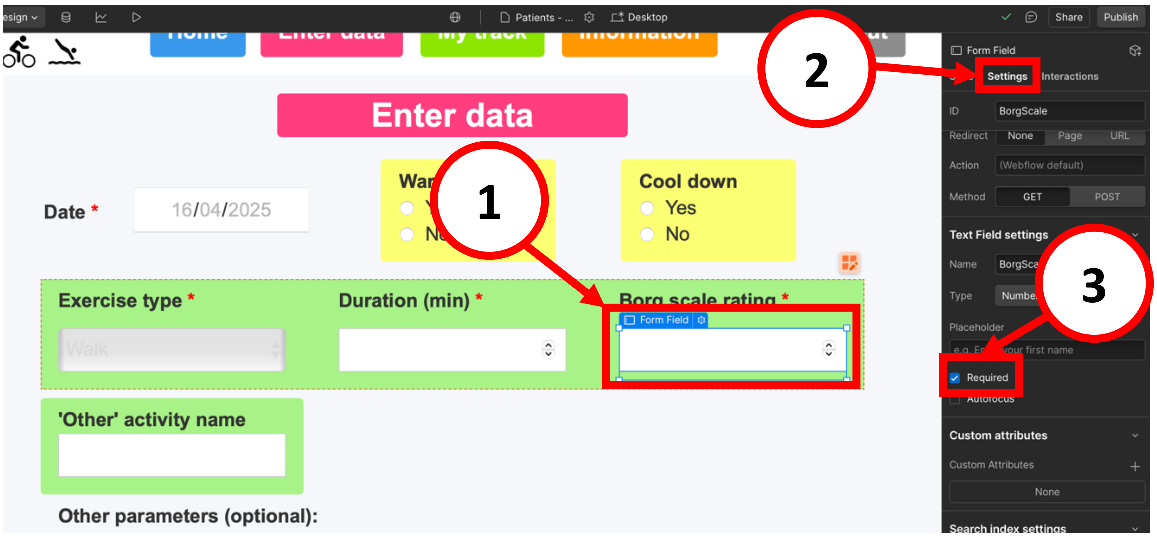

Figure A.30. Turning a required field in the form into an optional one

7. **Double click** on the **label** of the field to edit it, and **delete the red asterisk (*)**

8. **Publish the website** to apply and save your changes (For detailed instructions, refer to section *4. Publishing the Website*).

9. Go to **Xano**. Click on ***Database*** in the left menu.
Note: If you do not find 'Database' because you have just logged into Xano, click on 'Free Instance' to get to where we are.

10. Then, click on the **'data_entries'** table.

11. Find the **column that matches the field you made optional**.

12. **Right click** on the **name of the column** and click on ***Settings***.

13. Scroll down the settings until you see the 'API configuration' section and **uncheck *Required***. Then, click on ***Update Column*** (Figure A.31).

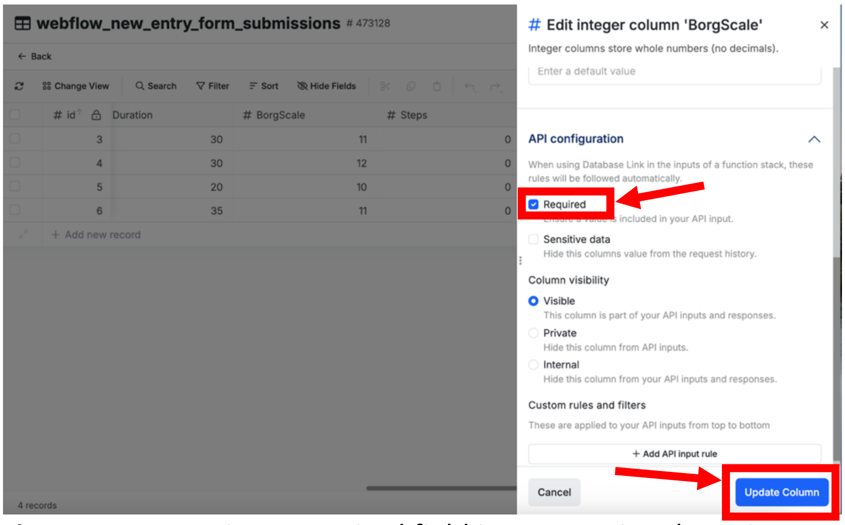

Figure A.31. Turning a required field into an optional one in Xano

14. Done! The field is now optional.

4.3. Managing the Exercise Type Field

Editing Exercise Type Options

The Exercise Type dropdown contains different options, which can be edited as follows:

1. In Webflow, click ***Open site*** to start editing your website (if you are not already doing so).

2. In the left menu, click on the **'Pages'** icon.

3. Click on ***Patients – Enter data***, to go to the 'Enter data' page.

4. **Double-click** the **'Exercise type' dropdown** to view its options (Figure A.32).

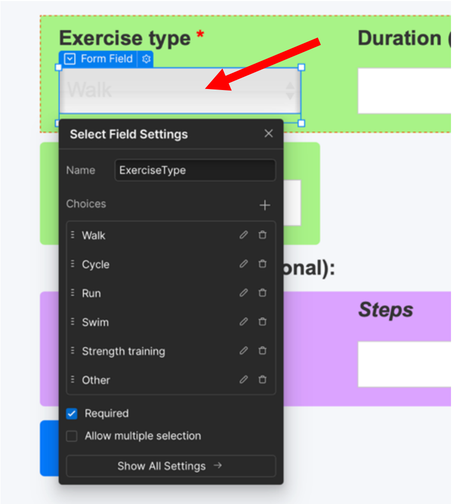

Figure A.32. Double-clicking on the 'Exercise type' dropdown

5. Now, follow the steps within:
- Step 5.1. – If you want to **add a new option**
- Step 5.2. – If you want to **delete an option**
- Step 5.3. – If you want to **manage the option named 'Other'** in the dropdown

5.1. Add a new option:

- Click the **'+' icon** next to Choices (Step 1 – Figure A.33).

- A new box will appear with empty **Text** and **Value** fields (Step 2 – Figure A.33).

- **Fill them in** with the new option, for example: *Text: Dance, Value: Dance*.
    - To save the option, click on any value in the list of Choices.
    - To reorder the options on the dropdown, drag the three-line icon next to the option (Figure A.34) to move it to another position (Figure A.35).

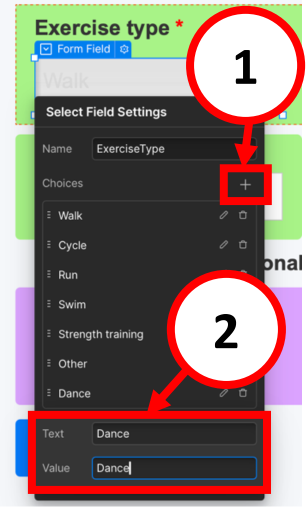

Figure A.33. Adding a new option in the Exercise type option

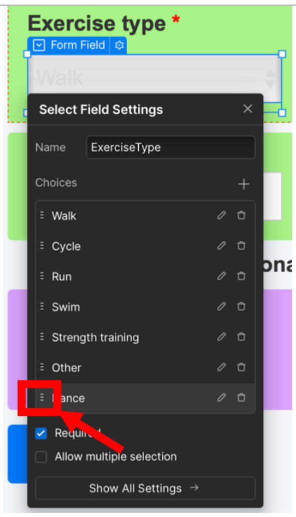

Figure A.34. Clicking on the three-line icon to move the option

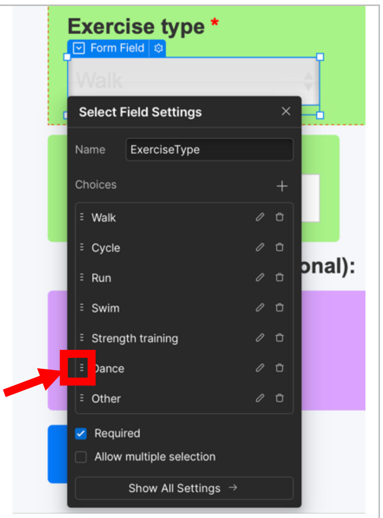

Figure A.35. Added option in a new position

- **Publish the website** to apply and save your changes (For detailed instructions, refer to section *4. Publishing the Website*).

- Great! You have added a new option in the Exercise Type drowdown.

Note: If you arrived here while following Example 1, click here to return to Example 1.

5.2. Delete an option:

- Click on the **trash can icon next to the option** you want to delete (Figure A.36)

- **Publish the website** to apply and save your changes (For detailed instructions, refer to section *4. Publishing the Website*).

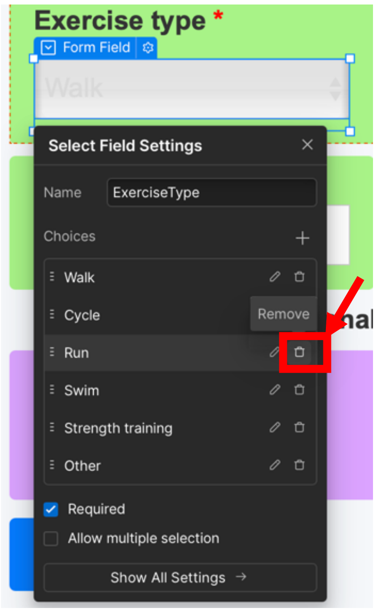

Figure A.36. Removing an option in the dropdown

- Now, go to Xano.

- In the left menu, click on **Database**.
Note: If you do not find 'Database' because you have just logged into Xano, click on 'Free Instance' to get to where we are.

- Click on the **'data_entries'** table.

- Delete all the existing entries:
  - **Check the box next to 'id'** (Step 1 – Figure A.37)
  - Click on ***Delete*** (Step 2 – Figure A.37)

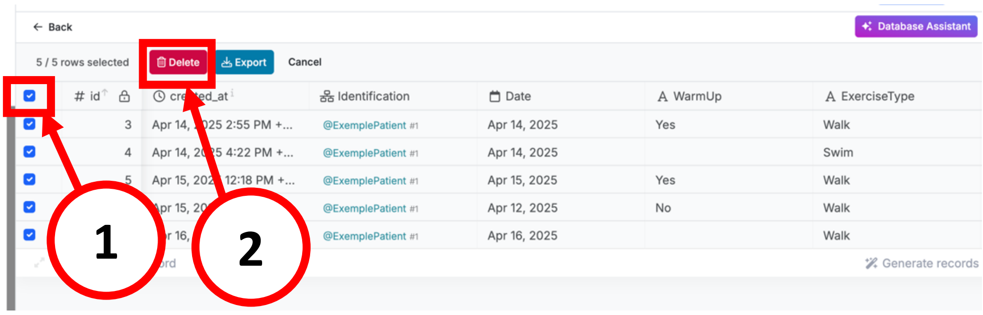

Figure A.37. Removing an option in the dropdown

Now, once new entries are submitted, the activity buttons on ***Patients – My track*** and ***Staff – My patients*** pages will be created with the updated dropdown options.

- Done! The option has been properly deleted from the dropdown.

5.3. **Manage the 'Other' option:**
When the **'Other' option is selected** in the dropdown of the website, **the field *'Other' activity name* appears** for the user to fill in.
If this extra field is not needed, you can:
   a. Delete the 'Other' option from the dropdown
   b. Delete the *'Other' activity name* from the form.
      Or, if you need a separate text field, delete the 'Other' option from the dropdown and modify the field (see section *Modifying a field*).

Deleting Exercise Type Field

If you are not interested in having the Exercise type field in your website, because the information you want to track with *'Duration (min)'* and *'Borg scale rating'* fields does not need to be filtered with buttons, you can delete it by following the next steps:

1. Open your site in Webflow. In the left menu, click on the **'Pages'** icon.

2. Click on the ***Patients – Enter data*** page.

3. **Right-click** on the **'Exercise type' select field** in the form (**Figure A.38**).
- Then, select ***Delete***.

4. **Right-click** on the **Exercise Type field label** (Figure A.39)
- Then, select ***Delete***.

Note: If you have trouble selecting the Exercise Type label, click the green container and temporarily change its display to 'Block' (right panel). After deleting it, switch the display setting back to its original layout ('Grid').

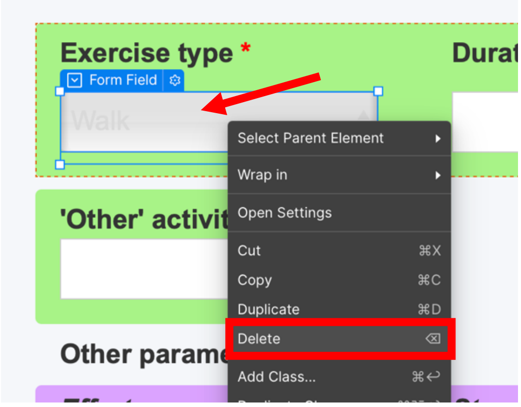

Figure A.38. Deleting the Exercise type select field on 'Patients – Enter data'

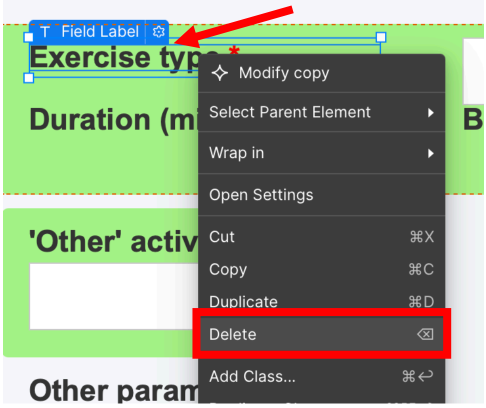

Figure A.39. Deleting the Exercise type label on 'Patients – Enter data'

5. Your form should now look like the one in **Figure A.40**.

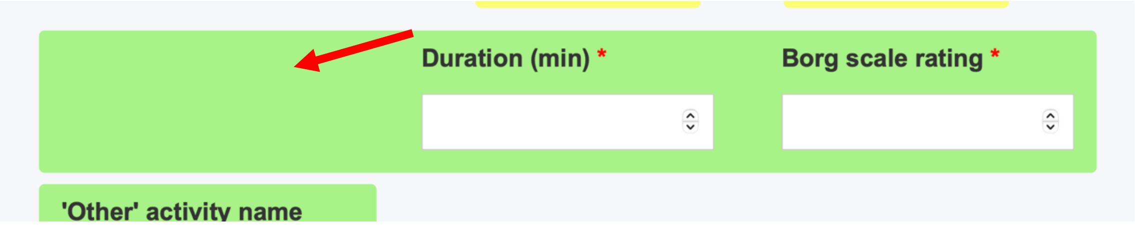

Figure A.40. Form without the *Exercise Type* field and label

6. Now, let's reorder the form so it looks better visually:
- Click on the **label *Duration (min)* (Step 1 – Figure A.41)**

- On the right panel, click the **'Style' tab (Step 2 – Figure A.41)**

- Under *Position*, set ***Column start* and *end* to 1 and 1 (Step 3 – Figure A.41)**

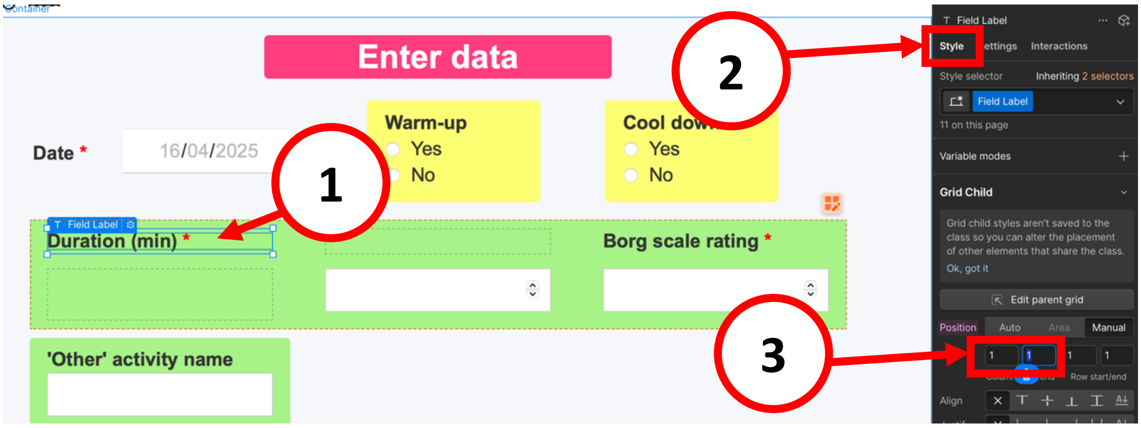

Figure A.41. Moving the *Duration (min)* field label

- Click on the **field that was below *Duration (min)*** and do the same:
  - In the 'Style' tab, change its ***Column start/end*** to **1** and **1** as well.

- Next, click on the ***Borg scale rating* label**:
  - Go to **'Style'** and set its ***Column start/end*** to **2** and **2**.

- Do the same for the ***Borg scale rating* field**:
  - Click on it, go to **'Style'**, and set its ***Column start/end*** to **2** and **2**.

- Your form should now look like the one in **Figure A.42**.

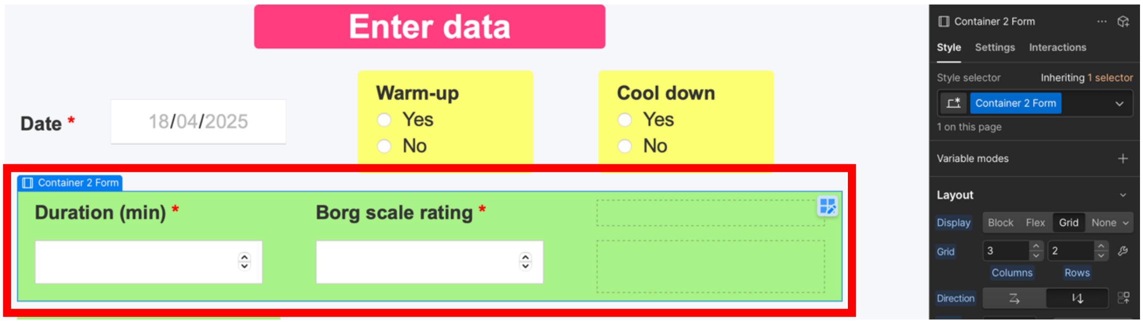

Figure A.42. Reordered form without the Exercise Type select field

7. Lastly, click on the green container named **‘Container 2 Form’**:
- And, on the **Style** settings of the right panel:
  - Change the Grid Layout to **2 columns**, as shown in **Figure A.43**.

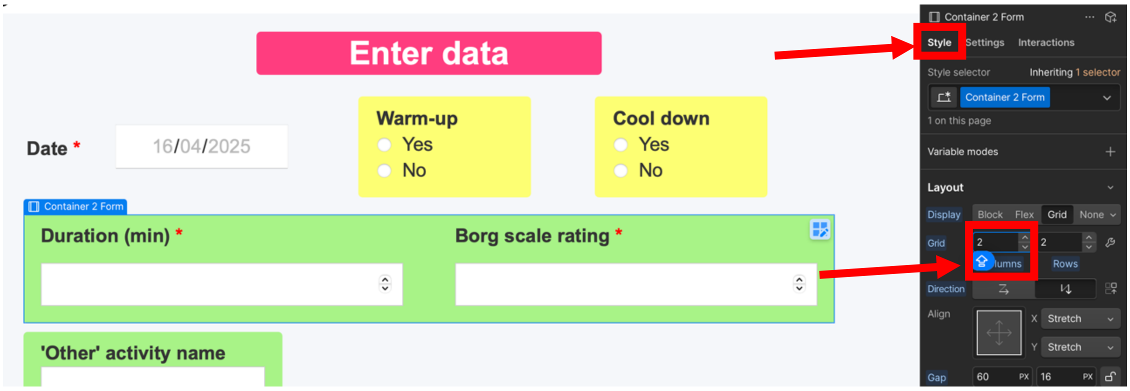

Figure A.43. Changing the layout to two columns

8. Perfect! Now, in the left menu, click on the **‘Pages’** icon.

9. Click on the ***Patients – My track*** page.

10. Find the container named **‘Container Activity Buttons’** (Figure A.44).
- **Right click** on it.
- And select ***Delete***.

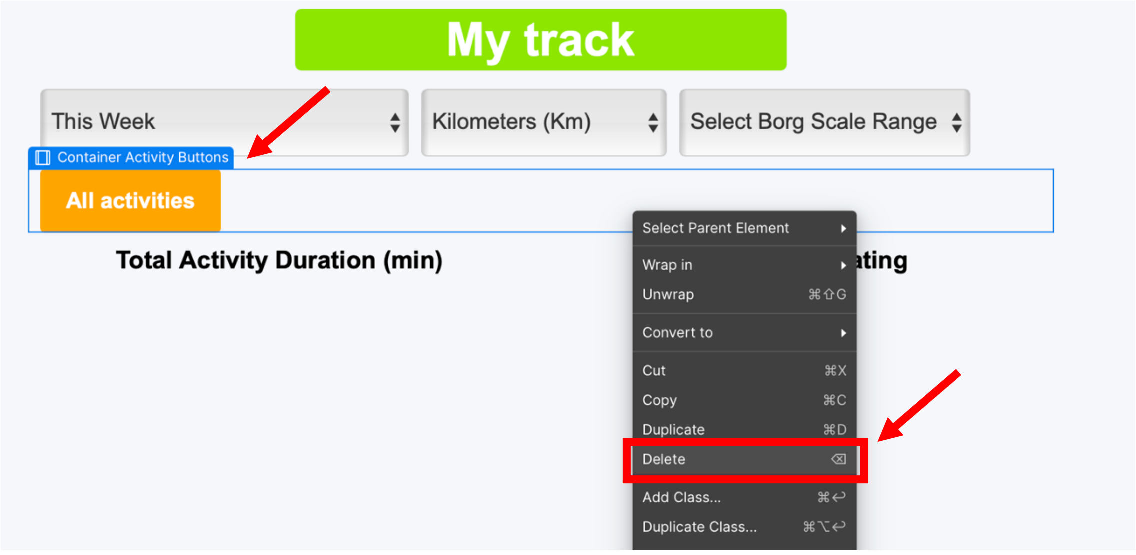

Figure A.44. Deleting the ‘Container Activity Buttons’ container

11. In the left menu, click on the **‘Pages’** icon.

12. Click on the ***Staff – My patients*** page.

13. Again, find the container named **‘Container Activity Buttons’**:
- **Right-click** it.
- And select ***Delete***.

14. **Publish the website** to apply and save your changes (For detailed instructions, refer to section *4. Publishing the Website*).

15. Perfect! Now, go to **Xano**. From the left menu, select ***Database***.
Note: If you do not find 'Database' because you have just logged into Xano, click on 'Free Instance' to get to where we are.

16. Click on the **'data_entries'** table.

17. Locate the **ExerciseType** column (**Figure A.45**).

18. **Right-click** on the name of the column (**Figure A.45**).
- Then, select ***Delete***.

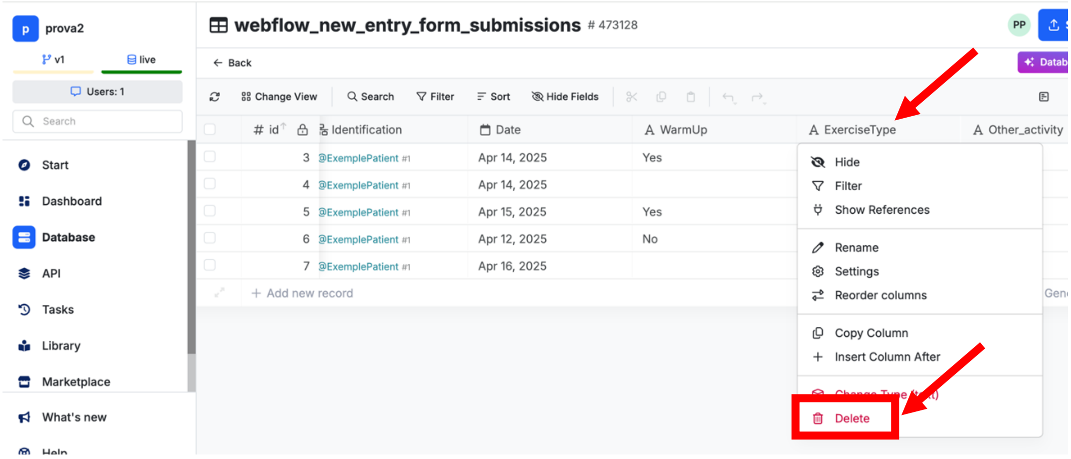

Figure A.45. Deleting the ExerciseType column in Xano

19. Done! You have successfully completely deleted the ExerciseType field.

4.4. Changing the remaining fields

Modifying a field

The fields of the form can be modified to track something else. To do so, follow the next steps:

1. Open your site in **Webflow**. In the left menu, click on the **'Pages'** icon.

2. Click on the ***Patients – Enter data*** page.

3. **Double-click on name of field** you would like to change (Figure A.46).
- Then, delete the text of existing label and **type in the new label** (Figure A.47).

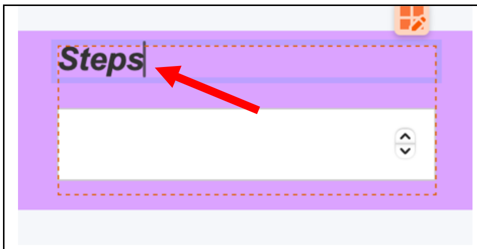	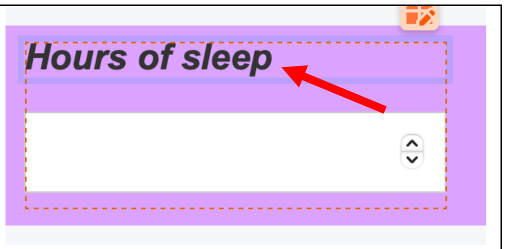
**Figure A.46.** Double-clicking on a field label to edit it	**Figure A.47.** Typing the new label of an existing field

4. Perfect! Now, **publish the website** to apply and save your changes (For detailed instructions, refer to section *4. Publishing the Website*).

5. Now, only follow the steps below if you are modifying a field other than 'Duration', 'Borg Scale', 'Distance', or 'Units'. If you modified one of those fields, you have already completed the necessary changes.

6. Let's change the name of that field in the database. To do so, go to **Xano**.

7. In the left menu, click on **Database**.
Note: If you do not find 'Database' because you have just logged into Xano, click on 'Free Instance' to get to where we are.

8. Then, click on the **'data_entries'** table.

9. Look for the **column** that corresponds to the field name you have just changed on the form.

10. **Right-click** on the **name of that column**.

11. Select ***Rename*** (Figure A.48).

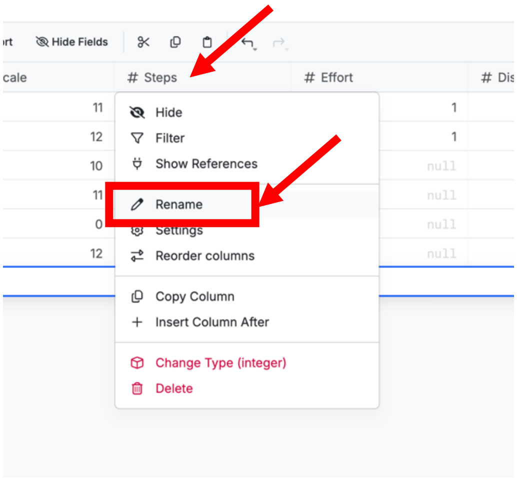

Figure A.48. Renaming the column matching the field changed

12. **Rename it** to something that reflects what the new field tracks.
*Note: **Do not use black spaces in the name**.*
E.g. Use 'HoursSleep' instead of 'Hours of sleep'.

- A confirmation banner will appear. Click **'Confirm'**.

- Then, a tab will open on the right. Click **'Update references'** (Figure A.49).

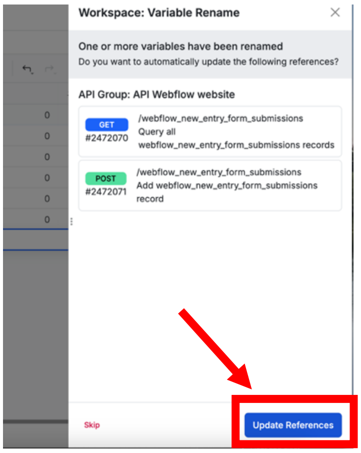

Figure A.49. New tab after confirmation banner

13. Great! Now, while still in Xano:
- Click on **API** in the left menu (Step 1 – Figure A.50).

14. Then, click on the folder named **'API Webflow website'** (Step 2 - Figure A.50).

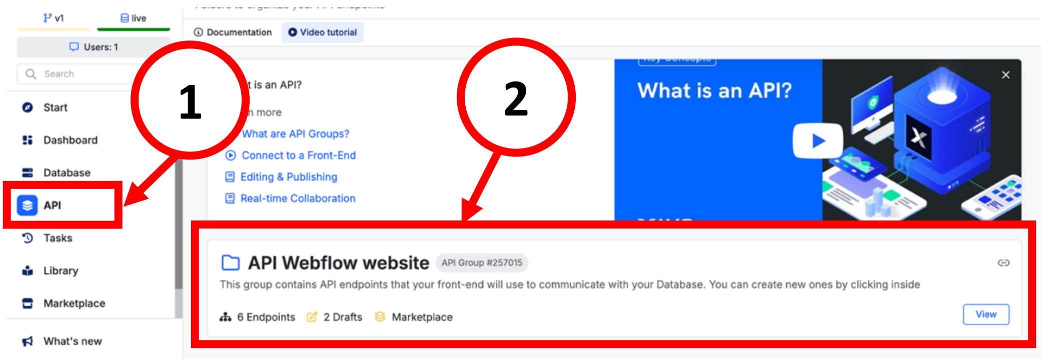

Figure A.50. Accessing the 'API Webflow website' folder in Xano

15. Inside the folder, there is a section named **'data_entries'**. In that section, you will see **POST data_entries** marked as ***'Draft'*** (Figure A.51).

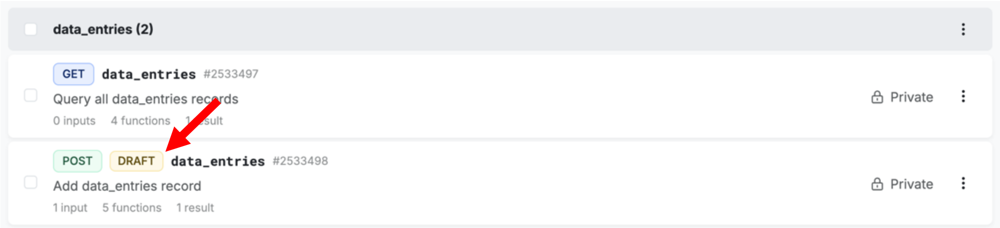

Figure A.51. Section 'data_entries' inside the 'API Webflow website' folder in Xano

16. Click on **POST data_entries**

17. Then, click on the **'Publish'** button **(Figure A.52).**

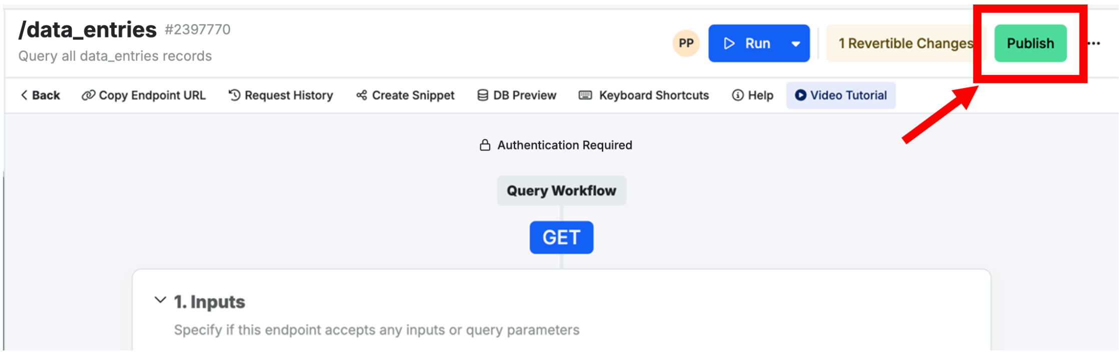

Figure A.52. 'Publish' button inside a subsection

18. A tab will appear in the right side of the screen.
- Click the **'Publish'** button again (See Figure A.53).

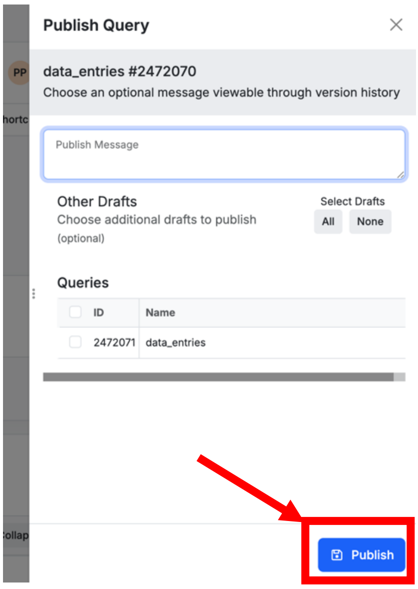

Figure A.53. 'Publish' button in the new tab

19. Return to the previous screen.

20. Congratulations! You have successfully modified a field in the form.

Changing a field to an open text field

1. Open your site in **Webflow**. In the left menu, click on the **'Pages'** icon.

2. Click on the ***Patients – Enter data*** page.

3. **Double-click on name of field** you would like to turn into an open text field.
- Then, delete the text of existing label and **type in the new label (Figure A.54)**.

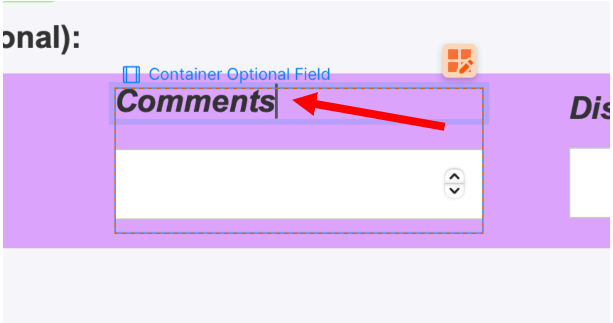

Figure A.54. Typing the new label of an existing field

4. Now, **click the field** you would like to turn into an open text field (Step 1 - Figure A.55).
   Note: You can only perform this action on fields that are currently set to a numerical type.

- On the ***Settings*** of the right panel (Step 2 - Figure A.55), scroll down to the '*Text Field settings'* section.
- Then, next to ***Type***, select ***Plain*** (Step 3 – Figure A.55).
  Note: It is very important that you only change the Type setting, do not modify anything else in Settings section.

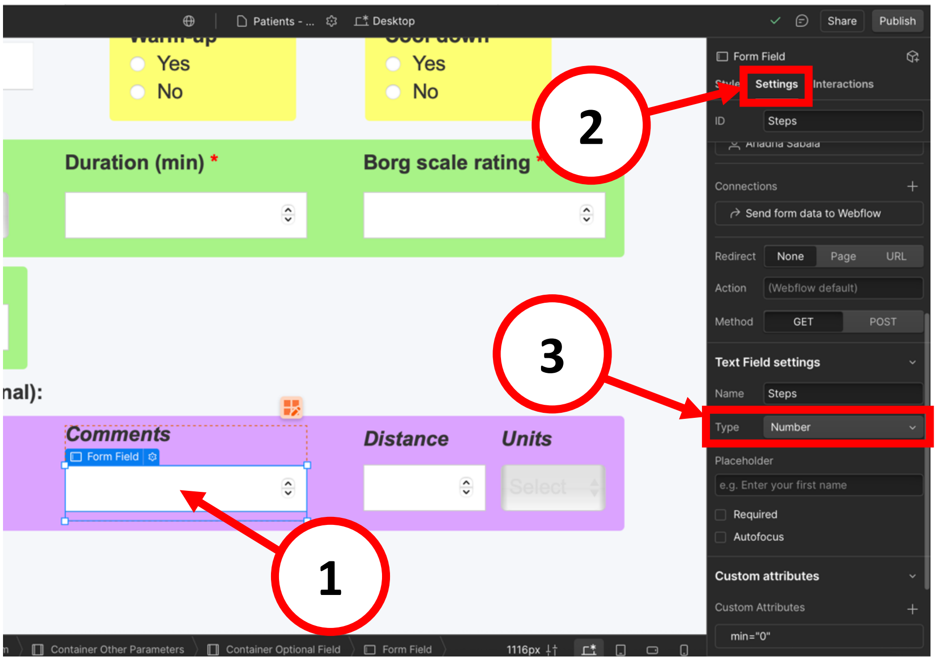

Figure A.55. Changing a field's Type

5. Perfect! Now, **publish the website** to apply and save your changes (For detailed instructions, refer to section [4. Publishing the Website](#)).

6. Now, go to **Xano**.

7. In the left menu, click on **Database**.

8. Then, click on the **'data_entries'** table.

9. Look for the **column** that corresponds to the field name you have just changed on the form.

10. **Right-click** on the **name of that column** and select ***Rename*** (Figure A.56).

- **Rename it** to something that reflects what the new field tracks.
  Note: ***Do not use black spaces in the name****.*
  E.g. Use 'HoursSleep' instead of 'Hours of sleep'.

- A confirmation banner will appear. Click **'Confirm'**.

- Then, a tab will open on the right. Click **'Update references'**

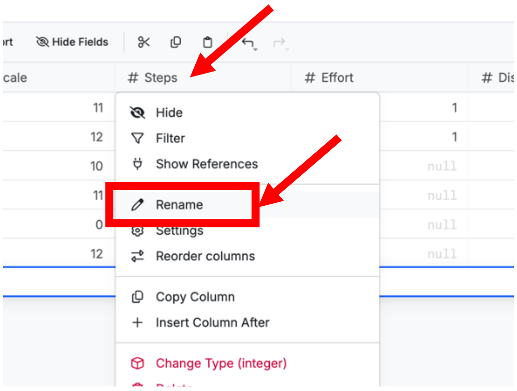

Figure A.56. Renaming the column matching the field changed

11. **Right-click again** the **name of the same column** (now renamed):
- And select ***Change Type (integer)*** (Figure A.57).
- Change it to ***text*** (Figure A.58).
- Click ***Save*** (Figure A.58).

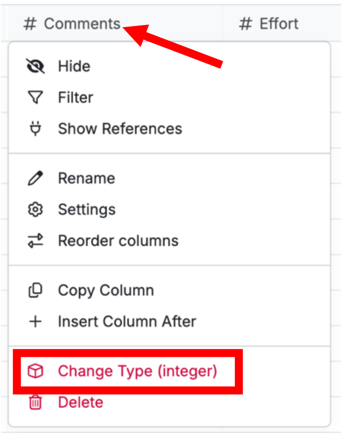

Figure A.57. Selecting 'Change Type (integer)

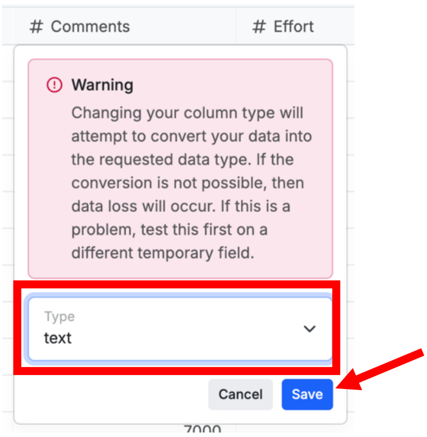

Figure A.58. Changing Type to 'text'

12. Great! Now, while still in Xano:
- Click on **API** in the left menu (Step 1 – Figure A.59).

13. Then, click on the folder named **'API Webflow website'** (Step 2 - Figure A.59).

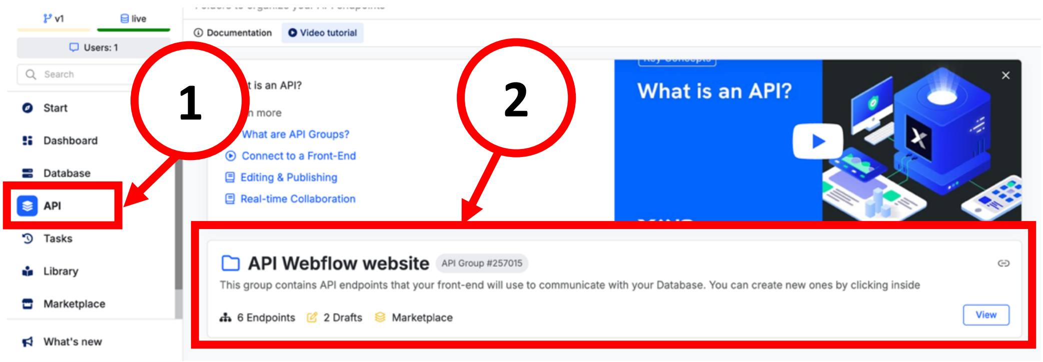

Figure A.59. Accessing the 'API Webflow website' folder in Xano

14. Inside the folder, there is a section named **'data_entries'**. In that section, you will see **POST data_entries** marked as ***'Draft'*** (Figure A.60).

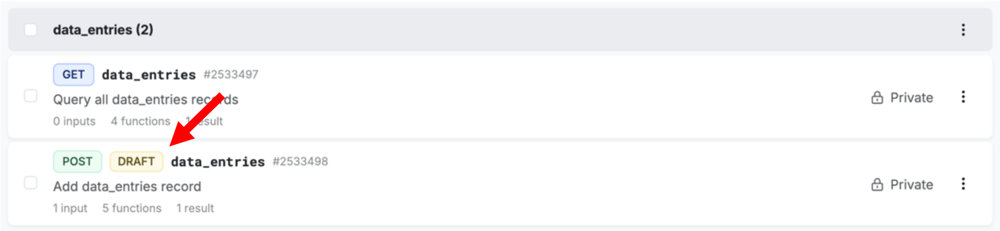

Figure A.60. Section 'data_entries' inside the 'API Webflow website' folder in Xano

15. Click on **POST data_entries**.

16. Then, click on the **'Publish'** button **(Figure A.61).**

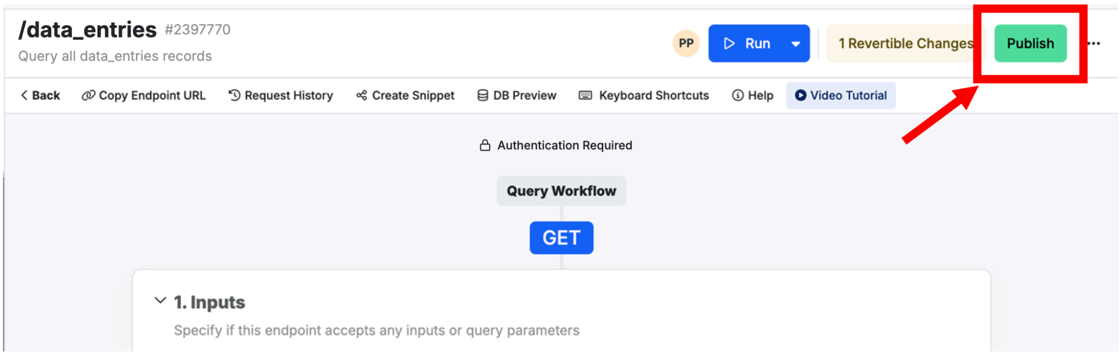

Figure A.61. 'Publish' button inside a subsection

17. A tab will appear in the right side of the screen.
- Click the **‘Publish’** button again. (See Figure A.62)

18. Return to the previous screen.

19. **Congratulations!** You have successfully changed a field to an open text field.

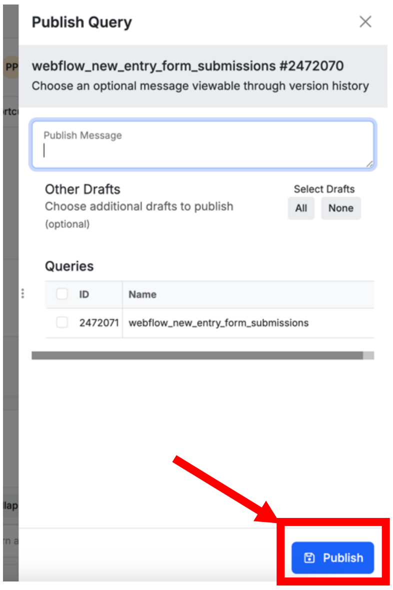

Figure A.62. ‘Publish’ button in the new tab

4.5. Deleting a field

1. Open your site in **Webflow**. In the left menu, click on the **‘Pages’** icon.

2. Click on the ***Patients – Enter data*** page.

3. Locate the question you want to delete:

- If its inside a container, **right-click the container** and select **‘Delete’** (Figure A.63 and Figure A.64).

- If it is not in a container, delete both the **‘Field Label’ and the ‘Form Field’** separately by **right clicking on each one** and selecting **‘Delete’** (Figure A.65).

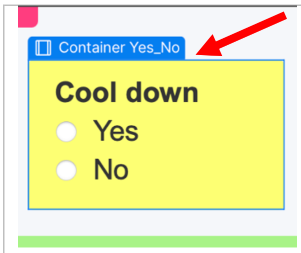

Figure A.63. ‘Cool down’ question inside a container

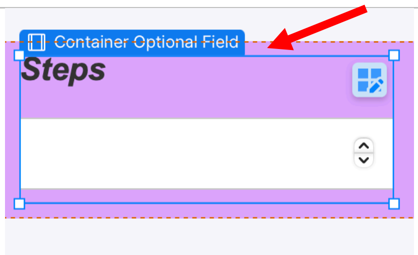

Figure A.64. ‘Steps’ question inside a container

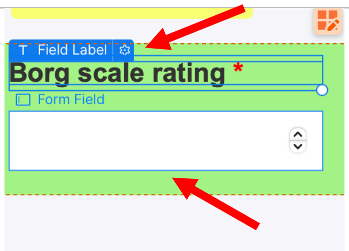

Figure A.65. ‘Borg scale rating’ question separately – Field Label and Form Field.

4. Go to **Xano**.

5. From the left menu, select **Database**.

6. Click on the **'data_entries'** table.

7. **Find the column that matches the question you have just deleted** from the form. E.g. If you deleted the question 'Cool down', look for the column named 'CoolDown'.

8. **Right-click** on the title of that column and select ***Delete*** **(Figure A.66)**.

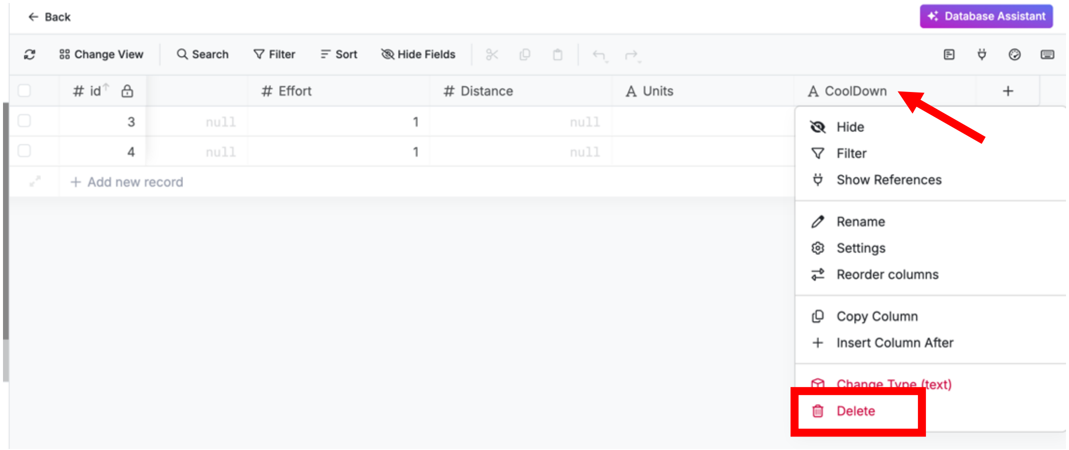

Figure A.66. Deleting the 'CoolDown' column in the 'data_entries' table

9. Select ***Delete*** again when the confirmation banner appears (Figure A.67)

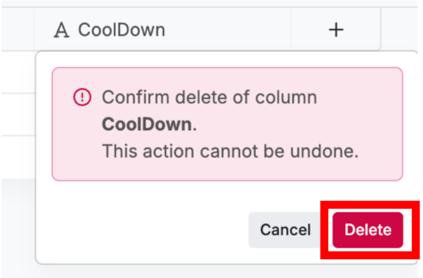

Figure A.67. Confirmation banner when deleting a column in the 'data_entries' table

10. And done! You have successfully removed a question from the form.

Important: If the field you delete is one that created a graph:
- Duration (min)
- Borg scale rating
- Distance
- Units

Then, you will also need to delete the corresponding graph from:
- ***Patients – My Track*** page
- ***Staff – My Patients*** page

Because they will no longer have data to display. To learn how to do it, go to section ***5.2. Deleting a graph.***

5. Modifying the graphs (level of difficulty: **2**/5)

There are three graphs in the 'My track' (patient's interface) and the 'My patients' page (staff's interface):

- 'Total Activity Duration (min)' graph
- 'Borg Scale Rating' graph
- 'Distance (*units*)' graph

5.1. Changing the title of a graph

Follow the next steps to change the title of a graph.

Note: It is not recommended to change the 'Distance (units)' graph title because this graph is specifically meant to track distances.

1. Go to Webflow. Make sure you are in 'Designer' mode.

2. In the left menu, click on the **'Pages' icon**.

3. Click on the ***Patients – My Track*** page.

4. **Double click on the title** you would like to modify (Figure A.68) and **change the title** as desired.

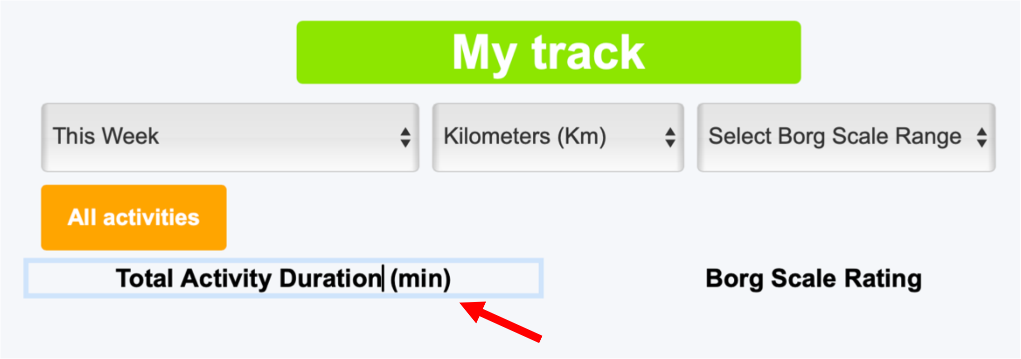

Figure A.68. Modifying the title of a graph

5. In the top-menu of the screen, change the display view to **'Mobile (L)'** (Figure A.69).
   This will allow us to update the mobile version of the title.

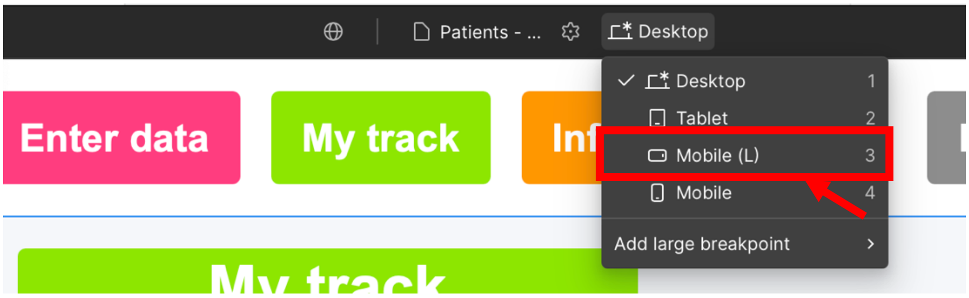

Figure A.69. Changing the display to 'Mobile (L)'

6. In the **Settings** of the right-menu:

- In the **Slider settings** section, use the **arrows (Step 1 – Figure A.70)** to change between slides **until you see the title you want to update**.

- Once you have found it, **double-click on the title (Step 2 – Figure A.70)** and change it to the same title you set earlier.

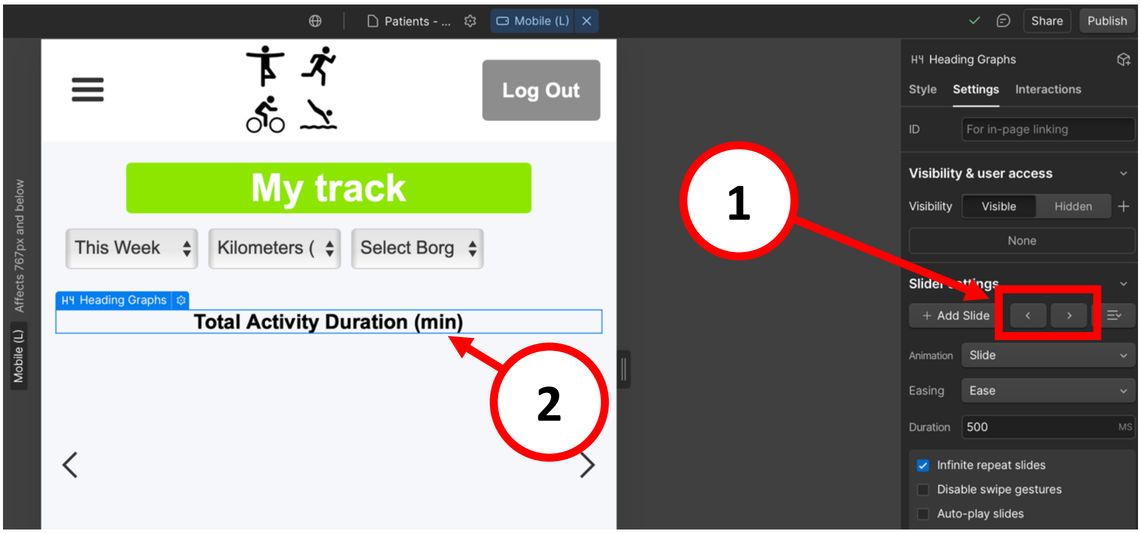

Figure A.70. Modifying the title of a graph in the mobile display view

7. **Click the 'X' next to 'Mobile (L)'** at the top of the screen to exit the mobile view.

8. Done! You have successfully changed the title of both the desktop and mobile views of the graph.

9. In the left menu, click on the **'Pages' icon**.

10. Click on the ***Staff – My Patients*** page and **repeat Steps 4 to 7 for this page**.

11. **Publish the website** to apply and save your changes (For detailed instructions, refer to section *4. Publishing the Website*).

5.2. Deleting a graph

If there is a graph you are not interested in, it can be deleted by following these steps:

1. Go to Webflow. Make sure you are in 'Designer' mode.

2. In the left menu, click on the **'Pages' icon**.

3. Click on:

- ***Patients – My Track*** page: if you want to delete the graph from the **patient interface.**

- ***Staff – My Patients*** page: if you want to delete the graph from the **staff interface.**

- If you want to delete the graph from **both interfaces**: follow the next steps twice (first for the ***Patients – My Track*** page, and then for the ***Staff – My Patients*** page).

4. Once you are on the desired page:
   - Click on the **Navigator icon** in the left-menu **(Step 1 – Figure A.71)**.

   *Note: The Navigator contains the structure of the different elements that appear on the page. The **arrow icons (>)** are used to view the different components inside each element.*

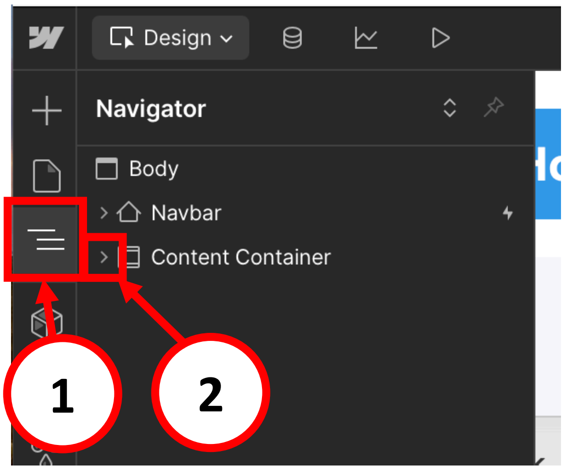

Figure A.71. Opening the Navigator menu

5. Click the **arrow icon (>)** next to **'Content Container'** (Step 2 - Figure A.71).

6. Click the **arrow icon (>)** next to **'Container Grid'** (Step 1 - Figure A.72).

   Inside, you will see **three 'Container Graphs Grid' elements** (in blue - Figure A.72), each of them represents a graph in the desktop view:
   - 1st 'Container Graphs Grid' → 'Total Activity Duration (min)' graph
   - 2nd 'Container Graphs Grid' → 'Borg Scale Rating' graph
   - 3rd 'Container Graphs Grid' → 'Distance (*units*)' graph

7. **Click on the 'Container Graphs Grid' that corresponds to the graph you want to delete** (Step 2 – Figure A.72).

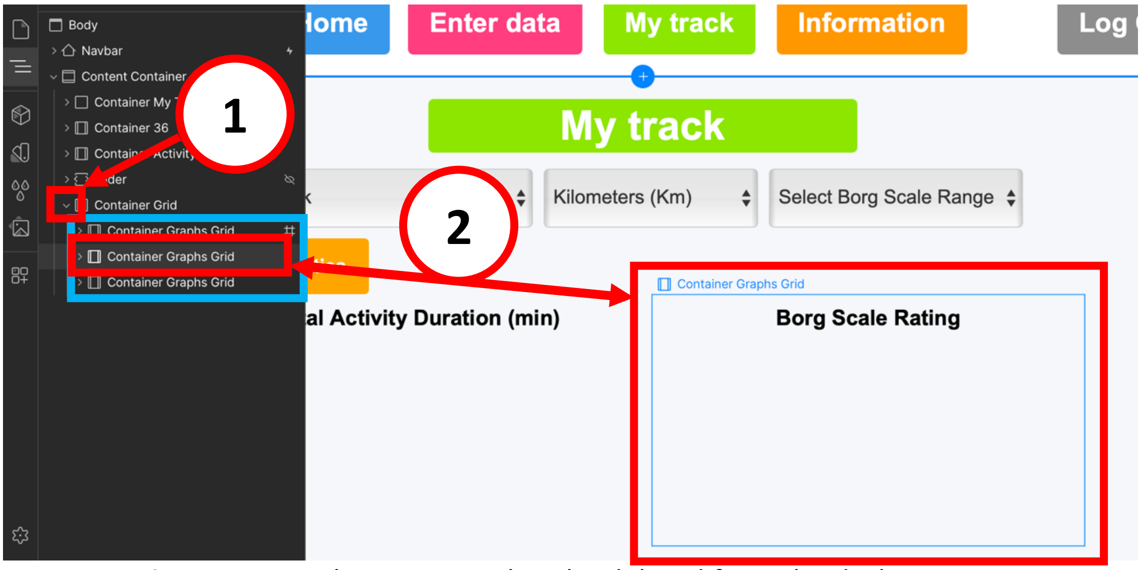

Figure A.72. Selecting a graph to be deleted from the desktop view

8. **Right-click** on it and select ***Delete*** (Figure A.73).

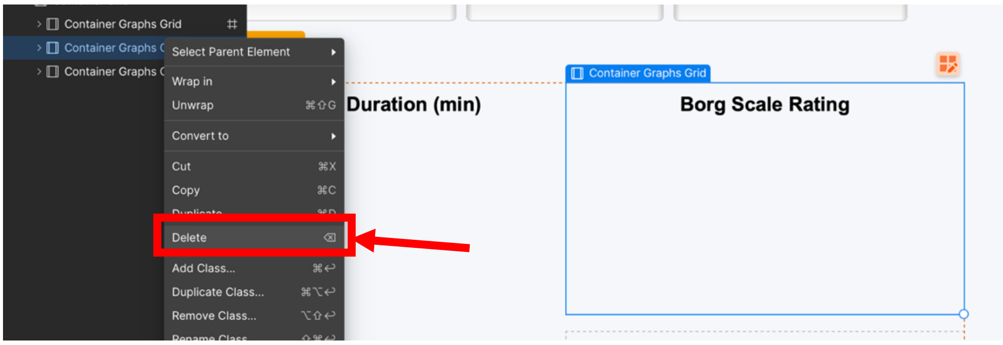

Figure A.73. Deleting a 'Container Graphs Grid'

9. Perfect. Now, let's delete the graph from the phone version of the website.
- On the **top menu**, switch the display view to **'Mobile (L)'** (Figure A.74).

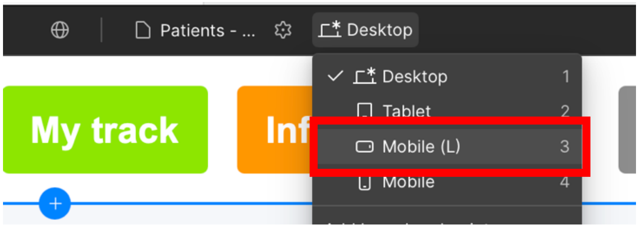

Figure A.74. Switching the display view to 'Mobile (L)'

10. In the Navigator panel:
- Click the **arrow icon (>)** next to **'Slider'** in the Navigator (Step 1 – Figure A.75).
- Then, click the **arrow icon (>)** next to **'Mask'** (Step 2 – Figure A.75).

You will see **three 'Slide _'**, each representing one of the mobile graphs:
- Slide 1 → 'Total Activity Duration (min)' graph
- Slide 2 → 'Borg Scale Rating' graph
- Slide 3 → 'Distance (*units*)' graph

11. **Right-click on the slide that matches the same graph you deleted in the desktop view** and select ***Delete*** (Step 3 – Figure A.75).

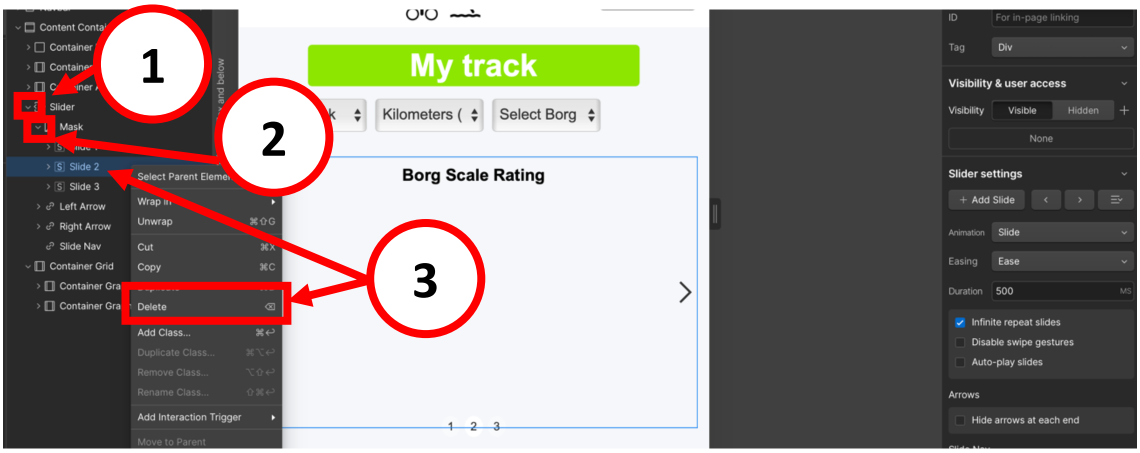

Figure A.75. Deleting a graph in mobile display view

12. **Click the 'X' next to 'Mobile (L)'** at the top of the screen to exit the mobile view.

13. Done! You have successfully removed the graph from both the desktop and mobile versions of the website.

14. **Publish the website** to apply and save your changes (For detailed instructions, refer to section *4. Publishing the Website*).

5.3. Deleting a filter

On the 'My track' page (patient's view) and on the 'My patients' page (staff view) there are different filters used to view and sort the data of graphs in different ways.

The following filters (Figure A.76 and Figure A.77) can be removed if they are not useful for your website:

- ***'Kilometres/Meters/Miles'* filter**
- ***'Borg Scale Range'* filter**

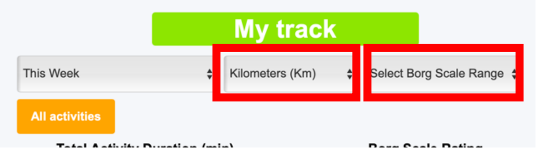

Figure A.76. Filters marked in red can be removed from the 'My track' page

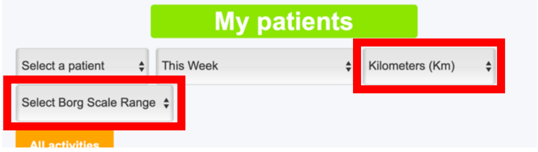

Figure A.77. Filters marked in red can be removed from the 'My patients' page

Important: The following filters cannot be deleted:

- *'Time filter (This Week, Last 2 Weeks, etc.)'* - appears in patients and staff pages.
- *'Select a patient'* filter - only appears in the staff page.

The next steps show how to remove a filter (**Figure A.76 and Figure A.77**):

1. In the left menu, click on the **'Pages' icon**.

2. Click on:

- ***Patients – My Track*** page: if you want to delete the filter from the **patient interface.**

- ***Staff – My Patients*** page: if you want to delete the filter from the **staff interface.**

- If you want to delete the filter from **both interfaces**: follow the next steps twice (first for the ***Patients – My Track*** page, and then for the ***Staff – My Patients*** page).

3. **Select the filter** you want to remove **by clicking on it** (Step 1 – Figure 78).

4. **Right-click** on it and select the ***Delete*** option (Step 2 – Figure A.78).

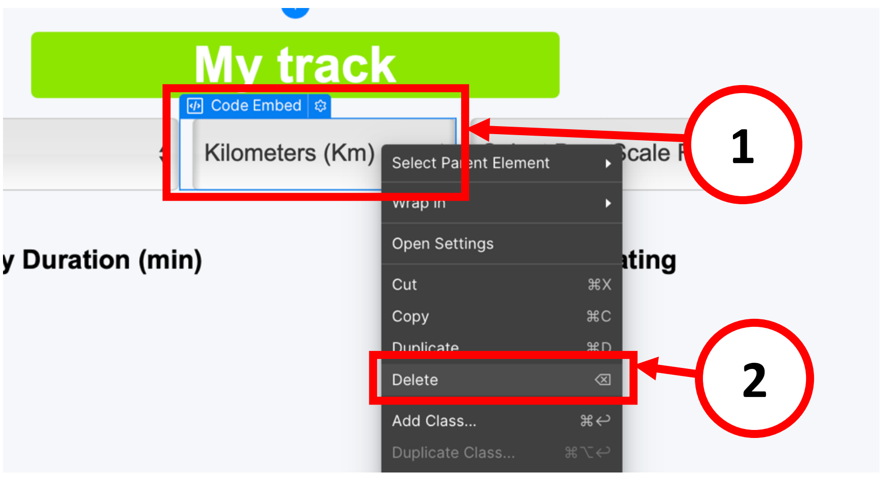

Figure A.78. Steps to delete a filter.

5. Done! The filter has been removed.

6. **Publish the website** to apply and save your changes (For detailed instructions, refer to section *4. Publishing the Website*).

Note: If you arrived here while following Example 2, click here to return to Example 2.

6. Deleting a User's Data (level of difficulty: **2**/5)

It's a good practice to conduct periodic reviews and remove patient information that is no longer needed to be stored online. Thus, this section shows how to delete a user's data.

For example:
If Patient 22 has completed their rehabilitation and staff members do no longer need to check their exercise data online, their data should be deleted from the database (from Xano). Or, if needed for other purposes, it can be saved locally (on a secure device) and then delete from the Cloud.

1. Log into **Xano**.

2. In the left menu, go to ***Database***.

3. Click on the **'data_entries'** table.

4. Select ***Filter*** (Step 1 – Figure A.79).

5. Click on ***+ Add filter*** (Step 2 – Figure A.79).

- Under Column, select ***Identification*** (Step 3 – Figure A.79).

- In **'Value'** field, **insert the ID of the patient** you would like to delete their entries. E.g. In our case, '22' (Step 3 – Figure A.79)

- Click ***Filter*** (Step 4 – Figure A.79)

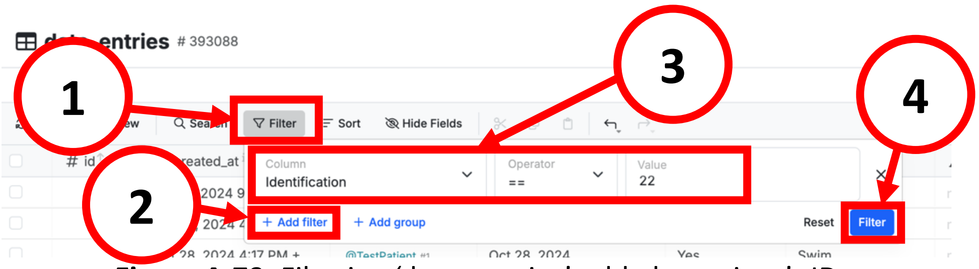

Figure A.79. Filtering 'data_entries' table by patient's ID

6. Now, only the data entries of this patient will appear on the screen.

- **Check the box next to '# id'** (Step 1 – Figure A.80) to select all entries of this patient.

- **If you prefer to store this data outside the database**:
  - Click ***Export*** (in blue – Figure A.80) to download the data as a CSV file.

  - Or, if you prefer to have the data in **Excel file**, log in to the published website as a staff member and download all data from this user from there.

- Delete the data by clicking ***Delete*** (Step 2 – Figure A.80)

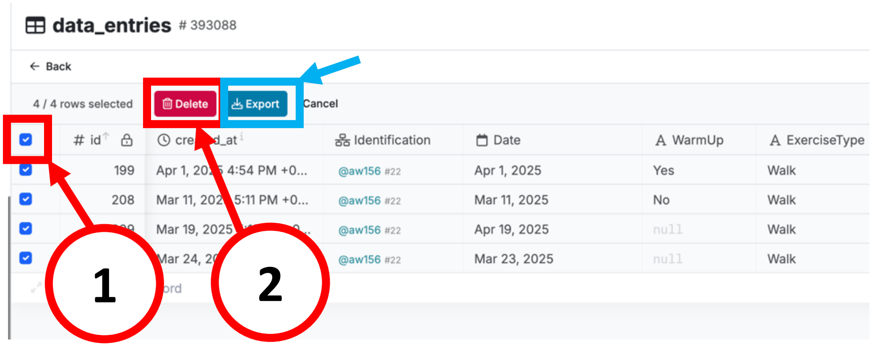

Figure A.80. Selecting all the filtered data entries and deleting them

7. Now, go back to ***Database*** (to the previous screen).

8. Click on the **'messages'** table.

9. Select ***Filter*** and click on ***+ Add filter***.
- Under Column, select ***sender_id***

- In **'Value'** field, **insert the ID of the patient** you would like to delete their sent messages entries. E.g. In our case, '22'.

- Click ***Filter***.

- **Check the box next to '# id'**, to select all sent messages of this patient.

- Delete them by clicking ***Delete***.

10. Change the filter by selecting ***receiver_id*** under Column.
- Apply it by clicking ***Filter***.

- **Check the box next to '# id'**, to select all the messages received by this patient.

- Click ***Delete***.

11. Remove the filter and go back to ***Database*** (to the previous screen).

12. Click on the **'user'** table.

13. **Locate the patient user you would like to delete:**
(In our case, Patient 22)

- **Check the box to the left** of the desired user's row (Step 1 – Figure A.81)
- Click ***Delete*** (Step 2 – Figure A.81)

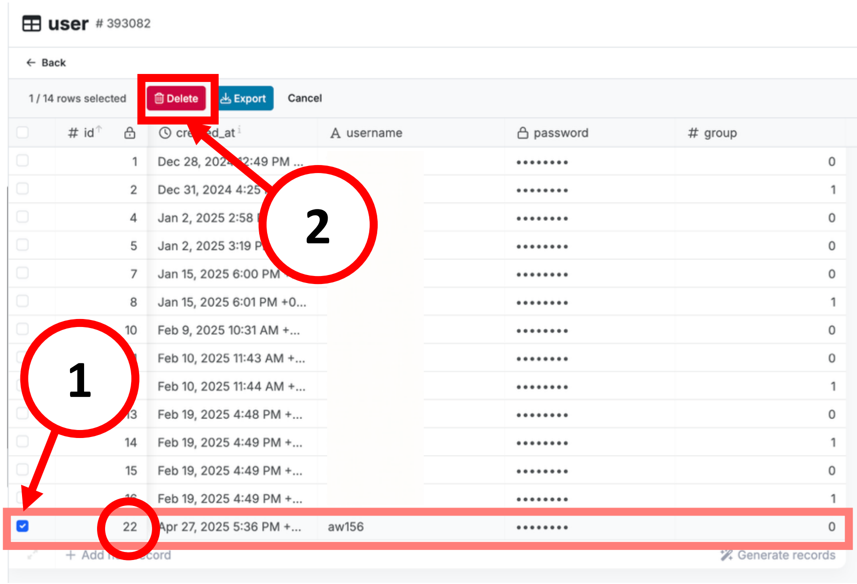

Figure A.81. Deleting a user in the 'user' table

14. **Done!** You have successfully deleted the exercise data entries, messages and username of this patient.
